# Supplementary figures and images for: Impact of COVID-19 lockdown on PM concentrations in an Italian Northern City: A year-by-year assessment
Source: PLoS One. 2022 Mar 28;17(3):e0263265. doi: 10.1371/journal.pone.0263265 (PMC8959169; doi:10.1371/journal.pone.0263265)

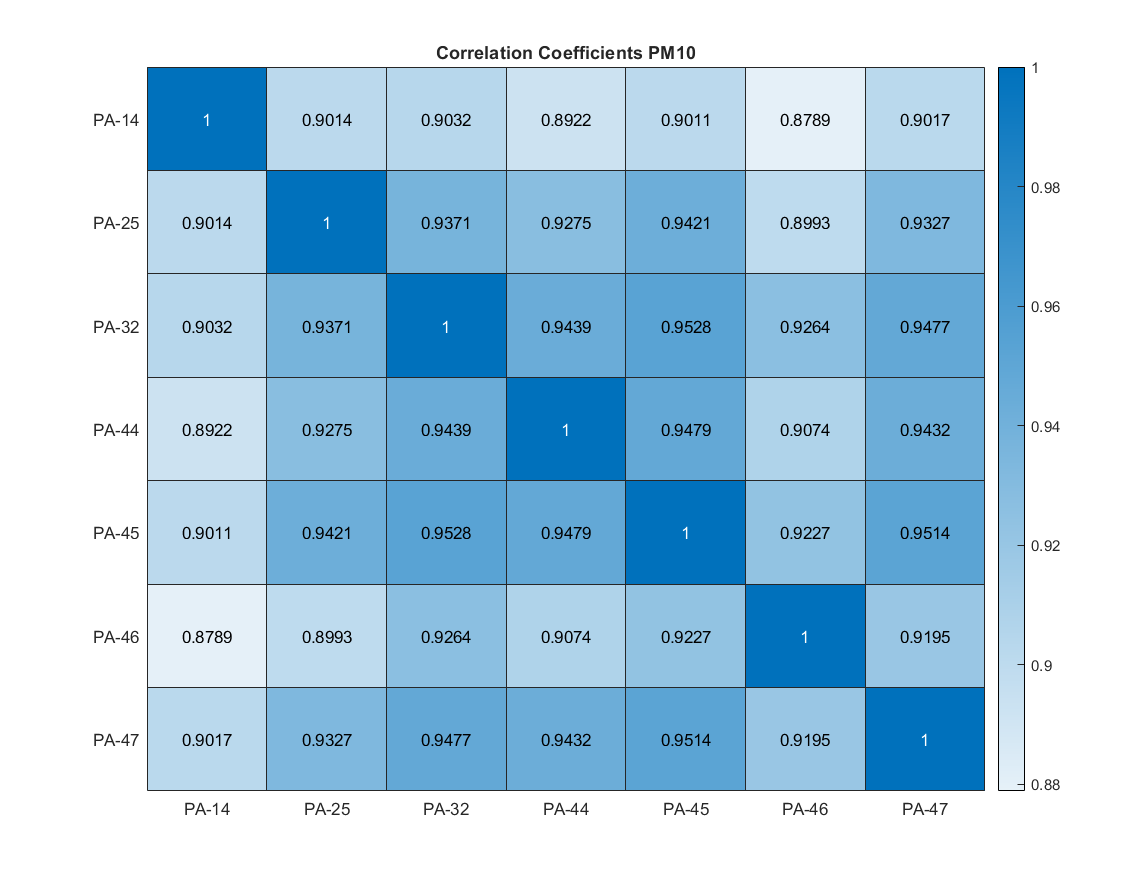

Supplement: S1 Fig — Correlation matrix (Pearson’s correlation) of the PM10 measurements performed by seven Purple Air sensors co-located on a selected day. (TIF) [file pone.0263265.s001.tif]

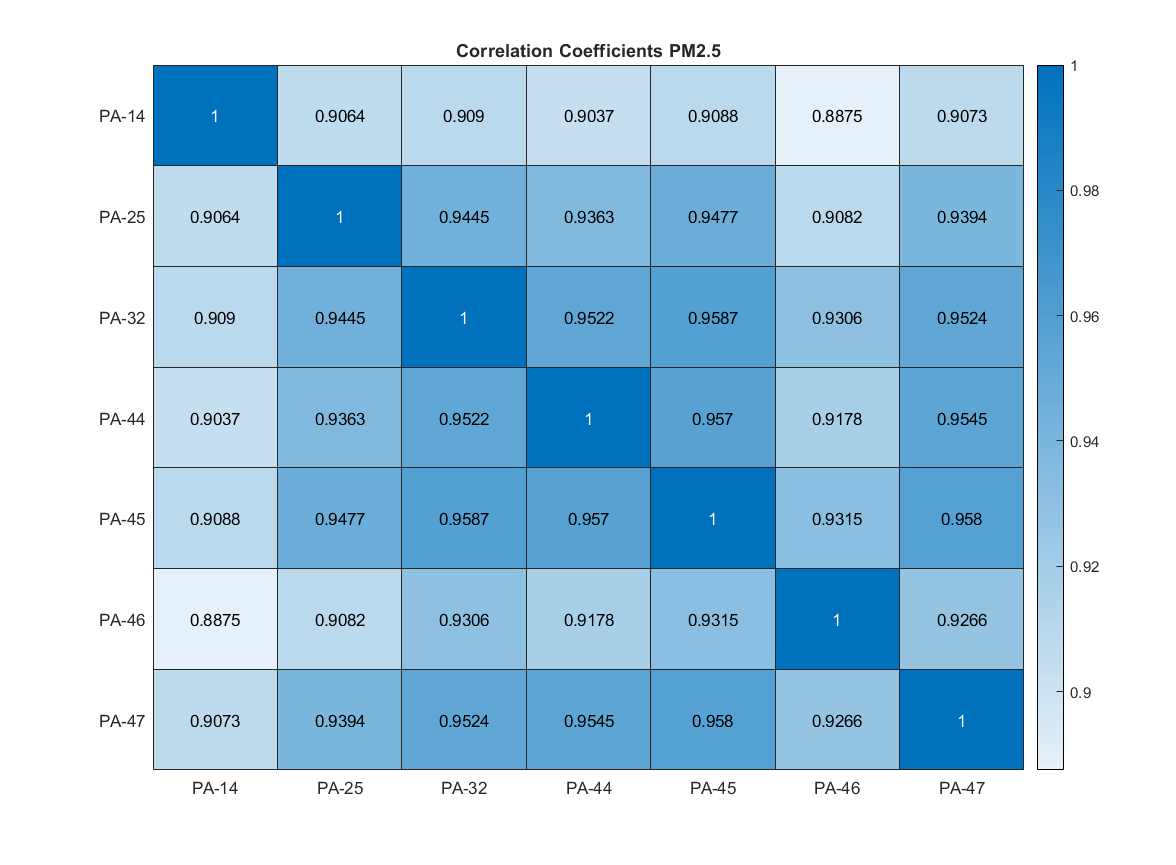

Supplement: S2 Fig — Correlation matrix (Pearson’s correlation) of the PM2.5 measurements performed by seven Purple Air sensors co-located on a selected day. (TIF) [file pone.0263265.s002.tif]

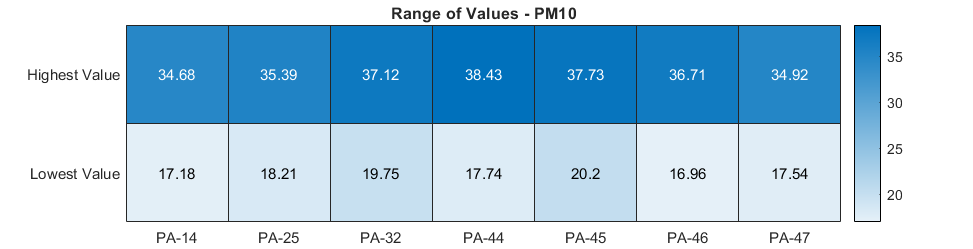

Supplement: S3 Fig — (TIF) [file pone.0263265.s003.tif]

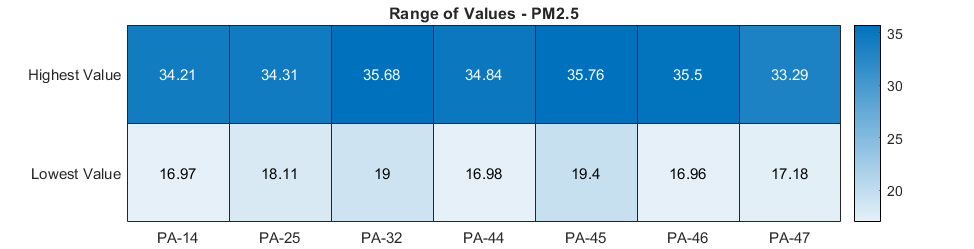

Supplement: S4 Fig — (TIF) [file pone.0263265.s004.tif]

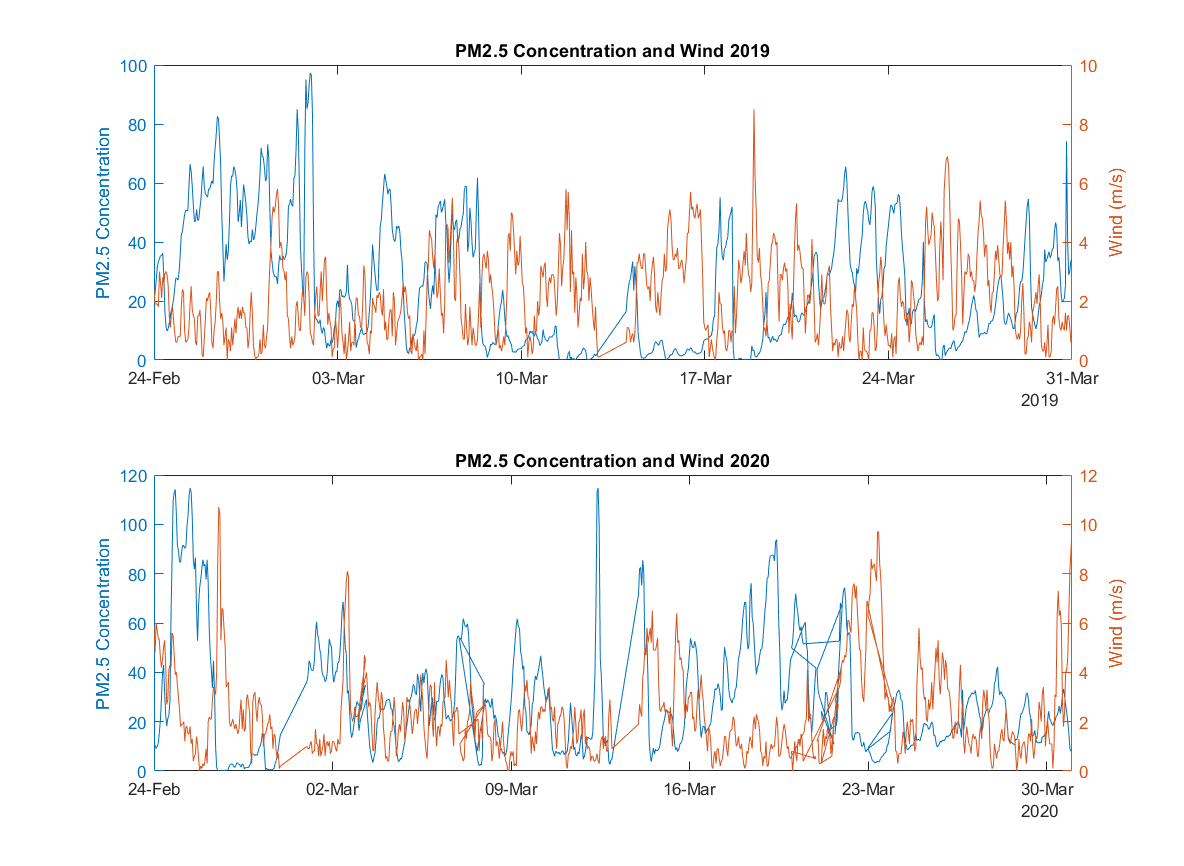

Supplement: S5 Fig — The upper plot shows 2019 data, the lower plot shows 2020 data. (TIF) [file pone.0263265.s005.tif]

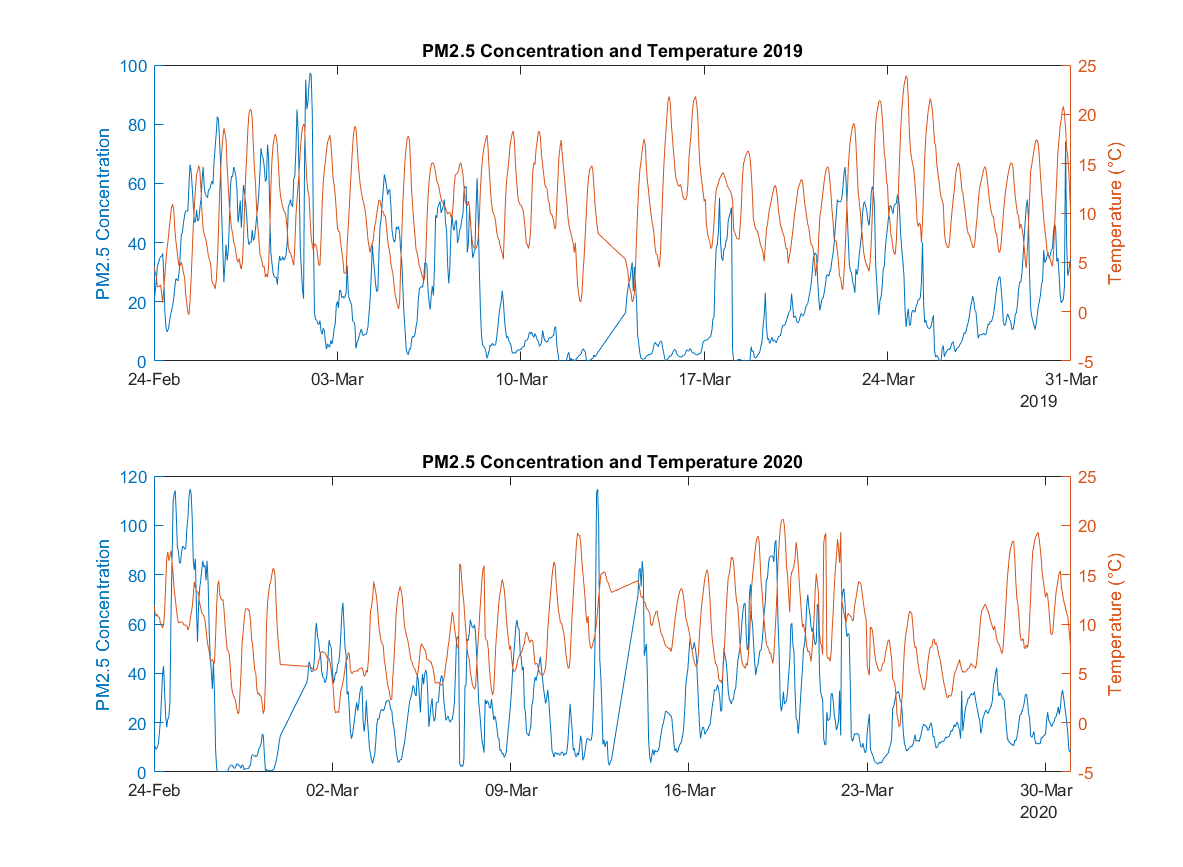

Supplement: S6 Fig — (TIF) [file pone.0263265.s006.tif]

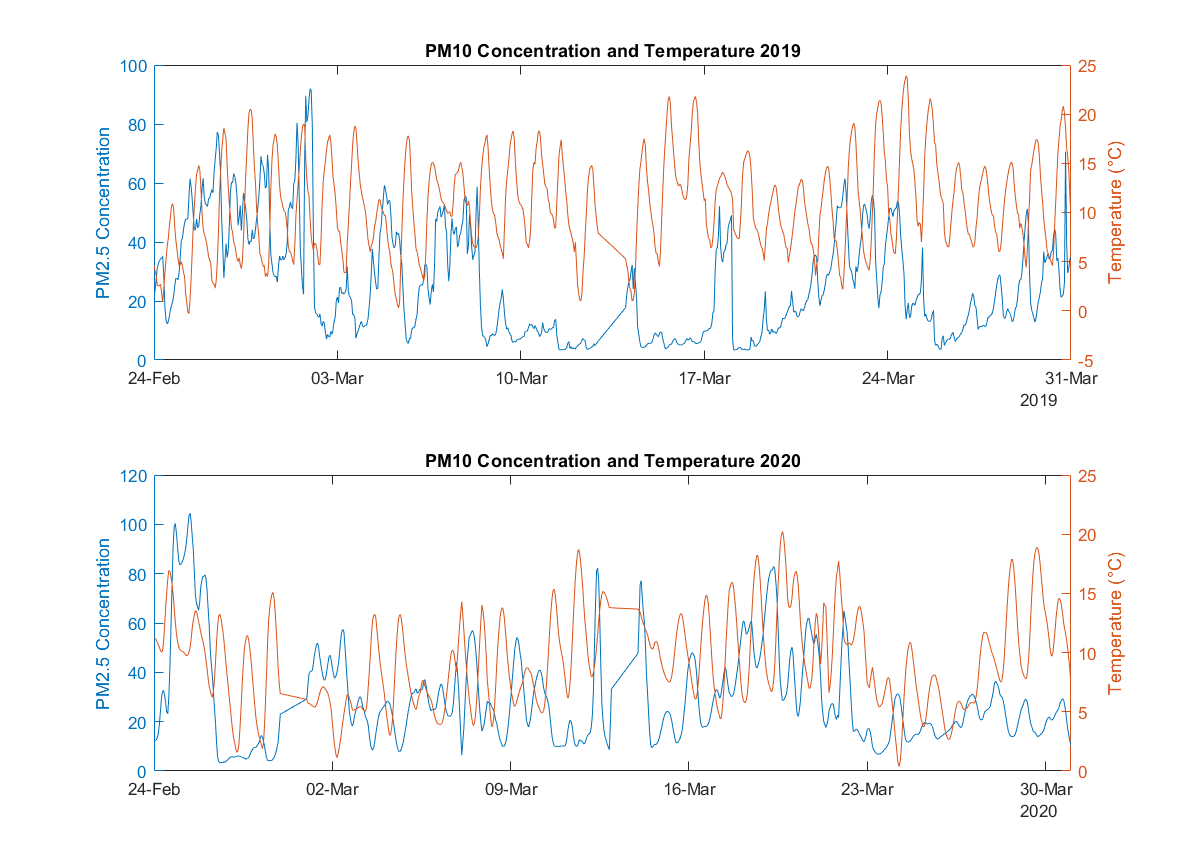

Supplement: S7 Fig — The upper plot shows 2019 data, the lower plot shows 2020 data. (TIF) [file pone.0263265.s007.tif]

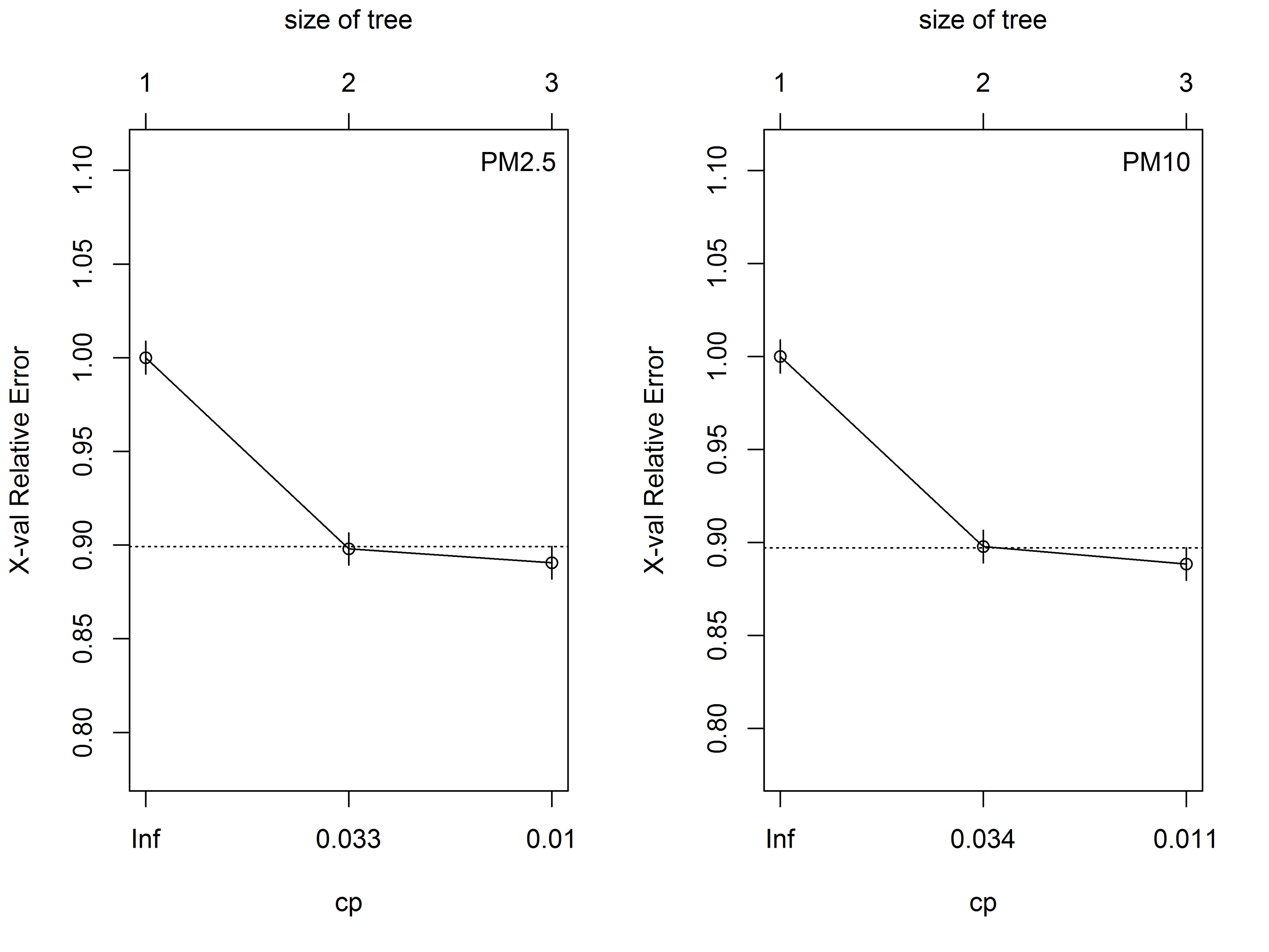

Supplement: S8 Fig — The x-axis represents the complexity parameter corresponding to different tree sizes while the y-axis represents the cross validation relative error. (TIF) [file pone.0263265.s008.tif]

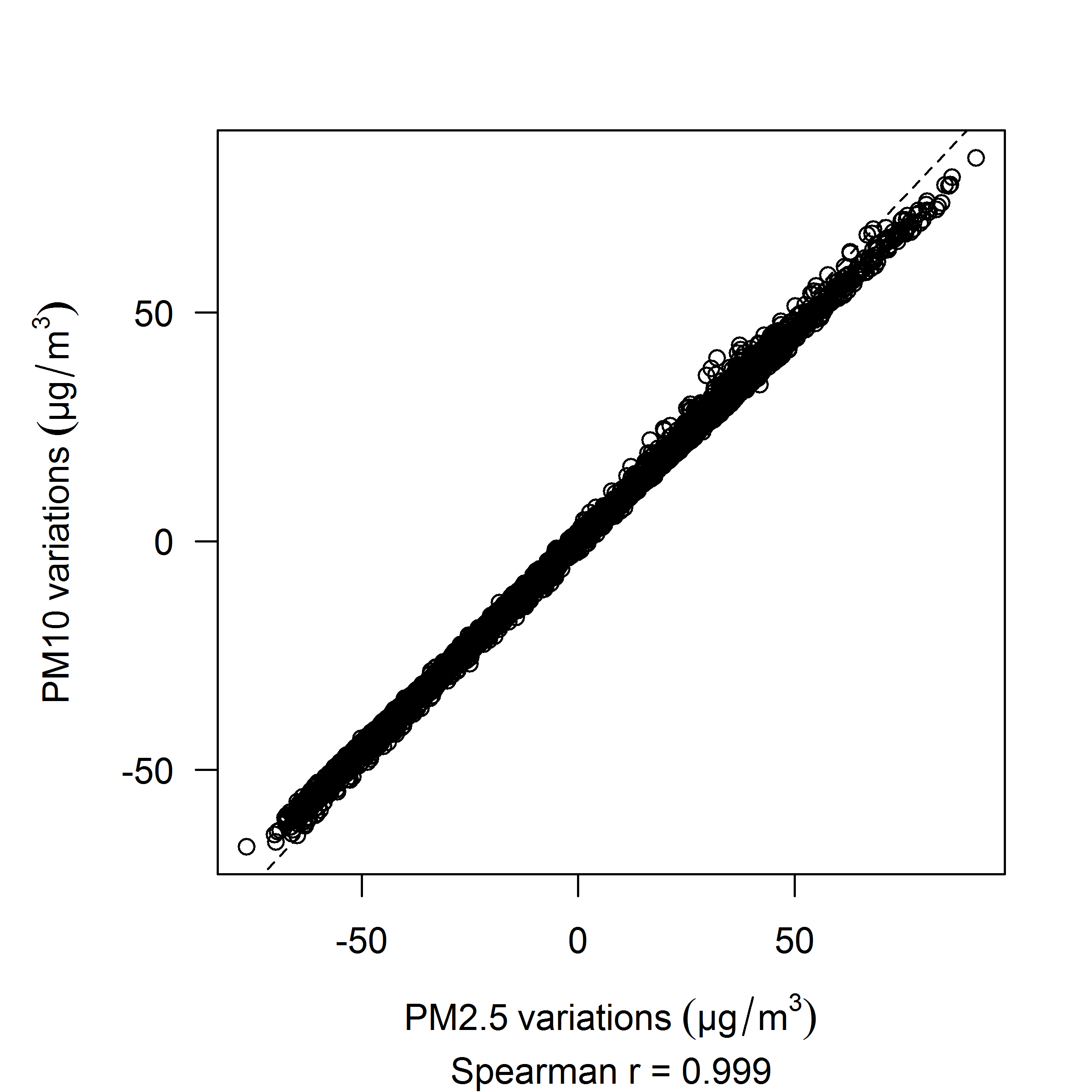

Supplement: S9 Fig — (TIF) [file pone.0263265.s009.tif]

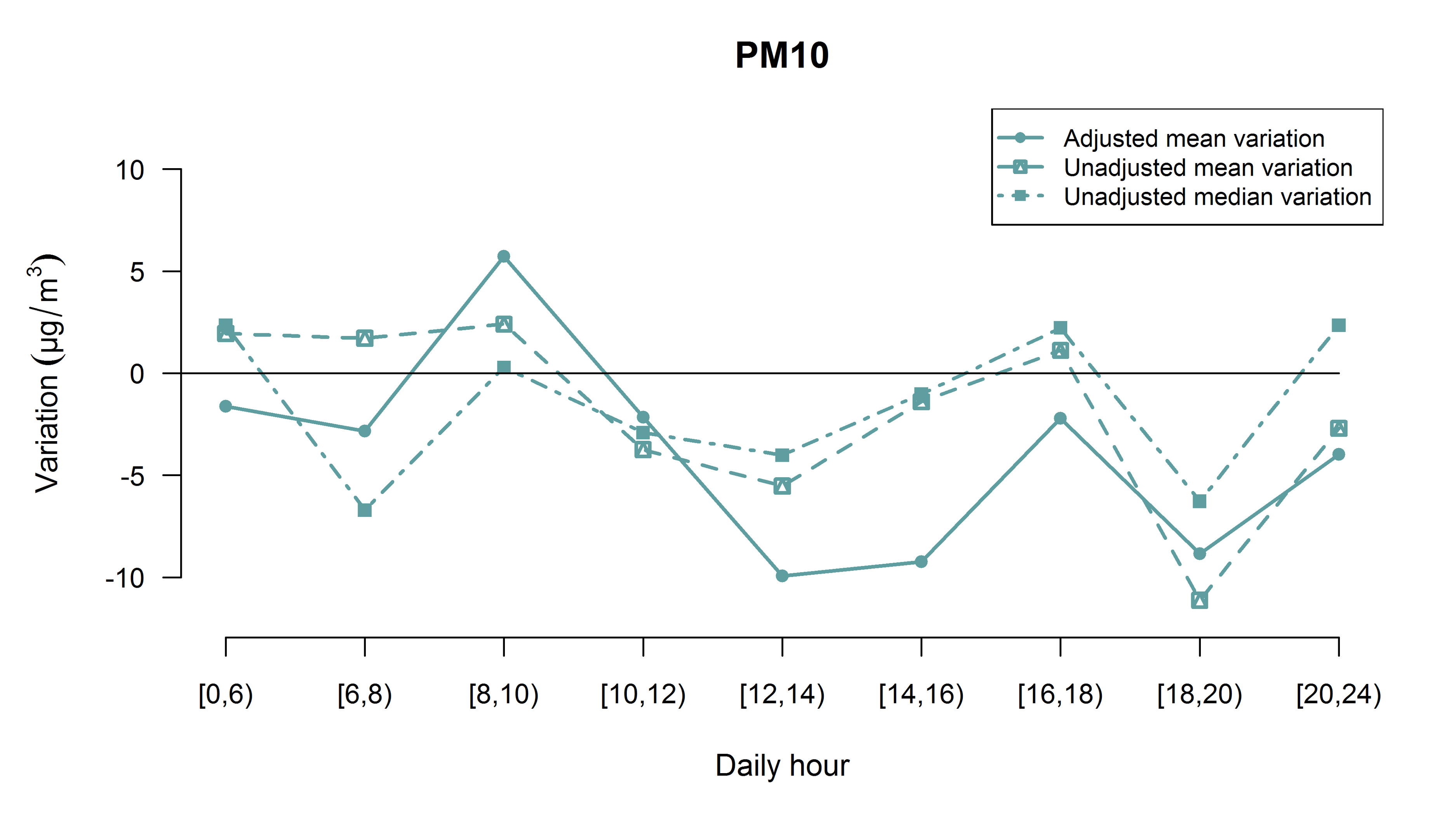

Supplement: S10 Fig — Data are presented as adjusted mean variations, unadjusted mean variations and unadjusted median variations. (TIF) [file pone.0263265.s010.tif]

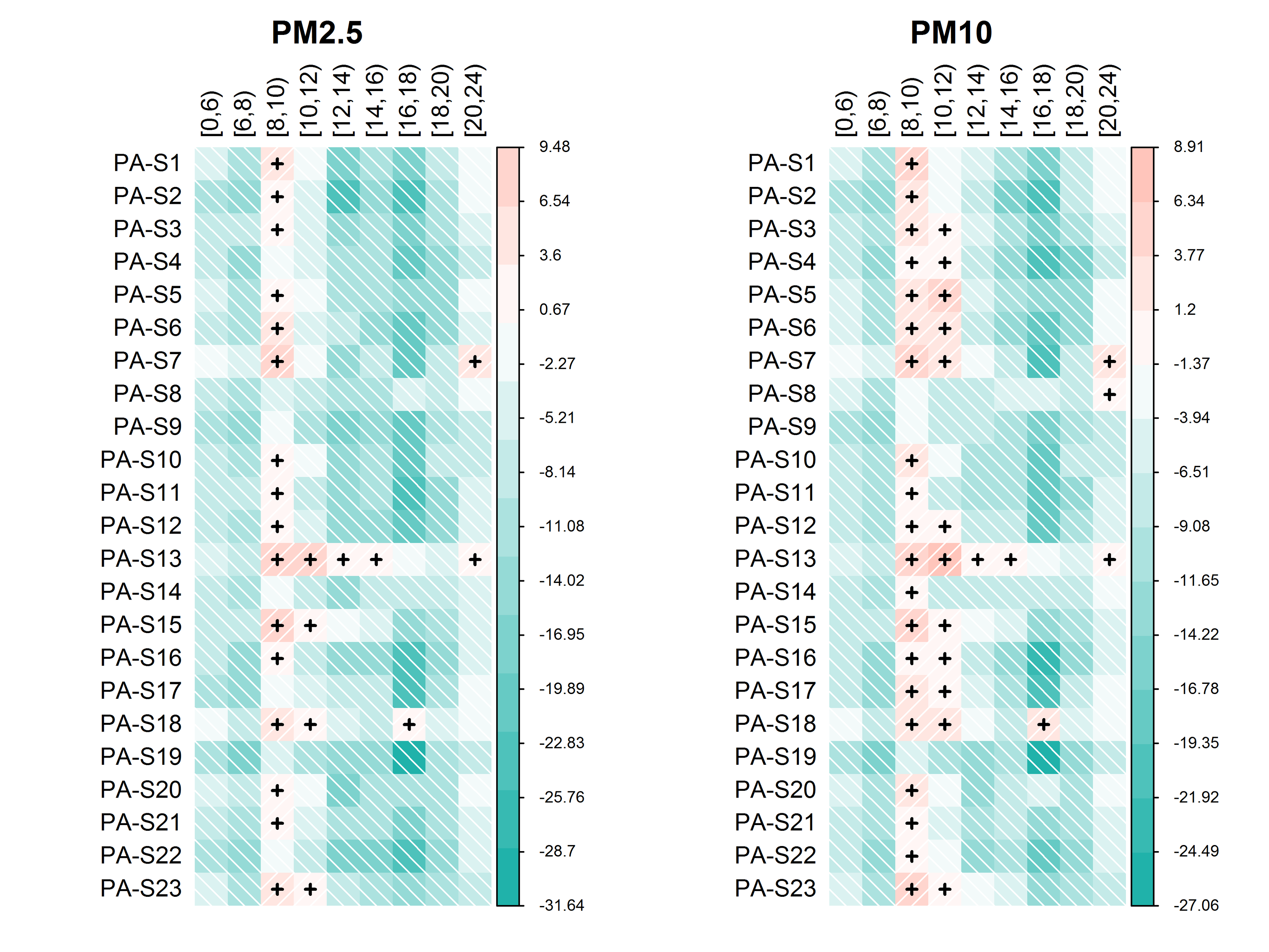

Supplement: S11 Fig — Each heatmap row graphically represents the median absolute differences between observed and predicted pollutants measurements by daily hours intervals. Shades of green indicate negative absolute differences between observed and predicted pollutants concentration during 2020, shades of red indicate positive absolute differences between observed and predicted pollutants concentration during 2020 as showed by the colour code legend on the right side of each plot. The “+” symbol denotes a positive absolute differences between observed and predicted pollutants concentration. (TIF) [file pone.0263265.s011.tif]
