# Supplementary material for: Impact of COVID-19 lockdown on PM concentrations in an Italian Northern City: A year-by-year assessment
Source: PLoS One. 2022 Mar 28;17(3):e0263265. doi: 10.1371/journal.pone.0263265 (PMC8959169; doi:10.1371/journal.pone.0263265)
Supplement: S1 Table — (DOCX) [file pone.0263265.s012.docx]

|  | **Hour** | | | | | | | | | |
| --- | --- | --- | --- | --- | --- | --- | --- | --- | --- | --- |
|  | **[0,6)** | **[6,8)** | **[8,10)** | **[10,12)** | **[12,14)** | **[14,16)** | **[16,18)** | **[18,20)** | **[20,24)** | **TOTAL** |
| ***All sensors*** | ***1872*** | ***776*** | ***549*** | ***295*** | ***340*** | ***295*** | ***228*** | ***297*** | ***800*** | ***5452*** |
| PA-S1 | 82 | 34 | 24 | 13 | 15 | 13 | 10 | 13 | 34 | 238 |
| PA-S2 | 78 | 32 | 24 | 11 | 14 | 13 | 9 | 12 | 34 | 227 |
| PA-S3 | 82 | 34 | 24 | 13 | 15 | 13 | 10 | 13 | 35 | 239 |
| PA-S4 | 82 | 34 | 24 | 13 | 15 | 13 | 10 | 13 | 35 | 239 |
| PA-S5 | 81 | 34 | 24 | 13 | 15 | 13 | 10 | 13 | 35 | 238 |
| PA-S6 | 82 | 34 | 24 | 13 | 15 | 13 | 10 | 13 | 35 | 239 |
| PA-S7 | 82 | 34 | 24 | 13 | 15 | 13 | 10 | 13 | 35 | 239 |
| PA-S8 | 82 | 34 | 24 | 13 | 15 | 13 | 10 | 13 | 35 | 239 |
| PA-S9 | 82 | 34 | 24 | 13 | 15 | 13 | 10 | 13 | 35 | 239 |
| PA-S10 | 82 | 34 | 24 | 13 | 15 | 13 | 10 | 13 | 35 | 239 |
| PA-S11 | 82 | 34 | 24 | 13 | 15 | 13 | 10 | 13 | 35 | 239 |
| PA-S12 | 82 | 34 | 24 | 13 | 15 | 13 | 10 | 13 | 35 | 239 |
| PA-S13 | 82 | 34 | 23 | 11 | 13 | 11 | 9 | 13 | 35 | 231 |
| PA-S14 | 82 | 34 | 24 | 13 | 15 | 13 | 10 | 13 | 35 | 239 |
| PA-S15 | 82 | 34 | 24 | 13 | 15 | 13 | 10 | 13 | 35 | 239 |
| PA-S16 | 82 | 34 | 24 | 13 | 15 | 13 | 10 | 13 | 35 | 239 |
| PA-S17 | 82 | 34 | 24 | 13 | 15 | 13 | 10 | 13 | 35 | 239 |
| PA-S18 | 73 | 30 | 22 | 13 | 13 | 12 | 10 | 12 | 33 | 218 |
| PA-S19 | 82 | 34 | 24 | 13 | 15 | 12 | 10 | 13 | 34 | 237 |
| PA-S20 | 82 | 34 | 24 | 13 | 15 | 13 | 10 | 13 | 35 | 239 |
| PA-S21 | 82 | 34 | 24 | 13 | 15 | 13 | 10 | 13 | 35 | 239 |
| PA-S22 | 82 | 34 | 24 | 13 | 15 | 13 | 10 | 13 | 35 | 239 |
| PA-S23 | 82 | 34 | 24 | 13 | 15 | 13 | 10 | 13 | 35 | 239 |

**S1 Table. Number of paired measurements available by sensor and hours**.
