# Supplementary material for: Impact of COVID-19 lockdown on PM concentrations in an Italian Northern City: A year-by-year assessment
Source: PLoS One. 2022 Mar 28;17(3):e0263265. doi: 10.1371/journal.pone.0263265 (PMC8959169; doi:10.1371/journal.pone.0263265)
Supplement: S2 Table — Each PurpleAir (PA) ID corresponds to a different sensor. In green: reduction in terms of PM2.5 between 2019 and 2020; in red: increase in terms of PM2.5 between 2019 and 2020. (DOCX) [file pone.0263265.s013.docx]

|  | **Adjusted mean PM2.5 variations by hour** | | | | | | | | |
| --- | --- | --- | --- | --- | --- | --- | --- | --- | --- |
| **Sensor** | **[0,6)** | **[6,8)** | **[8,10)** | **[10,12)** | **[12,14)** | **[14,16)** | **[16,18)** | **[18,20)** | **[20,24)** |
| ***All sensors*** | ***-1.77*** | ***-3.13*** | ***6.11*** | ***-2.52*** | ***-11.20*** | ***-10.54*** | ***-3.01*** | ***-10.37*** | ***-4.63*** |
| PA-S1 | 1.20 | -1.30 | 10.30 | -0.10 | -11.18 | -10.47 | -1.67 | -9.93 | -2.90 |
| PA-S2 | -2.33 | -3.55 | 6.69 | -2.08 | -12.76 | -10.82 | -6.48 | -8.69 | -4.68 |
| PA-S3 | -1.02 | -0.72 | 8.03 | -0.07 | -9.79 | -9.25 | -4.24 | -9.82 | -6.52 |
| PA-S4 | -2.70 | -4.92 | 4.46 | 0.60 | -8.59 | -10.19 | -3.77 | -12.56 | -6.24 |
| PA-S5 | 1.33 | -1.20 | 10.22 | 4.07 | -5.70 | -6.79 | -1.11 | -8.87 | -2.13 |
| PA-S6 | -0.82 | -3.04 | 7.00 | 2.50 | -7.44 | -9.19 | -2.58 | -10.86 | -4.33 |
| PA-S7 | 4.94 | 3.13 | 12.23 | 2.45 | -9.09 | -7.67 | -1.40 | -7.73 | 1.50 |
| PA-S8 | -2.54 | -4.03 | 4.37 | -4.32 | -12.88 | -10.62 | 1.42 | -6.96 | -2.09 |
| PA-S9 | -8.31 | -9.21 | -2.20 | -10.99 | -14.53 | -12.49 | -5.88 | -14.63 | -11.48 |
| PA-S10 | -2.37 | -3.23 | 4.12 | -5.63 | -12.09 | -10.93 | -2.54 | -10.53 | -6.39 |
| PA-S11 | -0.76 | -0.90 | 7.20 | -4.35 | -14.15 | -10.42 | -2.79 | -11.08 | -5.70 |
| PA-S12 | -2.45 | -5.45 | 5.46 | -2.66 | -11.66 | -12.53 | -5.78 | -12.76 | -5.31 |
| PA-S13 | -2.52 | -2.90 | 7.08 | 0.64 | -8.48 | -10.25 | -2.82 | -12.38 | -3.22 |
| PA-S14 | -3.35 | -3.24 | 6.64 | -2.13 | -9.86 | -9.56 | -0.02 | -7.59 | -1.35 |
| PA-S15 | -0.82 | 0.32 | 9.22 | -0.17 | -7.81 | -7.75 | -2.04 | -3.60 | -5.60 |
| PA-S16 | -1.82 | -3.35 | 6.29 | 1.22 | -8.39 | -11.82 | -7.65 | -10.88 | -6.86 |
| PA-S17 | -2.15 | -3.80 | 5.04 | -0.96 | -9.62 | -8.43 | -4.40 | -9.69 | -5.13 |
| PA-S18 | -1.35 | -2.43 | 5.96 | -6.90 | -12.22 | -12.28 | 1.60 | -10.96 | -2.72 |
| PA-S19 | -4.25 | -6.26 | 2.56 | -4.16 | -12.98 | -12.89 | -6.76 | -10.64 | -7.56 |
| PA-S20 | -1.35 | -2.95 | 5.79 | -5.62 | -14.68 | -11.84 | -0.51 | -12.54 | -1.27 |
| PA-S21 | -1.90 | -3.27 | 5.73 | -5.90 | -13.88 | -11.17 | -1.47 | -9.89 | -2.71 |
| PA-S22 | -4.57 | -6.55 | 1.60 | -9.32 | -16.98 | -14.89 | -5.97 | -14.01 | -7.78 |
| PA-S23 | -0.91 | -3.30 | 6.47 | -3.86 | -13.03 | -10.65 | -3.20 | -11.80 | -5.82 |

**S2 Table. Adjusted mean variations in terms of PM2.5 between 2019 and 2020 by sensor and daily hours**. Each PurpleAir (PA) ID corresponds to a different sensor. In green: reduction in terms of PM2.5 between 2019 and 2020; in red: increase in terms of PM2.5 between 2019 and 2020.
