# Supplementary material for: Impact of COVID-19 lockdown on PM concentrations in an Italian Northern City: A year-by-year assessment
Source: PLoS One. 2022 Mar 28;17(3):e0263265. doi: 10.1371/journal.pone.0263265 (PMC8959169; doi:10.1371/journal.pone.0263265)
Supplement: S3 Table — Each Purple Air (PA) ID corresponds to a different sensor. In green: reduction in terms of PM10 between 2019 and 2020; in red: increase in terms of PM10 between 2019 and 2020. (DOCX) [file pone.0263265.s014.docx]

|  | **Adjusted mean PM10 variations by hour** | | | | | | | | |
| --- | --- | --- | --- | --- | --- | --- | --- | --- | --- |
| **Sensor** | **[0,6)** | **[6,8)** | **[8,10)** | **[10,12)** | **[12,14)** | **[14,16)** | **[16,18)** | **[18,20)** | **[20,24)** |
| ***All sensors*** | ***-1.62*** | ***-2.83*** | ***5.72*** | ***-2.16*** | ***-9.94*** | ***-9.24*** | ***-2.21*** | ***-8.84*** | ***-3.98*** |
| PA-S1 | 1.00 | -1.12 | 9.07 | -0.24 | -9.85 | -9.11 | -1.25 | -8.57 | -2.58 |
| PA-S2 | -2.28 | -3.16 | 6.08 | -2.13 | -11.93 | -9.78 | -5.59 | -7.70 | -4.18 |
| PA-S3 | -1.09 | -0.89 | 7.22 | 0.00 | -8.40 | -7.80 | -3.31 | -8.21 | -5.63 |
| PA-S4 | -2.82 | -4.75 | 4.31 | 0.89 | -7.49 | -9.16 | -2.79 | -11.01 | -5.62 |
| PA-S5 | 1.16 | -1.03 | 9.39 | 3.77 | -4.85 | -5.74 | -0.49 | -7.34 | -1.63 |
| PA-S6 | -0.51 | -2.48 | 6.61 | 2.55 | -6.38 | -7.85 | -1.69 | -9.23 | -3.53 |
| PA-S7 | 4.05 | 2.61 | 10.89 | 1.80 | -8.46 | -6.85 | -1.01 | -6.99 | 1.16 |
| PA-S8 | -2.60 | -4.00 | 3.68 | -4.24 | -12.05 | -9.79 | 1.73 | -5.83 | -2.03 |
| PA-S9 | -7.20 | -8.06 | -1.36 | -9.43 | -12.64 | -10.88 | -4.59 | -12.37 | -9.88 |
| PA-S10 | -1.86 | -2.73 | 4.21 | -4.40 | -10.23 | -9.28 | -1.78 | -8.75 | -5.13 |
| PA-S11 | -0.58 | -0.80 | 6.83 | -3.64 | -12.26 | -8.86 | -1.94 | -9.18 | -4.80 |
| PA-S12 | -2.06 | -4.68 | 5.40 | -2.11 | -10.34 | -11.15 | -4.64 | -11.02 | -4.46 |
| PA-S13 | -2.11 | -2.39 | 6.35 | 0.72 | -7.38 | -8.66 | -2.13 | -10.55 | -2.68 |
| PA-S14 | -3.25 | -3.20 | 6.25 | -1.83 | -8.84 | -8.49 | 0.58 | -6.25 | -1.13 |
| PA-S15 | -0.97 | -0.04 | 7.95 | -0.47 | -7.27 | -6.97 | -1.93 | -3.53 | -5.09 |
| PA-S16 | -1.57 | -2.92 | 5.90 | 1.21 | -7.44 | -10.49 | -6.54 | -9.39 | -6.05 |
| PA-S17 | -2.03 | -3.47 | 4.84 | -0.52 | -8.42 | -7.38 | -3.53 | -8.20 | -4.49 |
| PA-S18 | -1.39 | -2.18 | 5.25 | -6.14 | -11.09 | -10.94 | 1.75 | -9.57 | -2.63 |
| PA-S19 | -3.92 | -5.75 | 2.57 | -3.70 | -11.55 | -11.34 | -5.60 | -8.96 | -6.69 |
| PA-S20 | -1.22 | -2.65 | 5.44 | -4.96 | -12.78 | -10.10 | 0.22 | -10.75 | -0.89 |
| PA-S21 | -1.81 | -3.11 | 5.45 | -5.26 | -12.44 | -9.86 | -0.72 | -8.32 | -2.23 |
| PA-S22 | -3.36 | -5.32 | 2.97 | -7.82 | -15.18 | -13.04 | -4.09 | -11.49 | -6.08 |
| PA-S23 | -0.89 | -3.06 | 6.07 | -3.49 | -11.47 | -9.20 | -2.28 | -10.03 | -5.18 |

**S3 Table. Adjusted median variations in terms of PM10 between 2019 and 2020 by sensor and daily hours**. Each Purple Air (PA) ID corresponds to a different sensor. In green: reduction in terms of PM10 between 2019 and 2020; in red: increase in terms of PM10 between 2019 and 2020.
