# Supplementary material for: Impact of COVID-19 lockdown on PM concentrations in an Italian Northern City: A year-by-year assessment
Source: PLoS One. 2022 Mar 28;17(3):e0263265. doi: 10.1371/journal.pone.0263265 (PMC8959169; doi:10.1371/journal.pone.0263265)
Supplement: S4 Table — (DOCX) [file pone.0263265.s015.docx]

| Pollutant | Index | LMM |
| --- | --- | --- |
| PM2.5 | RMSE | 23.93 |
| PM2.5 | MAE | 18.94 |
| PM2.5 | *r* | 0.51 |
| PM10 | RMSE | 21.64 |
| PM10 | MAE | 17.15 |
| PM10 | *r* | 0.52 |

**S4 Table.** Root mean square error, mean absolute error and Pearson correlation coefficient r from the LMM regression method.
