# Supplementary material for: Impact of COVID-19 lockdown on PM concentrations in an Italian Northern City: A year-by-year assessment
Source: PLoS One. 2022 Mar 28;17(3):e0263265. doi: 10.1371/journal.pone.0263265 (PMC8959169; doi:10.1371/journal.pone.0263265)
Supplement: S1 File — (DOCX) [file pone.0263265.s016.docx]

**Support Information**

**Methods**

An alternative approach based on the method proposed by Venter et al. [1] has been applied to estimate the potential impact of the lock-down on pollutants concentration during year 2020.

The method consists of the following steps:

1. Train linear mixed model regression models (LMM) using data from 2019. Analyses are performed at a sensor level, using PM2.5 and PM10 levels in turn as dependent variable and the following predictors as independent variables: working day, wind, temperature, humidity, precipitations, solar radiation, day/month, daily hour categories ([0,6), [6,8), [8,10), [10,12), [12,14), [14,16), [16,18), [18,20), [20,24)). The hour category information is used as random effect grouping variable, while the remaining ones as fixed terms.
2. Apply the LMM model trained on data from 2019 to forecast PM2.5 and PM10 concentrations during 2020.
3. Using the predicted pollutants concentration as benchmark, compare the predicted and the observed PM2.5 and PM10 values to estimate the absolute change (observed – predicted) in terms of pollutants concentration. Positive changes indicate an increase in terms of pollutants concentration compared to the expected values, negative changes a decrease in terms of pollutants concentration compared to the expected values. Positive and negative changes can be attributed to the COVID-19 related lockdown.

Linear mixed model analysis was performed by the *lmer* function implemented in the R package called *lme4*.

**Results**

The median Root Mean Square Error (RMSE), median Mean Absolute Error (MAE) and median Pearson correlation coefficient *r* over the 23 sensors analysed are reported in **S4 Table**. The median difference between observed and predicted PM2.5 and PM10 pollutants concentration by sensor and daily hour are reported in **S4 Fig**.

**Supplementary Figures**

**
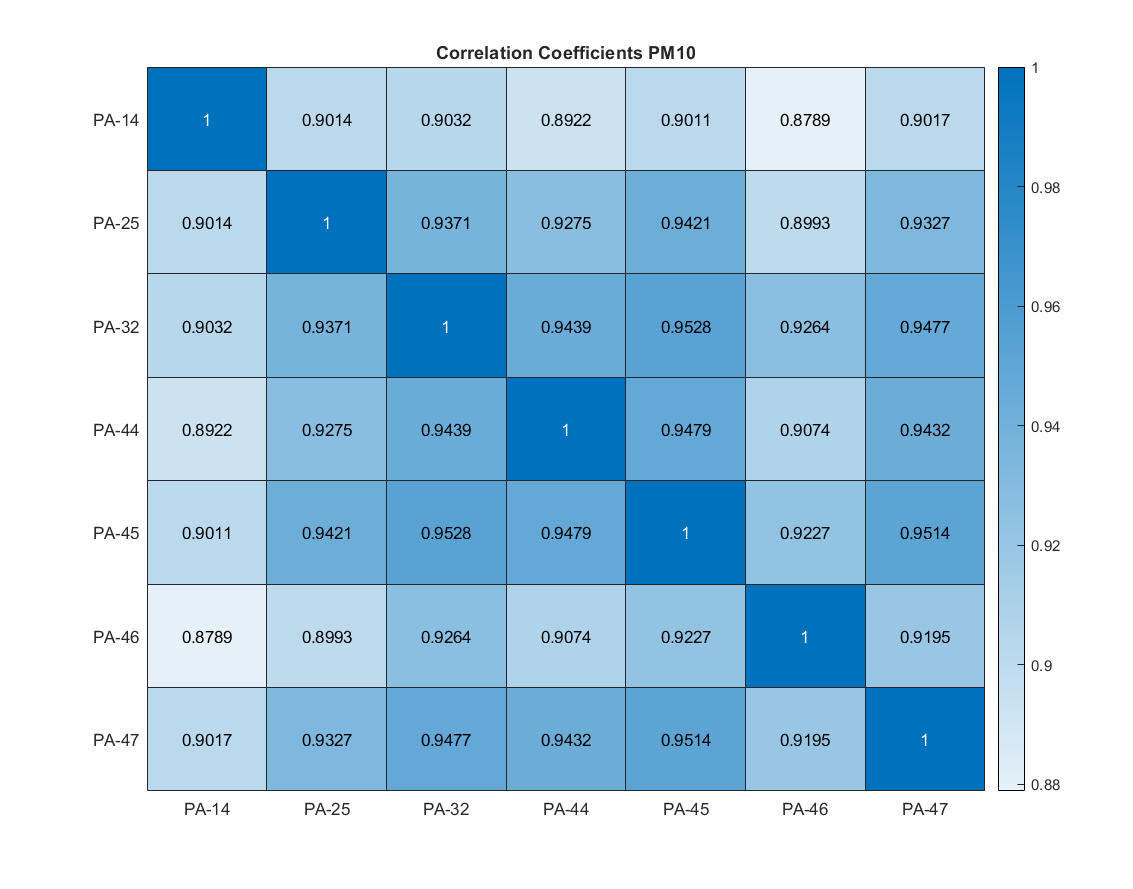
**

**S1 Fig. PM10 correlation matrix.** Correlation matrix (Pearson’s correlation) of the PM10 measurements performed by seven Purple Air sensors co-located on a selected day.

**
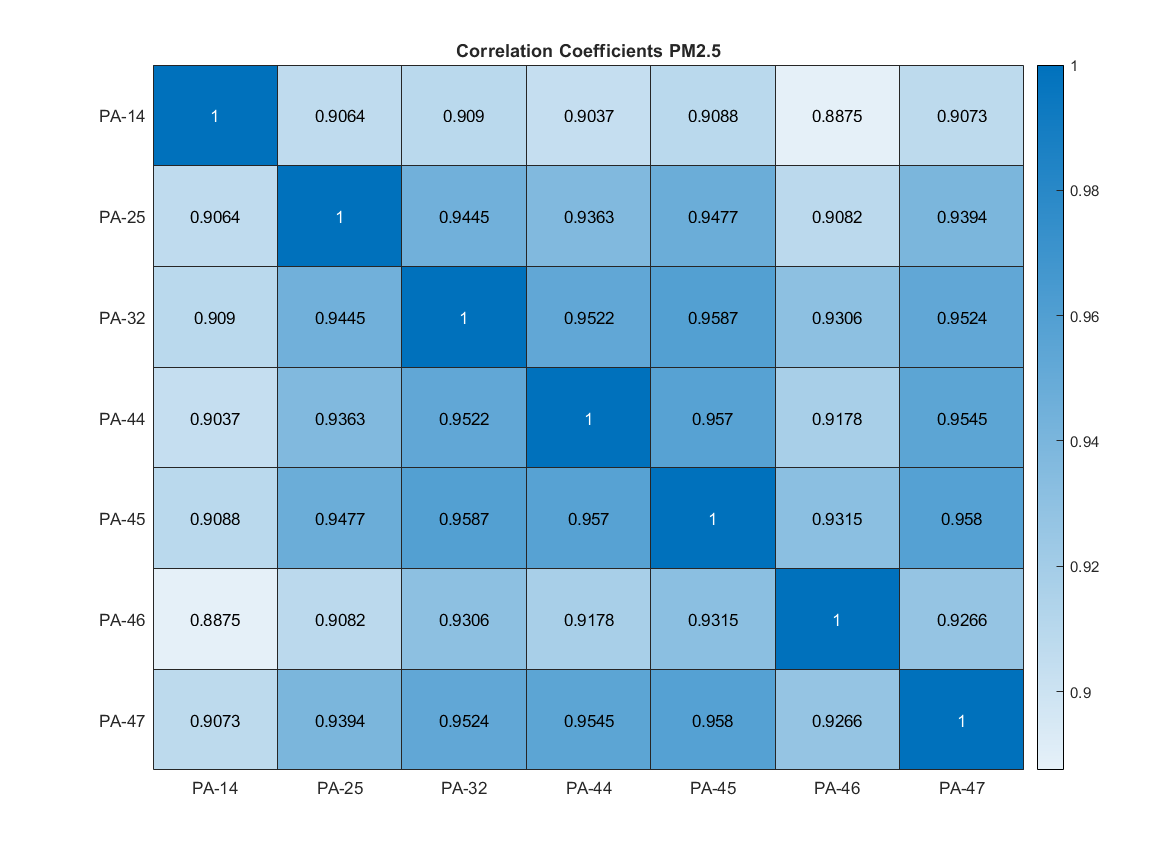
**

**S2 Fig. PM2.5 correlation matrix.** Correlation matrix (Pearson’s correlation) of the PM2.5 measurements performed by seven Purple Air sensors co-located on a selected day.


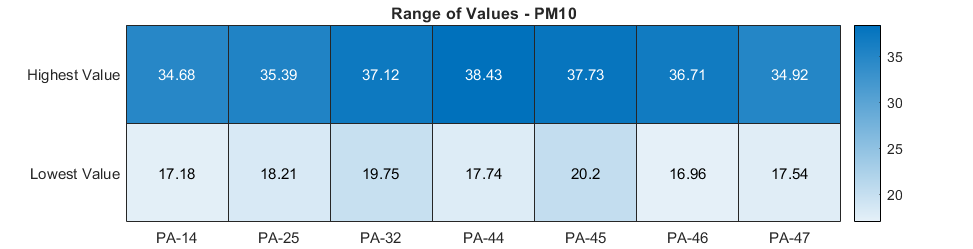


**S3 Fig.** Range of the PM10 values measured by the seven co-located sensors.


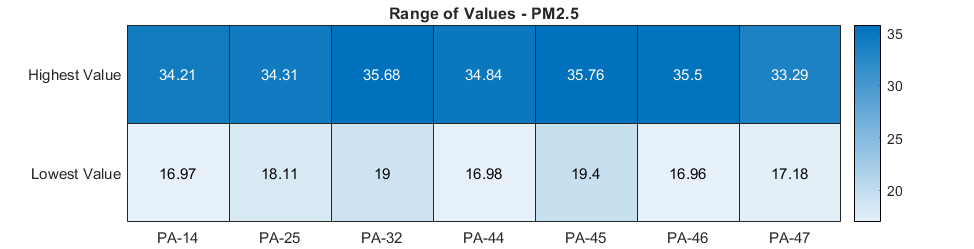


**S4 Fig.** Range of the PM2.5 values measured by the seven co-located sensors.

**
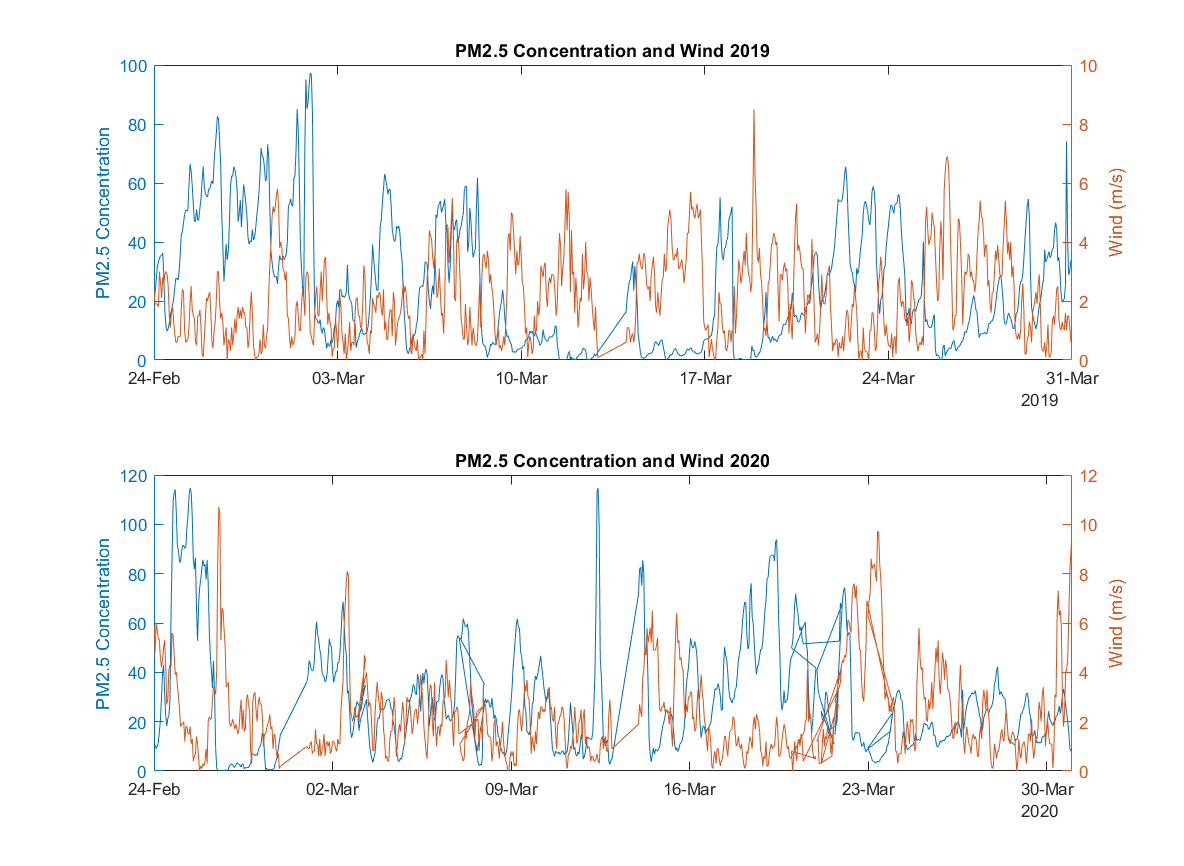
**

**S5 Fig. PM2.5 and Wind Variations in time in the considered periods in 2019 and 2020.** The upper plot shows 2019 data, the lower plot shows 2020 data.


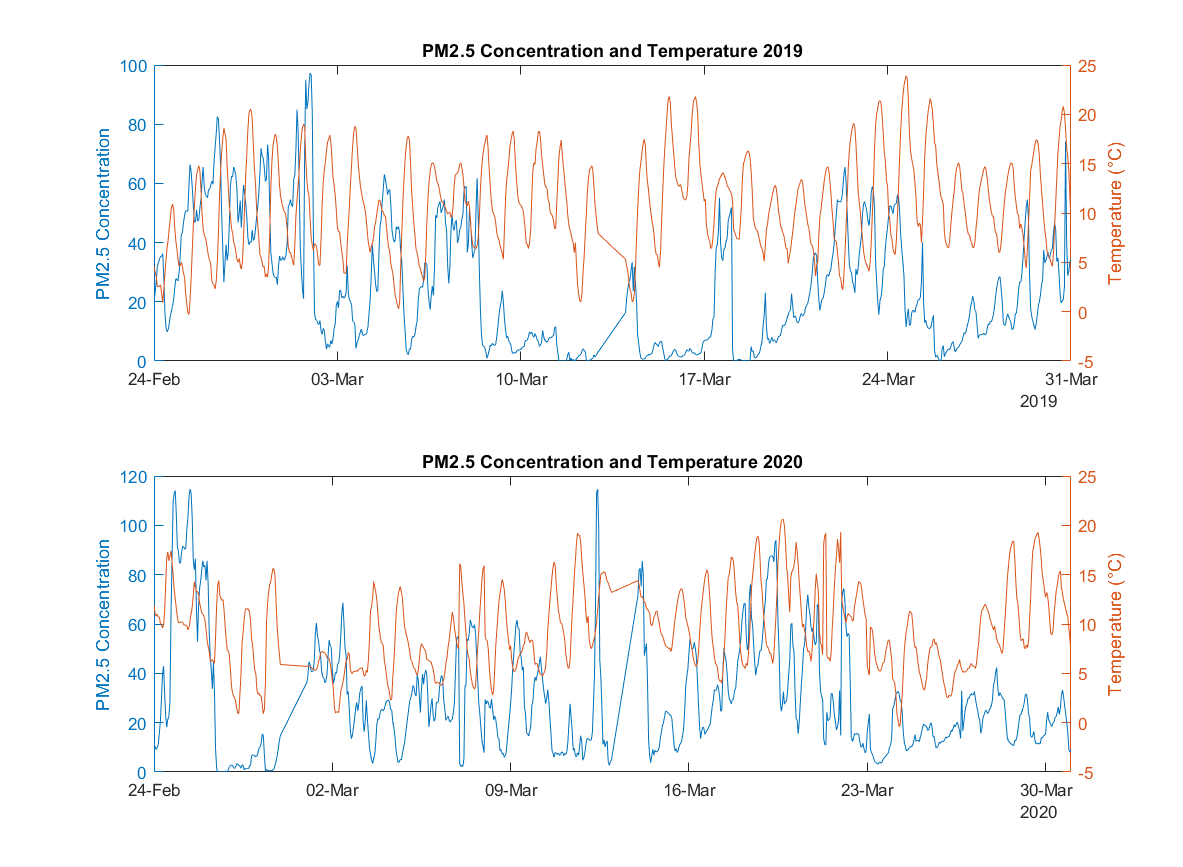


**S6 Fig.** PM2.5 concentration and temperature in the two considered periods in 2019 (upper plot) and 2020 (lower plot).

**
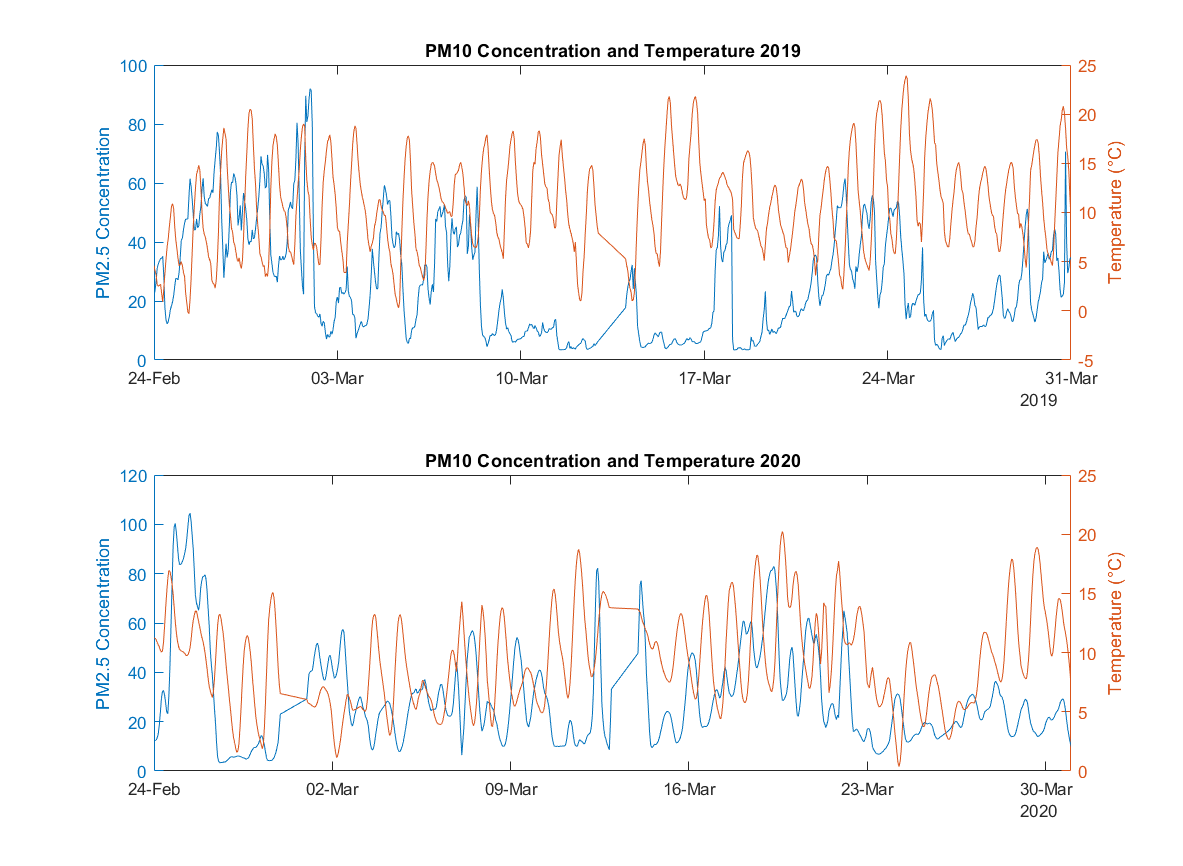
**

**S7 Fig. PM10 and Temperature Variations in time in the considered periods in 2019 and 2020.** The upper plot shows 2019 data, the lower plot shows 2020 data.

**
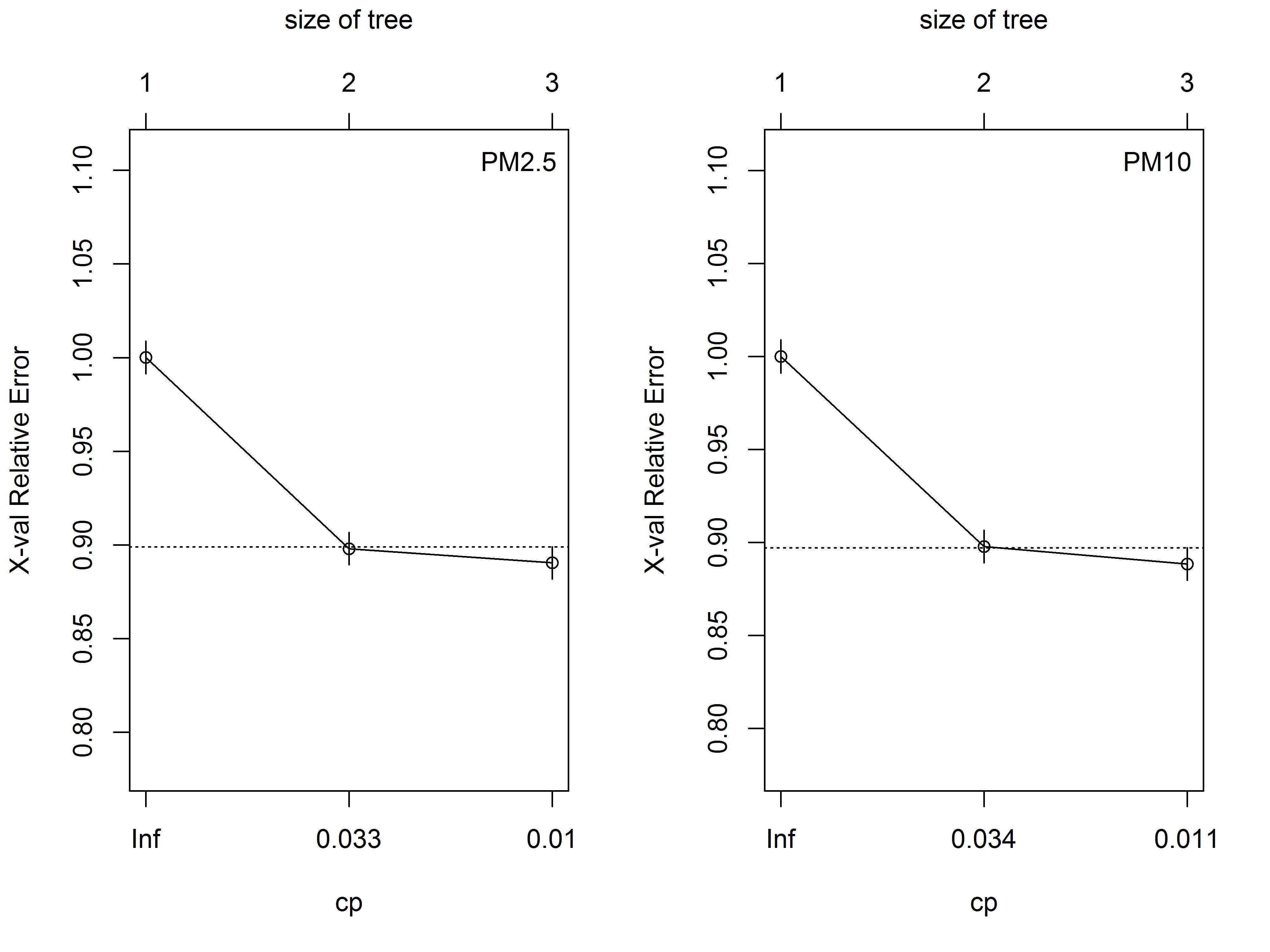
**

**S8 Fig. Visual representation of the cross-validation results for PM2.5 and PM10.** The x-axis represents the complexity parameter corresponding to different tree sizes while the y-axis represents the cross validation relative error.


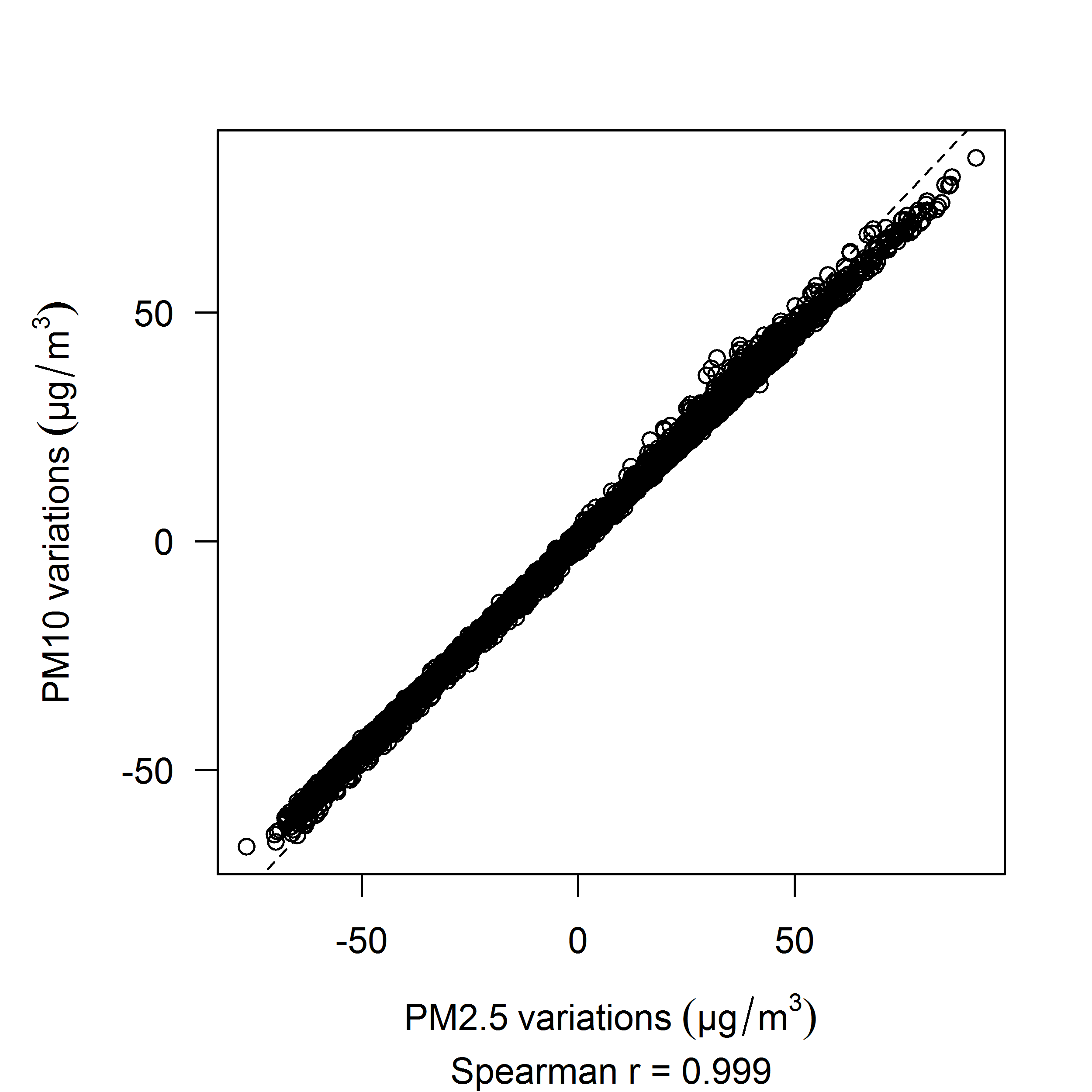


**S9 Fig.** Correlation between variations in terms PM2.5 and PM10 between year 2019 and 2020.


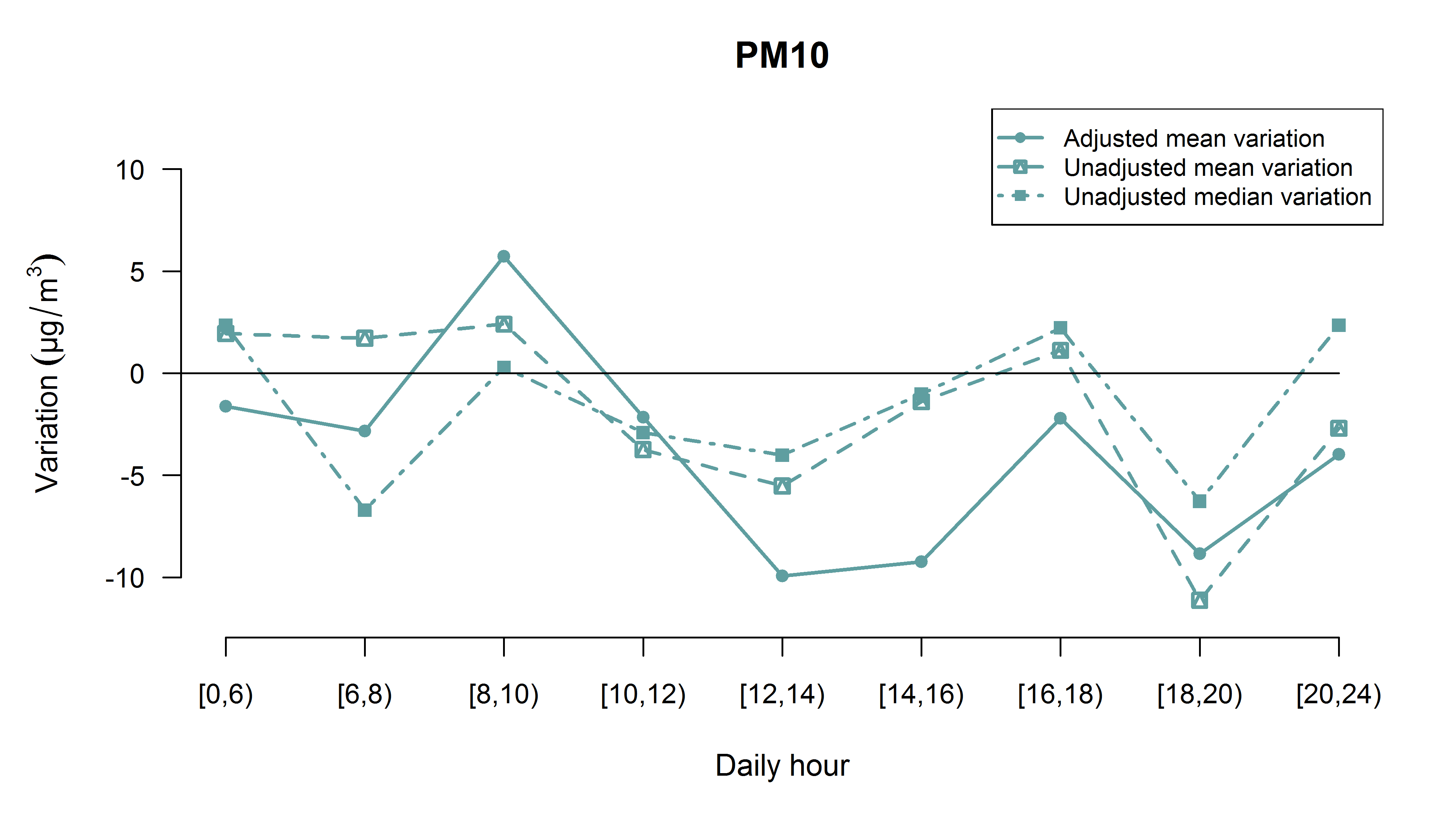


**S10 Fig.** PM10 variations by daily hour interval. Data are presented as adjusted mean variations, unadjusted mean variations and unadjusted median variations.

**
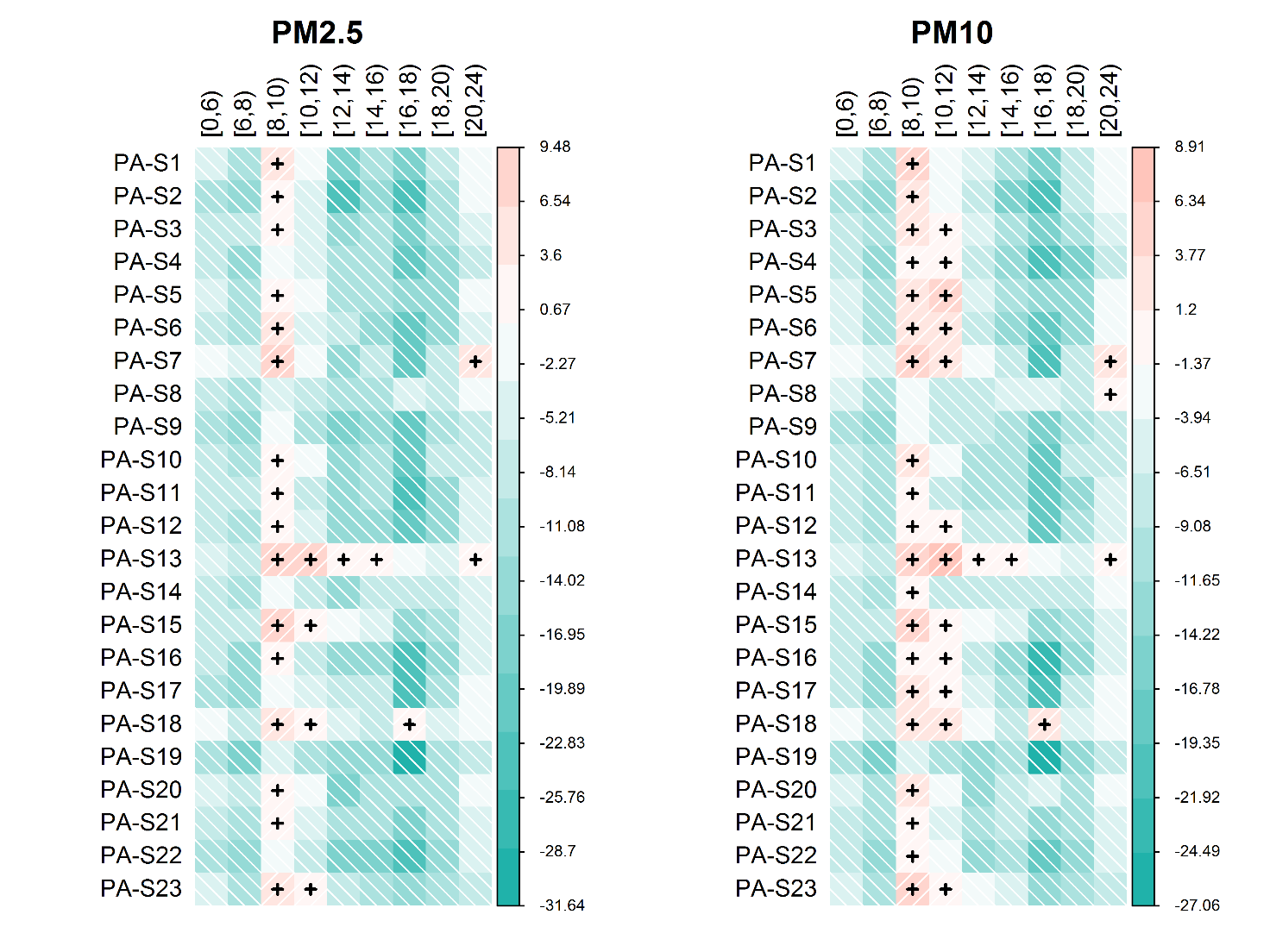
**

**S11 Fig. Heatmaps showing the median difference between observed and LMM - predicted PM2.5 and PM10 during 2020 by sensor and daily hour intervals**. Each heatmap row graphically represents the median absolute differences between observed and predicted pollutants measurements by daily hours intervals. Shades of green indicate negative absolute differences between observed and predicted pollutants concentration during 2020, shades of red indicate positive absolute differences between observed and predicted pollutants concentration during 2020 as showed by the colour code legend on the right side of each plot. The “+” symbol denotes a positive absolute differences between observed and predicted pollutants concentration.

**Supplementary Tables**

|  | **Hour** | | | | | | | | | |
| --- | --- | --- | --- | --- | --- | --- | --- | --- | --- | --- |
|  | **[0,6)** | **[6,8)** | **[8,10)** | **[10,12)** | **[12,14)** | **[14,16)** | **[16,18)** | **[18,20)** | **[20,24)** | **TOTAL** |
| ***All sensors*** | ***1872*** | ***776*** | ***549*** | ***295*** | ***340*** | ***295*** | ***228*** | ***297*** | ***800*** | ***5452*** |
| PA-S1 | 82 | 34 | 24 | 13 | 15 | 13 | 10 | 13 | 34 | 238 |
| PA-S2 | 78 | 32 | 24 | 11 | 14 | 13 | 9 | 12 | 34 | 227 |
| PA-S3 | 82 | 34 | 24 | 13 | 15 | 13 | 10 | 13 | 35 | 239 |
| PA-S4 | 82 | 34 | 24 | 13 | 15 | 13 | 10 | 13 | 35 | 239 |
| PA-S5 | 81 | 34 | 24 | 13 | 15 | 13 | 10 | 13 | 35 | 238 |
| PA-S6 | 82 | 34 | 24 | 13 | 15 | 13 | 10 | 13 | 35 | 239 |
| PA-S7 | 82 | 34 | 24 | 13 | 15 | 13 | 10 | 13 | 35 | 239 |
| PA-S8 | 82 | 34 | 24 | 13 | 15 | 13 | 10 | 13 | 35 | 239 |
| PA-S9 | 82 | 34 | 24 | 13 | 15 | 13 | 10 | 13 | 35 | 239 |
| PA-S10 | 82 | 34 | 24 | 13 | 15 | 13 | 10 | 13 | 35 | 239 |
| PA-S11 | 82 | 34 | 24 | 13 | 15 | 13 | 10 | 13 | 35 | 239 |
| PA-S12 | 82 | 34 | 24 | 13 | 15 | 13 | 10 | 13 | 35 | 239 |
| PA-S13 | 82 | 34 | 23 | 11 | 13 | 11 | 9 | 13 | 35 | 231 |
| PA-S14 | 82 | 34 | 24 | 13 | 15 | 13 | 10 | 13 | 35 | 239 |
| PA-S15 | 82 | 34 | 24 | 13 | 15 | 13 | 10 | 13 | 35 | 239 |
| PA-S16 | 82 | 34 | 24 | 13 | 15 | 13 | 10 | 13 | 35 | 239 |
| PA-S17 | 82 | 34 | 24 | 13 | 15 | 13 | 10 | 13 | 35 | 239 |
| PA-S18 | 73 | 30 | 22 | 13 | 13 | 12 | 10 | 12 | 33 | 218 |
| PA-S19 | 82 | 34 | 24 | 13 | 15 | 12 | 10 | 13 | 34 | 237 |
| PA-S20 | 82 | 34 | 24 | 13 | 15 | 13 | 10 | 13 | 35 | 239 |
| PA-S21 | 82 | 34 | 24 | 13 | 15 | 13 | 10 | 13 | 35 | 239 |
| PA-S22 | 82 | 34 | 24 | 13 | 15 | 13 | 10 | 13 | 35 | 239 |
| PA-S23 | 82 | 34 | 24 | 13 | 15 | 13 | 10 | 13 | 35 | 239 |

**S1 Table. Number of paired measurements available by sensor and hours**.

|  | **Adjusted mean PM2.5 variations by hour** | | | | | | | | |
| --- | --- | --- | --- | --- | --- | --- | --- | --- | --- |
| **Sensor** | **[0,6)** | **[6,8)** | **[8,10)** | **[10,12)** | **[12,14)** | **[14,16)** | **[16,18)** | **[18,20)** | **[20,24)** |
| ***All sensors*** | ***-1.77*** | ***-3.13*** | ***6.11*** | ***-2.52*** | ***-11.20*** | ***-10.54*** | ***-3.01*** | ***-10.37*** | ***-4.63*** |
| PA-S1 | 1.20 | -1.30 | 10.30 | -0.10 | -11.18 | -10.47 | -1.67 | -9.93 | -2.90 |
| PA-S2 | -2.33 | -3.55 | 6.69 | -2.08 | -12.76 | -10.82 | -6.48 | -8.69 | -4.68 |
| PA-S3 | -1.02 | -0.72 | 8.03 | -0.07 | -9.79 | -9.25 | -4.24 | -9.82 | -6.52 |
| PA-S4 | -2.70 | -4.92 | 4.46 | 0.60 | -8.59 | -10.19 | -3.77 | -12.56 | -6.24 |
| PA-S5 | 1.33 | -1.20 | 10.22 | 4.07 | -5.70 | -6.79 | -1.11 | -8.87 | -2.13 |
| PA-S6 | -0.82 | -3.04 | 7.00 | 2.50 | -7.44 | -9.19 | -2.58 | -10.86 | -4.33 |
| PA-S7 | 4.94 | 3.13 | 12.23 | 2.45 | -9.09 | -7.67 | -1.40 | -7.73 | 1.50 |
| PA-S8 | -2.54 | -4.03 | 4.37 | -4.32 | -12.88 | -10.62 | 1.42 | -6.96 | -2.09 |
| PA-S9 | -8.31 | -9.21 | -2.20 | -10.99 | -14.53 | -12.49 | -5.88 | -14.63 | -11.48 |
| PA-S10 | -2.37 | -3.23 | 4.12 | -5.63 | -12.09 | -10.93 | -2.54 | -10.53 | -6.39 |
| PA-S11 | -0.76 | -0.90 | 7.20 | -4.35 | -14.15 | -10.42 | -2.79 | -11.08 | -5.70 |
| PA-S12 | -2.45 | -5.45 | 5.46 | -2.66 | -11.66 | -12.53 | -5.78 | -12.76 | -5.31 |
| PA-S13 | -2.52 | -2.90 | 7.08 | 0.64 | -8.48 | -10.25 | -2.82 | -12.38 | -3.22 |
| PA-S14 | -3.35 | -3.24 | 6.64 | -2.13 | -9.86 | -9.56 | -0.02 | -7.59 | -1.35 |
| PA-S15 | -0.82 | 0.32 | 9.22 | -0.17 | -7.81 | -7.75 | -2.04 | -3.60 | -5.60 |
| PA-S16 | -1.82 | -3.35 | 6.29 | 1.22 | -8.39 | -11.82 | -7.65 | -10.88 | -6.86 |
| PA-S17 | -2.15 | -3.80 | 5.04 | -0.96 | -9.62 | -8.43 | -4.40 | -9.69 | -5.13 |
| PA-S18 | -1.35 | -2.43 | 5.96 | -6.90 | -12.22 | -12.28 | 1.60 | -10.96 | -2.72 |
| PA-S19 | -4.25 | -6.26 | 2.56 | -4.16 | -12.98 | -12.89 | -6.76 | -10.64 | -7.56 |
| PA-S20 | -1.35 | -2.95 | 5.79 | -5.62 | -14.68 | -11.84 | -0.51 | -12.54 | -1.27 |
| PA-S21 | -1.90 | -3.27 | 5.73 | -5.90 | -13.88 | -11.17 | -1.47 | -9.89 | -2.71 |
| PA-S22 | -4.57 | -6.55 | 1.60 | -9.32 | -16.98 | -14.89 | -5.97 | -14.01 | -7.78 |
| PA-S23 | -0.91 | -3.30 | 6.47 | -3.86 | -13.03 | -10.65 | -3.20 | -11.80 | -5.82 |

**S2 Table. Adjusted mean variations in terms of PM2.5 between 2019 and 2020 by sensor and daily hours**. Each PurpleAir (PA) ID corresponds to a different sensor. In green: reduction in terms of PM2.5 between 2019 and 2020; in red: increase in terms of PM2.5 between 2019 and 2020.

|  | **Adjusted mean PM10 variations by hour** | | | | | | | | |
| --- | --- | --- | --- | --- | --- | --- | --- | --- | --- |
| **Sensor** | **[0,6)** | **[6,8)** | **[8,10)** | **[10,12)** | **[12,14)** | **[14,16)** | **[16,18)** | **[18,20)** | **[20,24)** |
| ***All sensors*** | ***-1.62*** | ***-2.83*** | ***5.72*** | ***-2.16*** | ***-9.94*** | ***-9.24*** | ***-2.21*** | ***-8.84*** | ***-3.98*** |
| PA-S1 | 1.00 | -1.12 | 9.07 | -0.24 | -9.85 | -9.11 | -1.25 | -8.57 | -2.58 |
| PA-S2 | -2.28 | -3.16 | 6.08 | -2.13 | -11.93 | -9.78 | -5.59 | -7.70 | -4.18 |
| PA-S3 | -1.09 | -0.89 | 7.22 | 0.00 | -8.40 | -7.80 | -3.31 | -8.21 | -5.63 |
| PA-S4 | -2.82 | -4.75 | 4.31 | 0.89 | -7.49 | -9.16 | -2.79 | -11.01 | -5.62 |
| PA-S5 | 1.16 | -1.03 | 9.39 | 3.77 | -4.85 | -5.74 | -0.49 | -7.34 | -1.63 |
| PA-S6 | -0.51 | -2.48 | 6.61 | 2.55 | -6.38 | -7.85 | -1.69 | -9.23 | -3.53 |
| PA-S7 | 4.05 | 2.61 | 10.89 | 1.80 | -8.46 | -6.85 | -1.01 | -6.99 | 1.16 |
| PA-S8 | -2.60 | -4.00 | 3.68 | -4.24 | -12.05 | -9.79 | 1.73 | -5.83 | -2.03 |
| PA-S9 | -7.20 | -8.06 | -1.36 | -9.43 | -12.64 | -10.88 | -4.59 | -12.37 | -9.88 |
| PA-S10 | -1.86 | -2.73 | 4.21 | -4.40 | -10.23 | -9.28 | -1.78 | -8.75 | -5.13 |
| PA-S11 | -0.58 | -0.80 | 6.83 | -3.64 | -12.26 | -8.86 | -1.94 | -9.18 | -4.80 |
| PA-S12 | -2.06 | -4.68 | 5.40 | -2.11 | -10.34 | -11.15 | -4.64 | -11.02 | -4.46 |
| PA-S13 | -2.11 | -2.39 | 6.35 | 0.72 | -7.38 | -8.66 | -2.13 | -10.55 | -2.68 |
| PA-S14 | -3.25 | -3.20 | 6.25 | -1.83 | -8.84 | -8.49 | 0.58 | -6.25 | -1.13 |
| PA-S15 | -0.97 | -0.04 | 7.95 | -0.47 | -7.27 | -6.97 | -1.93 | -3.53 | -5.09 |
| PA-S16 | -1.57 | -2.92 | 5.90 | 1.21 | -7.44 | -10.49 | -6.54 | -9.39 | -6.05 |
| PA-S17 | -2.03 | -3.47 | 4.84 | -0.52 | -8.42 | -7.38 | -3.53 | -8.20 | -4.49 |
| PA-S18 | -1.39 | -2.18 | 5.25 | -6.14 | -11.09 | -10.94 | 1.75 | -9.57 | -2.63 |
| PA-S19 | -3.92 | -5.75 | 2.57 | -3.70 | -11.55 | -11.34 | -5.60 | -8.96 | -6.69 |
| PA-S20 | -1.22 | -2.65 | 5.44 | -4.96 | -12.78 | -10.10 | 0.22 | -10.75 | -0.89 |
| PA-S21 | -1.81 | -3.11 | 5.45 | -5.26 | -12.44 | -9.86 | -0.72 | -8.32 | -2.23 |
| PA-S22 | -3.36 | -5.32 | 2.97 | -7.82 | -15.18 | -13.04 | -4.09 | -11.49 | -6.08 |
| PA-S23 | -0.89 | -3.06 | 6.07 | -3.49 | -11.47 | -9.20 | -2.28 | -10.03 | -5.18 |

**S3 Table. Adjusted median variations in terms of PM10 between 2019 and 2020 by sensor and daily hours**. Each Purple Air (PA) ID corresponds to a different sensor. In green: reduction in terms of PM10 between 2019 and 2020; in red: increase in terms of PM10 between 2019 and 2020.

| Pollutant | Index | LMM |
| --- | --- | --- |
| PM2.5 | RMSE | 23.93 |
| PM2.5 | MAE | 18.94 |
| PM2.5 | *r* | 0.51 |
| PM10 | RMSE | 21.64 |
| PM10 | MAE | 17.15 |
| PM10 | *r* | 0.52 |

**S4 Table.** Root mean square error, mean absolute error and Pearson correlation coefficient r from the LMM regression method.

**References**

1. Venter ZS, Aunan K, Chowdhury S, Lelieveld J. COVID-19 lockdowns cause global air pollution declines. Proc Natl Acad Sci U S A. 2020;117(32):18984-18990. doi:10.1073/pnas.2006853117
