# Supplementary material for: Impact of COVID-19 lockdown on PM concentrations in an Italian Northern City: A year-by-year assessment
Source: PLoS One. 2022 Mar 28;17(3):e0263265. doi: 10.1371/journal.pone.0263265 (PMC8959169; doi:10.1371/journal.pone.0263265)
Supplement: Attachment — Submitted filename: Response to Reviewers.pdf [file pone.0263265.s017.pdf]

We would like to thank the Reviewer for the positive comments on our study and for raising interesting points regarding our research. The comments have been very beneficial to improve the quality of the paper and better conveying its message. It is true that there is considerable interest on the effect of the lockdown on air pollution, as the unique situation of last year caused an unprecedented opportunity to explore the real impact of traffic and some productive activities on air quality. The phenomenon is really complex, as it is influenced by a large number of external factors.

In this revision process, we addressed all the points of the review, adding new analyses and editing the manuscript where required. Concerning the list of comments, the detailed modifications are the following:

1) ***Detailed information on the sensor calibration: how each of the sensors are calibrated? Are all the sensors been co-located at one of the ARPA stations? Does the performance of the sensor change with time, and how this is controlled? How do the sensors compare with each other?***

   Purple Air sensors are already calibrated during their production before being made available for purchase, as stated in the official documentation. To increase the precision of their measurements, we applied a simple recalibration method, explained in lines 176-204 of the manuscript, where we used the data coming from the official ARPA sensors as reference, since those sensors are calibrated with reliable procedures according to the European standards (see point 3). A parallel study that was performed at the University of Pavia showed that co-located Purple Air sensors measure PMs with high correlation, and therefore that the sensors are expected to provide consistent results between each other. For this reason we used only one sensor colocated with the ARPA one to estimate the correction to be made, and then applied it to all the other sensors. Considering that Pavia is a small city with no significant climatic differences within different zones, we assume that a possible change in performance over time does not affect some sensors more than others. The calibration process is repeated every year. We added a more detailed explanation in lines 194-203 and reported the results of the co-location studies in supplementary figures 1-4.

2) Time series figures or raw data for each sensor should be provided, perhaps as a supplementary table or dataset. It may be easier to separate the sensor location into urban background, traffic and rural background (if any) and present the average data. If so, the original raw data should also be provided.

The raw sensor data will be provided on a GitHub folder, both for the Purple Air sensors and the ARPA ones. Pavia is a small city and does not have significant changes in urban landscape, as there are not extremely urbanized areas and industrial sites inside the city borders. We also did not install sensors in rural areas (which are outside of the municipality borders). Nevertheless, the central area of the city is mostly closed to traffic, so we divided the sensors according to their locations into two categories: center and traffic areas. These categories have been added to the raw data. Most sensors belong to the traffic areas category and significant differences in the data from the two categories were not observed.

3) What data are available from the ARPA stations and how they are measured? It mentioned ARPA sensors – what are they?

ARPA Lombardia (Environmental Protection Regional Agency of the region of Lombardy) is a public agency that has the aim of measuring and treating environmental data of the region the

city of Pavia is located in. Through numerous sensors scattered throughout the region, ARPA collects a large quantity of data about air pollution, meteorology, agriculture, sole status etc.

In Pavia, there are two official ARPA air quality monitoring stations, they are high-quality fixed stations that measure several pollutants (NOX, SO2, CO, O3, PM10 and PM2.5) at regular time intervals. These sensors are calibrated continuously using other commercially available instruments that can be used as reference according to Italian or European laws (https://www.arpalombardia.it/Pages/Aria/Rete-di-rilevamento/Qualit%C3%A0-dei-dati/Taratura-degli-strument.aspx?firstlevel=Rete%20di%20rilevamento) and therefore can be considered very reliable. Weather parameters are collected as well, and data is freely available upon request on a dedicated portal.

We added this information in the manuscript, lines 132-149.

4) Figure 1 – short-term air pollution levels are highly dependent on meteorology. Is it really meaningful to compare the concentration of PM10 levels in late Feb to March in 2020 with that in 2019? What is interesting is that there are large differences for the two stations (2020 vs 2019). It would be important to explain why this is the case. The quality of the figure is not so good but this can be rectified at a later stage. You may want to put the two figures into one.

It is true that pollution levels are highly dependent on meteorology, and unfortunately spring months like March are the most meteorologically unstable months in which wind, humidity, pressure etc. can vary very quickly and influence air pollution's measurements. The differences between 2019 and 2020 are due to this variability, as 2019 had a warmer spring mostly dominated by high pressure systems and higher temperatures, whereas 2020 had a generally lower temperature and higher instability. The aim of this image is to show the effects of this variability and prove that meteorology can affect the measurements in a significant way. Since the lockdown was established in this period, comparing data in this unstable month was not a choice, but our analyses explicitly consider the effects of meteorological variables to avoid confounding factors (see also point 6). Comments about this have been added in lines 268-271.

5) Line 220 – this should be in the methodology?

We thank the Reviewer for the suggestion and moved it to the Methods section (lines 203-208).

6) Line 226 – this section explored the effect of meteorology on air pollution levels. There are a number of figures but the key messages could be made clearer. Why only wind speed and temperature? Other meteorological conditions such as wind direction, relative humidity, cloud cover, precipitation, and back trajectories all could contribute to the variations in air quality. The key point of this section is to establish the regression models so that you can predict the concentrations under same meteorological conditions whether that is in 2020 or 2019. Although this regression model is not as good as machine learning based techniques, such as based on random forest algorithms (you should be able to find relevant papers), it has been used by Zenter et al. (https://www.pnas.org/content/pnas/117/32/18984.full.pdf). Only by doing this, you can compare the data in 2019 with that in 2020.

We thank the Reviewer for highlighting these aspects: we updated the analyses by including additional potential confounding factors in the models and tested an additional methodology to estimate the impact of the lock-down on pollutants concentration.

The set of confounders considered in the analyses now include: wind (m/s), temperature (°C), humidity (%), precipitations (mm) and solar radiation (W/m^2). The correlation between the updated set of variables and PM2.5 and PM10 concentrations has been extensively explored as described in the Results section with title "*Effect of potential confounders on pollutants concentration*". In particular, an updated approach to data filtering has been applied as reported in the following. Based on the scatterplots in Fig 6 we observed a non-linear relationship between wind speed and pollutants concentration (Fig 4A and Fig 4F). With the aim to identify informative wind speed cut off values able to distinguish subpopulations of measurements, univariate regression trees were fitted including wind speed as predictor while PM2.5 and PM10 as dependent variables in turn. By imposing a single split to the regression tree algorithm, a wind speed of 2.15 m/s was identified as the most informative threshold to stratify both PM2.5 and PM10 levels. Further, plots in Fig 4C and Fig 4H evidenced that pollutants concentration did not vary with respect to humidity when humidity values were below ~ 20%, highlighting a potential bias in terms of measurements accuracy when the confounder value is below this threshold. It was then decided to focus on measures performed when the wind speed was below 2.15 m/s and humidity > 20% to avoid confounding effects.
As an additional approach to estimate the effect of the lock-down on pollutants concentration, a methodology based on the method described in Venter et al. (https://www.pnas.org/content/pnas/117/32/18984.full.pdf) has been applied as described in the Supplementary Data section. In details, the implemented methodology consists of the following steps:

a) Train linear mixed model regression models (LMM) using data from 2019 at a sensor level, using PM2.5 and PM10 levels in turn as dependent variable and the following predictors as independent variables: working day, wind, temperature, humidity, precipitations, solar radiation, day/month, daily hour categories. The day/month information is used as random effect grouping variable, while the remaining ones as fixed terms.
b) Apply the LMM model trained on data from 2019 to forecast PM2.5 and PM10 concentrations during 2020.
c) Using the predicted pollutants concentration as benchmark, compare the predicted and the observed PM2.5 and PM10 values to estimate the absolute change (observed – predicted) in terms of pollutants concentration. Positive changes indicate an increase in terms of pollutants concentration compared to the expected values, negative changes a decrease in terms of pollutants concentration compared to the expected values.

The Pearson correlation coefficient *r* between observed and predicted PM2.5 and PM10 concentrations during 2020 was + 0.51 for PM2.5 and + 0.52 for PM10 (Supplementary Table 4). The median value of the absolute differences between observed and predicted pollutants concentration by sensor and daily hour showed trends concordant with what estimated by the method used in our manuscript (Supplementary Figure 8 vs. Figure 9).

7) Table 2: do not understand this. For example, what does intercept mean?

Table 2 reports the regression coefficients, 95% confidence intervals and p-values corresponding to the set of variables included in the multivariate linear mixed effects model regression fitted to estimate the mean variation in terms of PM2.5 and PM10 between 2019 and 2020 accounting for confounders. The regression coefficient corresponding to the "year (2020)" term quantifies the average variation in terms of PM2.5 and PM10 pollutants concentration between 2019 and 2020 accounting for potential confounding effect of the other variables included in the model and reported in the table. For sake of clarity the coefficients and significance corresponding to the intercept term have been now removed from Table 2. These aspects have been described more in the detail in Table 2 legend and in the corresponding text section.

8) Section starting line 308: this is interesting. Consider putting data in Table 3 and 4 in the SI and present the data in figures. It would be much easier to see the trend.

We made the proposed change, and we thank the Reviewer for the suggestion. Table 3 and Table 4 have been moved to Supplementary Data section (Supplementary Table 2 and Supplementary Table 3) and replaced by the two Heatmaps in Figure 9 graphically resuming the same information.

9) Is there longer-term changes in PM levels in the study region? For example, are the PM levels reducing in the previous years? If so, then the PM levels will be lower in 2020 whether or not there is a lockdown. You may need to take this "trend" into account as well. This is called "detrend".

There is some evidence of a reduction trend in all the area in the last years (https://www.infodata.ilsole24ore.com/2021/01/31/qualita-dellaria-italia-migliorata-negli-ultimi-cinque-anni-cosa-misura-snpa/?refresh_ce=1), although with notable fluctuations. Looking at the data gathered by the Italian National Environmental Protection System, it can be noticed that the reduction trend appears less evident after 2018, with even a little increase, probably not significant, in the PM10 concentrations in 2020. The article itself states that the meteorological variability could have played an important role in the measurements' variations, as in 2019 and 2020 temperatures were generally higher and precipitations lower than the previous years. Therefore, we do not assume that the general trend of the last years could influence the difference in PM2.5 and PM10 concentrations between 2019 and 2020. We added a few lines about this (lines 210-224) with the proper citations.

10) Discussions – this seems very general. It would be important to explain your results, e.g., why there is no obvious change in PM levels? We know the emission reduced in 2020 as a result of the lockdown. Is it due to the sensor uncertainty, or meteorology difference in 2019 and 2020, or is it due to the negligible contribution to PM levels from road traffic? Or is it due to changing chemistry? Do you see variations in different types of sites, for example, do you see changes in PM levels at roadside sites but not at the urban background sites? The changing diurnal patterns are really interesting and suggest different emission sources. Are there any other data, such as NO2, CO, BC data from the monitoring stations that can potentially help to interpret the results better? The second part of the discussion should focus on the implications of the results for air pollution control in the study region.

The discussion has been extended according to the new results and the Reviewer's comments, especially from line 480.
