# Supplementary material for: Impact of COVID-19 lockdown on PM concentrations in an Italian Northern City: A year-by-year assessment
Source: PLoS One. 2022 Mar 28;17(3):e0263265. doi: 10.1371/journal.pone.0263265 (PMC8959169; doi:10.1371/journal.pone.0263265)
Supplement: Attachment — Submitted filename: PONE-D-21-17702_R1.pdf [file pone.0263265.s018.pdf]

PLOS ONE

Impact of COVID-19 Lockdown on PM Concentrations in an Italian Northern City: a year-by-year Assessment

--Manuscript Draft--

**Manuscript Number:**	PONE-D-21-17702R1
**Article Type:**	Research Article
**Full Title:**	Impact of COVID-19 Lockdown on PM Concentrations in an Italian Northern City: a year-by-year Assessment
**Short Title:**	Assessment of the Impact of COVID-19 Lockdown on PM Concentrations in an Italian City
**Corresponding Author:**	Daniele Pala, Ph.D.<br>University of Pavia: Universita degli Studi di Pavia<br>Pavia, ITALY
**Keywords:**	Lockdown; Air Pollution; air quality; Public health; sensors; PM10; PM2.5; Mixed-effects Models
**Abstract:**	In the last century, the increase in traffic, human activities and industrial production have led to a diffuse presence of air pollution, which causes an increase of risk of several health conditions such as respiratory diseases. In Europe, air pollution is a serious concern that affects several areas, one of the worst ones being northern Italy, and in particular the Po Valley, an area characterized by low air quality due to a combination of high population density, industrial activity, geographical factors and weather conditions. Public health authorities and local administrations are aware of this problem, and periodically intervene with temporary traffic limitations and other regulations, often insufficient to solve the problem. In February 2020, this area was the first in Europe to be severely hit by the SARS-CoV-2 virus causing the COVID-19 disease, to which the Italian government reacted with the establishment of a drastic lockdown. This situation created the condition to study how significant is the impact of car traffic and industrial activity on the pollution in the area, as these factors were strongly reduced during the lockdown. Differently from other areas in the world, a drastic decrease in pollution measured in terms of particulate matter (PM) was not observed in the Po Valley during the lockdown, suggesting that several external factors can play a role in determining the severity of pollution. In this study, we report the case study of the city of Pavia, where data coming from 23 air quality sensors were analyzed to compare the levels measured during the lockdown with the ones coming from the same period in 2019. Our results show that, on a global scale, there was a statistically significant reduction in terms of PM levels taking into account meteorological variables that can influence pollution such as wind, temperature, humidity, rain and solar radiation. Differences can be noticed analyzing daily pollution trends too, as – compared to 2019 – during 2020 pollution was higher in the morning and lower in the remaining hours.
**Order of Authors:**	Daniele Pala, Ph.D.<br>Vittorio Casella<br>Cristiana Larizza<br>Alberto Malovini<br>Riccardo Bellazzi
**Opposed Reviewers:**	
**Response to Reviewers:**	We would like to thank the Reviewer for the positive comments on our study and for raising interesting points regarding our research. The comments have been very beneficial to improve the quality of the paper and better conveying its message. It is true that there is considerable interest on the effect of the lockdown on air pollution, as the unique situation of last year caused an unprecedented opportunity to explore the real impact of traffic and some productive activities on air quality. The phenomenon is really complex, as it is influenced by a large number of external factors.

Powered by Editorial Manager® and ProduXion Manager® from Aries Systems Corporation

In this revision process, we addressed all the points of the review, adding new analyses and editing the manuscript where required. Concerning the list of comments, the detailed modifications are the following:

1)Detailed information on the sensor calibration: how each of the sensors are calibrated? Are all the sensors been co-located at one of the ARPA stations? Does the performance of the sensor change with time, and how this is controlled? How do the sensors compare with each other?
Purple Air sensors are already calibrated during their production before being made available for purchase, as stated in the official documentation. To increase the precision of their measurements, we applied a simple recalibration method, explained in lines 176-204 of the manuscript, where we used the data coming from the official ARPA sensors as reference, since those sensors are calibrated with reliable procedures according to the European standards (see point 3). A parallel study that was performed at the University of Pavia showed that co-located Purple Air sensors measure PMs with high correlation, and therefore that the sensors are expected to provide consistent results between each other. For this reason we used only one sensor colocated with the ARPA one to estimate the correction to be made, and then applied it to all the other sensors. Considering that Pavia is a small city with no significant climatic differences within different zones, we assume that a possible change in performance over time does not affect some sensors more than others. The calibration process is repeated every year. We added a more detailed explanation in lines 194-203 and reported the results of the co-location studies in supplementary figures 1-4.

2) Time series figures or raw data for each sensor should be provided, perhaps as a supplementary table or dataset. It may be easier to separate the sensor location into urban background, traffic and rural background (if any) and present the average data. If so, the original raw data should also be provided.
The raw sensor data will be provided on a GitHub folder, both for the Purple Air sensors and the ARPA ones. Pavia is a small city and does not have significant changes in urban landscape, as there are not extremely urbanized areas and industrial sites inside the city borders. We also did not install sensors in rural areas (which are outside of the municipality borders). Nevertheless, the central area of the city is mostly closed to traffic, so we divided the sensors according to their locations into two categories: center and traffic areas. These categories have been added to the raw data. Most sensors belong to the traffic areas category and significant differences in the data from the two categories were not observed.

3) What data are available from the ARPA stations and how they are measured? It mentioned ARPA sensors – what are they?
ARPA Lombardia (Environmental Protection Regional Agency of the region of Lombardy) is a public agency that has the aim of measuring and treating environmental data of the region the city of Pavia is located in. Through numerous sensors scattered throughout the region, ARPA collects a large quantity of data about air pollution, meteorology, agriculture, sole status etc.
In Pavia, there are two official ARPA air quality monitoring stations, they are high-quality fixed stations that measure several pollutants (NOX, SO2, CO, O3, PM10 and PM2.5) at regular time intervals. These sensors are calibrated continuously using other commercially available instruments that can be used as reference according to Italian or European laws (https://www.arpalombardia.it/Pages/Aria/Rete-di-rilevamento/Qualit%C3%A0-dei-dati/Taratura-degli-strument.aspx?firstlevel=Rete%20di%20rilevamento) and therefore can be considered very reliable. Weather parameters are collected as well, and data is freely available upon request on a dedicated portal.
We added this information in the manuscript, lines 132-149.

4) Figure 1 – short-term air pollution levels are highly dependent on meteorology. Is it really meaningful to compare the concentration of PM10 levels in late Feb to March in 2020 with that in 2019? What is interesting is that there are large differences for the two stations (2020 vs 2019). It would be important to explain why this is the case. The quality of the figure is not so good but this can be rectified at a later stage. You may want to put the two figures into one.
It is true that pollution levels are highly dependent on meteorology, and unfortunately

Powered by Editorial Manager® and ProduXion Manager® from Aries Systems Corporation

spring months like March are the most meteorologically unstable months in which wind, humidity, pressure etc. can vary very quickly and influence air pollution's measurements. The differences between 2019 and 2020 are due to this variability, as 2019 had a warmer spring mostly dominated by high pressure systems and higher temperatures, whereas 2020 had a generally lower temperature and higher instability. The aim of this image is to show the effects of this variability and prove that meteorology can affect the measurements in a significant way. Since the lockdown was established in this period, comparing data in this unstable month was not a choice, but our analyses explicitly consider the effects of meteorological variables to avoid confounding factors (see also point 6). Comments about this have been added in lines 268-271.

5) Line 220 – this should be in the methodology?
We thank the Reviewer for the suggestion and moved it to the Methods section (lines 203-208).

6) Line 226 – this section explored the effect of meteorology on air pollution levels. There are a number of figures but the key messages could be made clearer. Why only wind speed and temperature? Other meteorological conditions such as wind direction, relative humidity, cloud cover, precipitation, and back trajectories all could contribute to the variations in air quality. The key point of this section is to establish the regression models so that you can predict the concentrations under same meteorological conditions whether that is in 2020 or 2019. Although this regression model is not as good as machine learning based techniques, such as based on random forest algorithms (you should be able to find relevant papers), it has been used by Zenter et al. (https://www.pnas.org/content/pnas/117/32/18984.full.pdf). Only by doing this, you can compare the data in 2019 with that in 2020.
We thank the Reviewer for highlighting these aspects: we updated the analyses by including additional potential confounding factors in the models and tested an additional methodology to estimate the impact of the lock-down on pollutants concentration.
The set of confounders considered in the analyses now include: wind (m/s), temperature (°C), humidity (%), precipitations (mm) and solar radiation (W/m2). The correlation between the updated set of variables and PM2.5 and PM10 concentrations has been extensively explored as described in the Results section with title "Effect of potential confounders on pollutants concentration". In particular, an updated approach to data filtering has been applied as reported in the following. Based on the scatterplots in Fig 6 we observed a non-linear relationship between wind speed and pollutants concentration (Fig 4A and Fig 4F). With the aim to identify informative wind speed cut off values able to distinguish subpopulations of measurements, univariate regression trees were fitted including wind speed as predictor while PM2.5 and PM10 as dependent variables in turn. By imposing a single split to the regression tree algorithm, a wind speed of 2.15 m/s was identified as the most informative threshold to stratify both PM2.5 and PM10 levels. Further, plots in Fig 4C and Fig 4H evidenced that pollutants concentration did not vary with respect to humidity when humidity values were below ~ 20%, highlighting a potential bias in terms of measurements accuracy when the confounder value is below this threshold. It was then decided to focus on measures performed when the wind speed was below 2.15 m/s and humidity > 20% to avoid confounding effects.
As an additional approach to estimate the effect of the lock-down on pollutants concentration, a methodology based on the method described in Venter et al. (https://www.pnas.org/content/pnas/117/32/18984.full.pdf) has been applied as described in the Supplementary Data section. In details, the implemented methodology consists of the following steps:
a)Train linear mixed model regression models (LMM) using data from 2019 at a sensor level, using PM2.5 and PM10 levels in turn as dependent variable and the following predictors as independent variables: working day, wind, temperature, humidity, precipitations, solar radiation, day/month, daily hour categories. The day/month information is used as random effect grouping variable, while the remaining ones as fixed terms.
b)Apply the LMM model trained on data from 2019 to forecast PM2.5 and PM10 concentrations during 2020.
c)Using the predicted pollutants concentration as benchmark, compare the predicted and the observed PM2.5 and PM10 values to estimate the absolute change (observed

– predicted) in terms of pollutants concentration. Positive changes indicate an increase in terms of pollutants concentration compared to the expected values, negative changes a decrease in terms of pollutants concentration compared to the expected values.
The Pearson correlation coefficient r between observed and predicted PM2.5 and PM10 concentrations during 2020 was + 0.51 for PM2.5 and + 0.52 for PM10 (Supplementary Table 4). The median value of the absolute differences between observed and predicted pollutants concentration by sensor and daily hour showed trends concordant with what estimated by the method used in our manuscript (Supplementary Figure 8 vs. Figure 9).

7) Table 2: do not understand this. For example, what does intercept mean?
Table 2 reports the regression coefficients, 95% confidence intervals and p-values corresponding to the set of variables included in the multivariate linear mixed effects model regression fitted to estimate the mean variation in terms of PM2.5 and PM10 between 2019 and 2020 accounting for confounders. The regression coefficient corresponding to the "year (2020)" term quantifies the average variation in terms of PM2.5 and PM10 pollutants concentration between 2019 and 2020 accounting for potential confounding effect of the other variables included in the model and reported in the table. For sake of clarity the coefficients and significance corresponding to the intercept term have been now removed from Table 2. These aspects have been described more in the detail in Table 2 legend and in the corresponding text section.

8) Section starting line 308: this is interesting. Consider putting data in Table 3 and 4 in the SI and present the data in figures. It would be much easier to see the trend.
We made the proposed change, and we thank the Reviewer for the suggestion. Table 3 and Table 4 have been moved to Supplementary Data section (Supplementary Table 2 and Supplementary Table 3) and replaced by the two Heatmaps in Figure 9 graphically resuming the same information.

9) Is there longer-term changes in PM levels in the study region? For example, are the PM levels reducing in the previous years? If so, then the PM levels will be lower in 2020 whether or not there is a lockdown. You may need to take this "trend" into account as well. This is called "detrend".
There is some evidence of a reduction trend in all the area in the last years (https://www.infodata.ilsole24ore.com/2021/01/31/qualita-dellaria-italia-migliorata-negli-ultimi-cinque-anni-cosa-misura-snpa/?refresh_ce=1), although with notable fluctuations. Looking at the data gathered by the Italian National Environmental Protection System, it can be noticed that the reduction trend appears less evident after 2018, with even a little increase, probably not sig[illegible]ant, in the PM10 concentrations in 2020. The article itself states that the meteorolog[illegible]variability could have played an important role in the measurements' variations, as in 2019 and 2020 temperatures were generally higher and precipitations lower than the previous years. Therefore, we do not assume that the general trend of the last years could influence the difference in PM2.5 and PM10 concentrations between 2019 and 2020. We added a few lines about this (lines 210-224) with the proper citations.

10) Discussions – this seems very general. It would be important to explain your results, e.g., why there is no obvious change in PM levels? We know the emission reduced in 2020 as a result of the lockdown. Is it due to the sensor uncertainty, or meteorology difference in 2019 and 2020, or is it due to the negligible contribution to PM levels from road traffic? Or is it due to changing chemistry? Do you see variations in different types of sites, for example, do you see changes in PM levels at roadside sites but not at the urban background sites? The changing diurnal patterns are really interesting and suggest different emission sources. Are there any other data, such as NO2, CO, BC data from the monitoring stations that can potentially help to interpret the results better? The second part of the discussion should focus on the implications of the results for air pollution control in the study region.
The discussion has been extended according to the new results and the Reviewer's comments, especially from line 480.

**Additional Information:**	
**Question**	**Response**

Powered by Editorial Manager® and ProduXion Manager® from Aries Systems Corporation

**Financial Disclosure**<br><br>Enter a financial disclosure statement that describes the sources of funding for the work included in this submission. Review the submission guidelines for detailed requirements. View published research articles from *PLOS ONE* for specific examples.<br><br>This statement is required for submission and **will appear in the published article** if the submission is accepted. Please make sure it is accurate.<br><br>**Unfunded studies**<br>Enter: *The author(s) received no specific funding for this work.*<br><br>**Funded studies**<br>Enter a statement with the following details:<br>• Initials of the authors who received each award<br>• Grant numbers awarded to each author<br>• The full name of each funder<br>• URL of each funder website<br>• Did the sponsors or funders play any role in the study design, data collection and analysis, decision to publish, or preparation of the manuscript?<br>• **NO** - Include this sentence at the end of your statement: *The funders had no role in study design, data collection and analysis, decision to publish, or preparation of the manuscript.*<br>• **YES** - Specify the role(s) played.<br><br>* typeset	The work has been funded by the European Commission with Grant Agreements GA-727816 and GA-101016233. All authors received funding. The funders had no role in study design, data collection and analysis, decision to publish, or preparation of the manuscript.
**Competing Interests**<br><br>Use the instructions below to enter a competing interest statement for this submission. On behalf of all authors, disclose any competing interests that could be perceived to bias this work—acknowledging all financial support and any other relevant financial or non-financial competing interests.<br><br>This statement is required for submission	The authors have declared that no competing interests exist.

Powered by Editorial Manager® and ProduXion Manager® from Aries Systems Corporation

and **will appear in the published article** if the submission is accepted. Please make sure it is accurate and that any funding sources listed in your Funding Information later in the submission form are also declared in your Financial Disclosure statement.<br><br>View published research articles from *PLOS ONE* for specific examples.<br><br>**NO authors have competing interests**<br><br>Enter: *The authors have declared that no competing interests exist.*<br><br>**Authors with competing interests**<br><br>Enter competing interest details beginning with this statement:<br><br>*I have read the journal's policy and the authors of this manuscript have the following competing interests: [insert competing interests here]*<br><br>* typeset	
**Ethics Statement**<br><br>Enter an ethics statement for this submission. This statement is required if the study involved:<br><br>• Human participants<br>• Human specimens or tissue<br>• Vertebrate animals or cephalopods<br>• Vertebrate embryos or tissues<br>• Field research<br><br>Write "N/A" if the submission does not require an ethics statement.<br><br>General guidance is provided below. Consult the submission guidelines for detailed instructions. **Make sure that all information entered here is included in the Methods section of the manuscript.**	N/A

Powered by Editorial Manager® and ProduXion Manager® from Aries Systems Corporation

**Format for specific study types**<br><br>**Human Subject Research (involving human participants and/or tissue)**<br>• Give the name of the institutional review board or ethics committee that approved the study<br>• Include the approval number and/or a statement indicating approval of this research<br>• Indicate the form of consent obtained (written/oral) or the reason that consent was not obtained (e.g. the data were analyzed anonymously)<br><br>**Animal Research (involving vertebrate animals, embryos or tissues)**<br>• Provide the name of the Institutional Animal Care and Use Committee (IACUC) or other relevant ethics board that reviewed the study protocol, and indicate whether they approved this research or granted a formal waiver of ethical approval<br>• Include an approval number if one was obtained<br>• If the study involved *non-human primates*, add *additional details* about animal welfare and steps taken to ameliorate suffering<br>• If anesthesia, euthanasia, or any kind of animal sacrifice is part of the study, include briefly which substances and/or methods were applied<br><br>**Field Research**<br><br>Include the following details if this study involves the collection of plant, animal, or other materials from a natural setting:<br>• Field permit number<br>• Name of the institution or relevant body that granted permission	
**Data Availability**<br><br>Authors are required to make all data underlying the findings described fully available, without restriction, and from the time of publication. PLOS allows rare exceptions to address legal and ethical concerns. See the PLOS Data Policy and FAQ for detailed information.	No - some restrictions will apply

Powered by Editorial Manager® and ProduXion Manager® from Aries Systems Corporation

A Data Availability Statement describing where the data can be found is required at submission. Your answers to this question constitute the Data Availability Statement and **will be published in the article**, if accepted.<br><br>**Important:** Stating 'data available on request from the author' is not sufficient. If your data are only available upon request, select 'No' for the first question and explain your exceptional situation in the text box.<br><br>Do the authors confirm that all data underlying the findings described in their manuscript are fully available without restriction?	
**Describe where the data may be found in full sentences. If you are copying our sample text, replace any instances of XXX with the appropriate details.**<br><br>• If the data are **held or will be held in a public repository**, include URLs, accession numbers or DOIs. If this information will only be available after acceptance, indicate this by ticking the box below. For example: *All XXX files are available from the XXX database (accession number(s) XXX, XXX.).*<br>• If the data are all contained **within the manuscript and/or Supporting Information files**, enter the following: *All relevant data are within the manuscript and its Supporting Information files.*<br>• If neither of these applies but you are able to provide **details of access elsewhere**, with or without limitations, please do so. For example:<br><br>*Data cannot be shared publicly because of [XXX]. Data are available from the XXX Institutional Data Access / Ethics Committee (contact via XXX) for researchers who meet the criteria for access to confidential data.*<br><br>*The data underlying the results presented in the study are available from (include the name of the third party*	Data are currently held by the University of Pavia, but URLs and/or accession numbers and/or DOIs will be made available after acceptance. private access to reviewers and editors can be provided if requested.

Powered by Editorial Manager® and ProduXion Manager® from Aries Systems Corporation

and contact information or URL).<br>• This text is appropriate if the data are owned by a third party and authors do not have permission to share the data.<br><br>* typeset	
Additional data availability information:	Tick here if the URLs/accession numbers/DOIs will be available only after acceptance of the manuscript for publication so that we can ensure their inclusion before publication.

Powered by Editorial Manager® and ProduXion Manager® from Aries Systems Corporation

Dear Editor-in-Chief,

we are glad to send you our new manuscript entitled "Impact of COVID-19 Lockdown on PM Concentrations in an Italian Northern City: a year-by-year Assessment" to be considered for publication as a Research Article in PLOS ONE.

The strict lockdown measures established in Italy between March and April 2020 to contrast the spread of the new Coronavirus disease created the unique opportunity to observe air quality trends in an almost trafficless environment and consequently to evaluate the impact of traffic on air pollution. A lot of case studies related to different cities have been reported, with different results. In our paper, we present a case study related to the city of Pavia, Italy, located in one of the most polluted areas in Europe.

Thanks to a dense network of sensors deployed throughout the city, we were able to analyze air quality patterns with extremely high spatial and temporal resolutions using a combination of statistical analysis and linear mixed-effects models, and compare them with the same period of the previous year, before COVID-related restrictions. Our analyses included the influence of meteorological variables to avoid their confounding effects. Our results show that despite the traffic reduction, there was not a significant reduction of PM2.5 and PM10 in Pavia, but the daily pollution patterns changed in most city's locations.

All authors and have approved the manuscript and agree with its submission to PLOS ONE. All authors have contributed to the study reported.

We have no conflict of interest to disclose and we declare that this manuscript has not been published elsewhere and is not currently under consideration by any other journal.

We hope you will find our work interesting and consider our paper for publication.

Sincerely,

Daniele Pala, PhD
University of Pavia
Laboratory of Biomedical Informatics
Via Ferrata 3, Pavia, Italy 27100
daniele.pala@unipv.it
+39-334-930-4887

Manuscript Click here to access/download;Manuscript;Manuscript.docx

Impact of COVID-19 Lockdown on PM Concentrations in an Italian Northern City: a year-by-year Assessment

D.Pala[1*], V.Casella[2], C.Larizza[1], A.Malovini[3], R.Bellazzi[1]

[1]Department of Electrical, Computer and Biomedical Engineering, University of Pavia, Pavia, Italy.

[2]Department of Civil Engineering and Architecture, University of Pavia, Pavia, Italy.

[3]Istituti Clinici Scientifici Maugeri SpA SB IRCCS, Pavia, Italy.

*Corresponding author

E-mail: daniele.pala@unipv.it

Abstract

In the last century, the increase in traffic, human activities and industrial production have led to a diffuse presence of air pollution, which causes an increase of risk of several health conditions such as respiratory diseases. In Europe, air pollution is a serious concern that affects several areas, one of the worst ones being northern Italy, and in particular the Po Valley, an area characterized by low air quality due to a combination of high population density, industrial activity, geographical factors and weather conditions. Public health authorities and local administrations are aware of this problem, and periodically intervene with temporary traffic limitations and other regulations, often insufficient to solve the problem. In February 2020, this area was the first in Europe to be severely hit by the SARS-CoV-2 virus causing the COVID-19 disease, to which the Italian government reacted with the establishment of a drastic lockdown. This situation created the condition to study how significant is the impact of car traffic and industrial activity on the pollution in the area, as these factors were strongly reduced during the lockdown. Differently from other areas in the world, a drastic decrease in pollution measured in terms of particulate matter (PM) was not observed in the Po Valley during the lockdown, suggesting that several external factors can play a role in determining the severity of pollution. In this study, we report the case study of the city of Pavia, where data coming from 23 air quality sensors were analyzed to compare the levels measured during the lockdown with the ones coming from the same period in 2019. Our results show that, on a global scale, there was a statistically significant reduction in terms of PM levels taking into account meteorological variables that can influence pollution such as wind, temperature, humidity, rain and solar radiation. Differences can be noticed analyzing daily pollution trends too, as – compared to 2019 – during 202[illegible]llution was higher in the morning and lower in the remaining hours.

Introduction

In the last decades, air pollution has become a major threat to health and climate in several countries of the world, most of them being urban or highly populated areas. Scientific investigations showed that exposure to air pollution can lead to an increased risk of developing or exacerbating several diseases, in particular respiratory and cardiovascular ones [1]. The prevalence of diseases such as asthma, type 2

diabetes and cardiovascular disorders is also increasing in most of the world [2–4]. While public health organizations, together with non-profit associations and governments are struggling to apply "green solutions" to contain the increase in pollution levels, a sudden reduction of air pollutants concentration was observed in many countries between the end of the year 2019 and the year 2020. During this period, most of the productive activities throughout the world had to come to an unexpected stop due to the pandemic caused by the new SARS-CoV-2 virus, and the related diseases, COVID-19. This virus generated most likely in a market in Wuhan, China, and quickly spread causing a high number of intense flu-like syndromes and cases of atypical pneumonia. Compared to influenza viruses, this unknown pathogen showed abnormally high contagious strength, with higher hospitalization and death risk [5]. Despite the initial efforts to contain the disease, the virus spread in several Asian countries outside of China, and at the end of February 2020 the first European case unrelated to the Asian outbreak was found in northern Italy, in the city of Codogno in Lombardy. In the following weeks, Italy suffered a tremendous increase in terms of COVID-19 cases, forcing the government to take drastic actions. Thus, Italy was the first western country to apply severe measures and to apply a general lockdown, with most of the population confined at home and a shutdown of all nonessential productive activities and services.

The first area where schools and factories have been closed included the most densely populated and industrialized regions of northern Italy, located in a large area named Po Valley. Among other aspects, this area is known to be among the most polluted in Europe [6], since several air pollutants, including particulate matter, often rise to dangerous levels. This phenomenon is due to an unfortunate combination of factors such as high population density, intense industrial activity and geographic position, as the valley is surrounded on three sides by mountains. Starting from China in December 2019, a reduction of human related pollution was observed in all areas where the lockdown was applied by governments. In several parts of the world air pollution dropped to the lowest levels in decades [7,8]; this happened particularly in Asian countries such as China and India. In Italy, although a similar effect has been observed, the reduction in terms of concentration was not evident for all pollutants. A significant decrease in nitrogen dioxide and sulfur dioxide was observed during the first period, whereas no drop in particulate matter (PM10 and PM2.5) was recorded [9]. Peculiar meteorological conditions such as high concentration of sea salt and Saharan sand brought by Eastern winds at the beginning of the lockdown and a coincidental increase in the wind speed for several days could have contributed to this phenomenon [10]. Meteorological factors are known to play a considerable role in modulating particulate matter and other pollutants concentration: wind and precipitations tend to disperse them, while lower or higher temperatures can create favorable conditions for higher concentrations. The presence of confounders makes it difficult to assess the real impact of traffic, industrial activities and house heating on the pollution levels, especially because they can potentially interact in complex ways. The sudden lockdown condition in which Italy entered between February and March 2020 allowed to analyze how vehicular traffic and factories activities impact on air pollution during the daily life. Although traffic was limited to essential transportations and factories not producing essential goods were closed during this period, results from studies resulted controversial due to the extreme variability in terms of weather condition typically characterizing the month of March [11]. Air pollution is a typical problem in the Po Valley during winter months [12], when cold dense air tends to stagnate in the lower layers of the atmosphere. The phenomenon is less common in the warm season as air is less dense and local breezes are more frequent.

In this paper we present the results from a study performed in Pavia, located in the Po valley in Italy, where 45 air quality sensors have been deployed in the urban area in the context of the European project PULSE (Participatory Urban Living for Sustainable Environments [https://www.project-pulse.eu/]), allowing to create a dense network of pollutants measurements.

The primary objective of the analysis was to assess the impact of the lockdown on the urban PM pollution. To this aim we analyzed PM2.5 and PM10 data coming from the available sensors with high spatial and temporal resolution and compared measurements performed from the end of February to the

beginning of April of 2020 with those deriving from the same period in 2019. Analyses consisted of several steps: we first analyzed data from two official monitoring stations in Pavia, then we investigated the effect of meteorological conditions (wind and temperature) on the measurements and finally we estimated the adjusted mean variations in terms of PM2.5 and PM10 between years 2019 and 2020 at a whole data level and at a single sensor level stratified by daily hours.

Materials and Methods

The PULSE Project

New public health initiatives are emerging to face the new challenges brought by the increase of population in the urban areas and the global changes in air pollution and lifestyle. Among these, the PULSE project was funded by the European Commission to perform heath research in big cities across Europe, Asia and the U.S.A. through a collaboration between universities, research centers and city councils. The project started during the fall of 2016 and lasted until April 2020 with clinical focus on the link between air quality and asthma, physical inactivity and type 2 diabetes and on the influence of exposures on health and wellbeing in general. A complex multi technological platform was created in the context of the PULSE project and tested in seven cities: Barcelona (Spain), Birmingham (United Kingdom), Keelung (Taiwan), New York (U.S.A.), Paris (France), Pavia (Italy) and Singapore. This platform is based on a sophisticated data exchange infrastructure that involves data coming from different sources, including satellite data, open data and dedicated sensing technology for air quality.

The sensors network in Pavia

The city of Pavia, Italy, was one of the test sites of the PULSE system. Although Pavia is a rather small city (about 72,000 inhabitants), its university and its peculiar position in the geographical area of the Po Valley makes it a suitable location to carry on research and innovation projects to study air quality and personal exposure to pollutants, being air pollution and its related health risk a frequent problem that affects this area during winter months.

One of the key innovation aspects of PULSE is the use of data with high spatial and temporal resolution. The increase in terms of resolution can be beneficial to study phenomena of public health interest at a neighborhood level, thus taking into account social and health disparities that often characterize large urban environments, as highlighted by studies we performed in the context of the project [13,14]. Despite this necessity, urban data is rarely collected with a sufficient spatial granularity, due to the high costs and difficulties of the process. Taking air quality as an example, pollution is usually measured by high quality monitoring stations deployed in the cities by official agencies or state departments. Being these instruments expensive and requiring constant maintenance they are usually deployed in small numbers. As an example, the entire city of New York has only 13 monitoring stations while Pavia has 2 stations over an area of about 65 km^2. For this reason, low-cost and portable sensing technologies are becoming common [15] but generating controversial results: while the frequency and easiness of measurements increase with low-cost technology, the quality of the data collected is often limited.

A total number of 45 low-cost PurpleAir sensors [16] were purchased and installed throughout Pavia in agreement with the municipality and with the collaboration of a few private citizens within the PULSE project. These sensors are small outdoor devices that measure three types of pollutants, i.e. PM1, PM2.5 and PM10, and temperature and humidity as meteorological data. These instruments are easy to use as they can be installed on private apartments' balconies and require only a Wi-Fi connection and an electrical outlet. The network created by these sensors was added to already existing official monitoring stations, property of *ARPA Lombardia* (Environmental Protection Regional Agency of the region of Lombardy), a public agency that aims at measuring environmental data in the Lombardy region in Italy

– where Pavia is located. Through numerous sensors scattered throughout the region, ARPA collects a large quantity of data about air quality, meteorology, agriculture, sole status, etc.

In Pavia, there are two official ARPA air quality monitoring stations, they are high-quality fixed stations that measure several pollutants (NO_X, SO_2, CO, O_3, PM10 and PM2.5) at regular time intervals. These sensors are calibrated continuously using commercially available instruments that can be used as reference according to Italian or European laws [17] and therefore can be considered very reliable. Weather parameters are collected as well, and data is freely available upon request on a dedicated portal. An analysis has been performed to evaluate whether measurements performed by the Purple Air sensors were different from the ones performed by one of the official monitoring stations belonging to the local environmental agency ARPA. It was observed that, despite a small offset, the Purple Air sensors' measurements were highly correlated with the official ARPA ones (correlation > 0.9). Thanks to the large number of available devices, a dense sensors network was created, allowing to derive high-quality homogeneous maps through interpolation and to use them in the monitoring of pollution levels and personalized risk for the citizens that move around the city. Besides defining a spatially dense network, these sensors allow also for high temporal granularity in the measurements, by quantifying PM1, PM2.5, PM10 every 80 seconds. The geographical position of the Purple Air sensors located in Pavia can be visualized on the real time map available on the Purple Air website, showing the latest sensors' measurements [18].

Analyzed data

Two main data sources have been used in this study: air quality measurements coming from the Purple Air sensors and weather data (wind speed, temperature, humidity, precipitations and solar radiation) acquired from the ARPA official database. In particular, used sensors measure all kinds of particulate matter (PM1, PM2.5 and PM10), but only PM2.5 and PM10 have been considered in our analysis, since they are well-agreed indexes of air pollution. Even though the Purple Air sensors themselves measure weather data (temperature and humidity) , these data were not of interest in the PULSE project and therefore the accuracy of weather measurements was not determined. For this reason, we decided to use the official ARPA sensors to measure meteorological variables included in the study. Being Pavia a small city, it is not expected to observe significant meteorological differences across different areas of the city.

We considered hourly pollution measurements data during two periods of time: from February 24th, 2020 (00:00 CET) to April 2nd, 2020 (24:00, CEST) and the same period during year 2019. During 2020 the time period considered started in correspondence of the days after the identification of the first COVID-19 cases in northern Italy, with the consequent closing of schools and of most of the commercial activities and the deriving reduction in terms of urban mobility (the so-called "lockdown"). The same temporal period during year 2019 has been considered as reference, to estimate PM2.5 and PM10 variations between the two years. Meteorological data (measurements of hourly average wind speed, maximum wind gusts, air temperature, humidity, precipitations and solar radiation) were collected with the same hourly temporal granularity as PM. Only sensors with available measures for both 2019 and 2020 were included in the analysis.

Data calibration

Data measured by Purple Air sensors were calibrated using the official ARPA monitoring stations measurements, using data of a single sensor located close to one of the ARPA stations (PurpleAir sensor 3, PA-S3). Linear regression was used to calibrate the sensor data according to the following model:

$$y = m \cdot x + b$$

Where y indicates data coming from the Purple Air sensor, x those coming from the ARPA monitoring station, and m and b are the regression coefficients, representing the slope and the intercept respectively. Using measurements with the same temporal granularity (measurements were aggregated by day as the ARPA data was available only with this temporal granularity), the values of m and were estimated for PM2.5 and PM10 independently. Data from the considered sensor and from the ARPA station showed evidence of strong correlation (r = 0.83 and r = 0.80 for PM2.5 and PM10 respectively). Once the parameters were estimated, all sensors' measurements were corrected by the inverse formula:

$$\hat{x} = \frac{(y - b)}{m}$$

Specifically, by comparing the PM2.5 measurements between the Purple Air and the ARPA sensors we obtained m = 1.5755 and b = -5.7709, while m = 1.1851 and b = -16.1240 when comparing PM10 values. Thus, by defining PM2.5' and PM10' as the crude values measured by our sensors, the corresponding scaled PM2.5 and PM10 values have been calculated as follows:

- PM2.5 = (PM2.5' + 5.7709) / 1.5755
- PM10 = (PM10' + 16.1240) / 1.1851

Scaled PM2.5 and PM10 measurements were then used for the analyses reported in the next sections.

Considering that the Purple Air sensors have all the same hardware, it was enough to locate only one sensor close to the ARPA one to estimate the correction to be made, and then apply it to all the other Purple Air sensors, rather than co-locate all sensors close to the ARPA ones. Furthermore, an internal study performed at the University of Pavia locating seven Purple Air sensors in the same place showed evidence of strong correlation between measures performed by co-located sensors. Results are reported in **Supplementary Figures 1-4**, showing the correlation matrices and the range of measured values for each sensor on a selected day (July 24th 2019). Considering that Pavia is a small city with no significant climatic differences across different zones, we assumed that possible changes in performance over time should not affect some sensors more than others.

Data pre-processing

Only data from 24 out of the 45 Purple Air sensors available in Pavia were analyzed since some of these were not active during both years 2019 and 2020. Scatterplots and boxplots by sensor were generated to visually inspect the correlation between PM.2.5 and PM10 concentration (μg/m^3) and to identify potential outlier measurements. Visual inspection of boxplots allowed identifying and removing 4 outlier values corresponding to PM10 and PM2.5 > 250, for a total number of 39,926 measurements passing quality control.

General pollution trend over the years

Before comparing 2019 and 2020 data by statistical methods, it is useful to check for the presence of potential increasing or reducing trends in PM levels in the study area. The continuous technological improvements in vehicular traffic and energetic efficiency are presumably leading to a slow decrease of average pollution levels in Europe, and this could be a confounding element in the evaluation of the lockdown effects using data from the previous year, since an observed reduction could be due to the general trend instead of the lockdown itself. According to data in literature and past articles, there is some evidence of a PM10 and PM2.5 reduction trend in all the area in the last years [19] although with notable fluctuations. Looking at an article published by the Italian National Environmental Protection System [20], it can be noticed that the reduction trend appears less evident after 2018, with even a little increase, probably not significant, in the PM10 concentrations in 2020. The article itself states that the meteorological variability could have played an important role in the measurements' variations, as in 2019 and 2020 temperatures were generally higher and precipitations lower than the previous years.

Therefore, it is reasonable to assume that the general trend characterizing the last years probably did not significantly influence the difference in PM2.5 and PM10 concentrations between 2019 and 2020.

Statistical and data mining methods

The Wilcoxon Mann Whitney test was applied to compare numeric variables distribution between groups. Multivariate regression trees were fitted to identify informative wind speed cut off values able to discriminate subpopulations of PM2.5 and PM10 measurement values. The one sample t-test was applied to test the null hypothesis that the mean variation in terms of PM pollutants between years was zero. The Spearman correlation coefficient *r* and corresponding 95% confidence interval (95% CI) were computed to estimate the correlation between numeric variables by a bootstrap approach based on 1,000 replicates. Linear mixed model regression was used to estimate the adjusted mean variation in terms of PM2.5 and PM10 between years 2019 and 2020, using data matched between years by measurement month, day and hour. Year (binary: 2019 coded "0", 2020 coded "1"), working day (binary: week end coded "0", working day coded "1"), wind (numeric continuous, expressed as m/s), temperature (numeric continuous, expressed as °C), humidity (numeric continuous, expressed as %), precipitations (numeric continuous, expressed as mm), solar radiation (numeric continuous, expressed as W/m^2) and sensors elevation upon the sea level (continuous, expressed as meters) were included as fixed effect terms, while a single variable resuming the sensors ID/month/day/hour of measurement was used as random effect grouping variable. The interaction between year and daily hour was also included in order to estimate the adjusted mean variation in terms of pollutants concentration by daily hours interval. Analyses were performed at a whole data level and then repeated by sensor.

An alternative approach consisting of a modification of the method proposed by Venter et al. [21] has been also applied to estimate the potential impact of the lockdown on pollutants concentration during year 2020. The method is described in the detail in the **Supplementary Material** section.

Analyses were performed by the R statistical software tool version 3.6.1 (www.r-project.org). Regression trees were fitted by the *rpart* fuction implemented in the *rpart* package. Spearman coefficients and corresponding 95% CI were computed by the *spearman.ci* function implemented in the R package called *RVAideMemoire*. Linear mixed model analysis was performed by the *lmer* function implemented in the R package called *lme4*. MATLAB (R2020b Version) was used for sensors' calibration, analyses regarding the official ARPA data and the creation of visual representations of the PMs trends in the two years.

Results

Analysis of the Official ARPA Data

ARPA owns two sensors in the urban area of Pavia: the first one (ARPA-S1) is located in a square very close to the city center and along one of the most important traffic arteries of the city, characterized by high traffic during the rush hours. The second one (ARPA-S2) is located in a residential area, densely populated but less prone to traffic. Unfortunately, ARPA historical data are available with a daily temporal granularity for PM10 only. As a matter of fact, ARPA-S1 open data does not contain PM2.5 measurements and ARPA-S2 open data are characterized by an extremely high missing data fraction in the considered period.

When comparing measurements distribution between years it was possible to observe different behaviors of the two sensors, as ARPA-S1 showed a significant decrease in the PM10 levels during the year 2020 (median decreasing from 42 µg/m^3 to 26 µg/m^3, p-value = 0.0016), whereas ARPA-S2 measurements distribution did not show significant variations (p-value = 0.4571). The difference between the two sensors is visible also in **Fig 1**, where PM10 measurements of both sensors in 2019

and 2020 are shown. This analysis was performed on daily averaged data for each sensor, the only data available from the ARPA dedicated portal.

These differences are presumably due to the fact that pollution levels are highly dependent on meteorology, and spring months like March are often characterized by very quick weather changes (in terms of wind, humidity, pressure, temperature, etc.). This enlightens the necessity to take meteorological factors into account when comparing two time periods.

Fig 1 – PM10 daily trends during 2019 and 2020 measured by the two ARPA monitoring stations.

Besides PM2.5 and PM10, when focusing on other kinds of pollutants measured by ARPA sensors (CO, NO_2, SO_2 and SO) it has been possible to observe a statistically significant reduction in terms of all pollutants except for CO during 2020 compared to 2019 ($p < 0.0001$) (**Table 1**).

Pollutant	Median 2019 (Q1:Q3)	Median 2020 (Q1:Q3)	p-value
CO	0.6 (0.6:0.7)	0.7 (0.5:0.8)	0.1295
NO_2	34.0 (20.6:47.9)	20.9 (13.3:31.7)	<0.0001
SO_2	5.2 (4.3:6.1)	1.8 (1.4:2.3)	<0.0001
SO	42.7 (27.7:64.5)	28.2 (18.0:42.5)	<0.0001

Table 1. Results of the Wilcoxon Mann Whitney test on ARPA data. The table reports the median values of the pollutants in the considered periods in 2019 and 2020, together with their 25th and 75th percentiles, and the tests' p-value. Apart from CO, all pollutants had a significant decrease in 2020 compared to 2019, even neglecting the effect of meteorological variables.

Effect of potential confounders on pollutants concentration

After outliers removal a total number of 39,926 measurements passed quality control. An exploratory analysis has been performed to inspect how the pollution trends have changed during the different time periods and how weather conditions influenced this change. Looking at the absolute quantities reported in **Fig 2**, showing scaled data from the sensor PA-S3 (the same used for data calibration), a very irregular trend in pollution concentration can be observed during both years, suggesting that external factors can influence these measurements.

Fig 2 – PM2.5 concentration and wind speed in the two considered periods in 2019 (upper plot) and 2020 (lower plot).

Looking at the two plots, it is possible to observe that high wind speed peaks correspond generally to lower concentrations of PM2.5. The same trend has been observed for PM10 (**Supplementary Figure 5**).

Similarly, the effect of temperature on PM concentration (scaled values) has been inspected. Plots in **Fig 3** and **Supplementary Figure 6** show an apparently milder relationship between temperature and pollutants concentration.

Fig 3 – PM2.5 concentration and temperature in the two considered periods in 2019 (upper plot) and 2020 (lower plot).

The relationship between wind speed (m/s), temperature (°C), humidity (%), precipitations (mm), solar radiation (W/m^2) and pollutants' concentration using scaled data pooled from both 2019 and 2020 was assessed by visual inspection of the scatterplots reported in **Fig 4**

Fig 4 – Scatterplots describing the correlation between wind speed, temperature, humidity, precipitations, solar radiation and PM concentration.

Plots in **Fig 4A** and **Fig 4F** confirm a mild negative correlation between wind speed and both PM2.5 and PM10 levels (PM2.5: r = -0.40, 95% CI = -0.41:-0.39; PM10: r = -0.40, 95% CI = -0.41:-0.39), especially for high wind speed values. High wind speed values could reduce pollutants concentration, representing a potential confounder when comparing PM levels between 2019 and 2020. Further, PM2.5 and PM10 levels correlated positively with humidity (PM2.5: r = 0.47, 95% CI = 0.46:0.48; PM10: r = 0.47, 95% CI = 0.46:0.47, **Fig 4C** and **Fig 4H**).

Weaker and negative correlation was observed between temperature and pollutants concentration (PM2.5: r = -0.21, 95% CI = -0.22:-0.20; PM10: r = -0.20, 95% CI = -0.21:-0.19, **Fig 4B** and **Fig 4G**) and between pollutants concentration and solar radiation (PM2.5: r = -0.19, 95% CI = -0.20:-0.18; PM10: r = -0.19, 95% CI = -0.20:-0.18, **Fig 4E** and **Fig 4J**).

The evidence of correlation between precipitations and pollutants concentration was almost null (PM2.5: r = 0.02, 95% CI = -0.01:0.03; PM10: r = 0.02, 95% CI = 0.01:0.03, **Fig 4D** and **Fig 4I**).

Based on the scatterplots in **Fig 4** it was possible to observe a non linear relationship between wind speed and pollutants concentration (**Fig 4A** and **Fig 4F**). With the aim to identify informative wind speed cut off values able to distinguish subpopulations of measurements, univariate regression trees were fitted including wind speed as predictor while PM2.5 and PM10 as dependent variables in turn.

By visual inspection of the cross-validation results of the unpruned trees it was possible to observe that the first split caused a major reduction of the relative cross validation error of about 10% for both pollutants, further splits induced less relevant reductions (~ 2%) as shown in **Supplementary Figure 7**. Thus, by imposing a single split to the regression tree algorithm, a wind speed of 2.15 m/s was identified as the most informative threshold to stratify both PM2.5 and PM10 levels. Pollutants concentration measured when the wind speed was ≥ 2.15 m/s were significantly lower compared to those performed when the wind speed was below the threshold (p-value < 0.0001).

Plots in **Fig 4C** and **Fig 4H** evidenced that pollutants concentration did not vary with respect to humidity when humidity values were below ~ 20%, highlighting a potential bias in terms of measurements accuracy when the confounder value is below this threshold.

It was then decided to focus on a subset of 22,240 measures performed when the wind speed was below 2.15 m/s and humidity > 20% to avoid confounding effects.

Matching measurements between years

PM measures were then matched between 2019 and 2020 by sensor, month, day and hour to further reduce the potential impact of confounders when assessing their variation between years. Further, all data from a single sensor (PA-S24) were also removed due to the low number of measurements available compared to the others (n = 154 vs. n ≥ 436). A total number of 5,452 paired measures (year 2019: 5,452 measures, year 2020: 5,452 measures) from 23 sensors were included in the analyses on

the basis of the matching criteria. **Supplementary Table 1** reports the number of measurements available by sensor.

The correlation between PM2.5 and PM10 measures during 2019 and 2020 as well as the correlation between variations in terms of PM2.5 and PM10 between 2019 and 2020 using matched data was extremely high (Spearman r > 0.99), as shown in **Fig 5** and **Fig 6**.

Fig 5 – Correlation between PM2.5 and PM10 during years 2019 and 2020.

Fig 6 – Correlation between variations in terms PM2.5 and PM10 between year 2019 and 2020.

Variation in terms of pollutants concentration between 2019 and 2020

The variation in terms of PM2.5 and PM10 concentration between 2019 and 2020 has then been estimated, results are reported in **Table 2** and show no statistically significant variations in terms of mean PM2.5 (p-value = 0.177) or PM10 (p-value = 0.230).

		**PM2.5**		
**Year**	**N**	**Mean ± SD**	**Median (Q1:Q3)**	**p**
2019	5,452	34.2 ± 21.02	34.6 (15.63 : 50.79)	
2020	5,452	33.68 ± 23.69	28.11 (17.55 : 45.12)	
Variation	5,452	-0.52 ± 28.56	-0.38 (-17.92 : 17.33)	0.177

		**PM10**		
**Year**	**N**	**Mean ± SD**	**Median (Q1:Q3)**	**P**
2019	5,452	34.62 ± 19.18	34.76 (17.69 : 49.74)	
2020	5,452	34.2 ± 21.76	28.87 (19.5 : 44.18)	
Variation	5,452	-0.43 ± 26.19	-0.14 (-16.12 : 15.73)	0.230

Table 2 – PM2.5 and PM10 distribution during 2019 and 2020. Year = analyzed year or variation between years; Mean ± SD = mean value and standard deviation of pollutants concentration during years 2019, 2020 and variations between 2019 and 2020; Median (**Q1:Q3**) = median value and 25th : 75th percentiles of pollutants concentration during years 2019, 2020 and variations between 2019 and 2020; p = p – value from the paired samples t-test.

Two multivariate linear mixed effects model regression models have been fitted to quantify the variation in terms of PM2.5 and PM10 between 2019 and 2020, adjusting by wind speed, temperature, humidity, precipitations, solar radiation, weekend/working days and sensors elevation upon the sea level (fixed effects). A single random effect grouping factor was included, resuming the information about sensor ID and year, month, day, hour when the measure was performed (5,452 levels or intercept values).

Results are reported in **Table 3** and show statistically significant reductions in terms of PM2.5 and PM10 values during 2020 compared to 2019 accounting for potential confounders (PM2.5: Year (2020)

coefficient = - 2.58 μg/m^3, 95% CI = -3.25 to -1.91 μg/m^3, $p < 0.0001$; PM10: Year (2020) coefficient = - 2.25 μg/m^3, 95% CI = -2.87 to -1.64 μg/m^3, $p < 0.0001$).

	PM2.5		PM10	
**Variable**	**Estimate (95% CI)**	**p**	**Estimate (95% CI)**	**p**
Year (2020)	*-2.58 (-3.25:-1.91)*	*<0.0001*	*-2.25 (-2.87:-1.64)*	*<0.0001*
Working day (yes)	11.15 (10.36:11.94)	<0.0001	10.15 (9.42:10.87)	<0.0001
Average wind speed (m/s)	-4.33 (-4.99:-3.66)	<0.0001	-3.96 (-4.57:-3.36)	<0.0001
Temperature (°C)	1.59 (1.48:1.7)	<0.0001	1.51 (1.4:1.61)	<0.0001
Sensors' elevation upon the sea level (m)	0.02 (-0.04:0.08)	0.4850	0.03 (-0.03:0.08)	0.3002
Humidity (%)	0.54 (0.52:0.57)	<0.0001	0.49 (0.47:0.52)	<0.0001
Precipitations (mm)	-9.55 (-11.2:-7.9)	<0.0001	-8.85 (-10.36:-7.34)	<0.0001
Solar radiation (W/m^2)	0 (-0.01:0)	0.0015	0 (-0.01:0)	0.0001

Table 3 – Multivariate linear mixed effects model regression: variation in terms of PM2.5 and PM10 between 2019 and 2020 accounting for confounders. Estimate (95% CI) = regression coefficient and 95% CI; p = p-value. The regression coefficient corresponding to "year (2020)" term quantifies the variation in terms of PM2.5 and PM10 pollutants concentration (μg/m^3) between 2019 and 2020 accounting for the other variables included in the model reported in the table.

Variation in terms of pollutants concentration between 2019 and 2020 by daily hours

The presence of differences in terms of PM variations between 2019 and 2020 has been then inspected by daily hour intervals by including the year x hour interaction and the same set of confounders previously described in the regression. Adjusted means estimated by multivariate linear mixed effect models showed that both PM2.5 and PM10 variations were characterized by different trends as function of the daily hours interval considered (**Fig 7** and **Fig 8**).

Fig 7 – PM2.5 variations by daily hour interval. Data are presented as adjusted mean variations, unadjusted mean variations and unadjusted median variations.

Fig 8 – PM10 variations by daily hour interval. Data are presented as adjusted mean variations, unadjusted mean variations and unadjusted median variations.

By inspecting the adjusted mean variations (**Fig 7** and **Fig 8**) it is possible to observe a general reduction in terms of PM2.5 and PM10 between 2019 and 2020 during all day except from 8 to 10 in the morning, when a pollutants increase was estimated. Unadjusted mean and median variations showed conflicting trends of variations: discrepancies with respect to the adjusted mean variations could be partially explained by the non-gaussian distribution of PM measures variations during the different daily hours and by the lack of correction by confounders.

The adjusted variations estimated by linear mixed effect models in the whole sample and by sensor and daily hour (by performing sensor-level analyses) are graphically represented in **Fig 9** and reported in

Supplementary Table 2 and **Supplementary Table 3**. Results show strong consistency in terms of adjusted mean PM variations by daily hour intervals across sensors. A general pollutants increase was estimated from hour 8 to 10 in the morning for most of the sensors, in some cases increases were observed also from 10 and 12 and - sporadically - during evening/night, morning and afternoon hours.

Fig 9 – Heatmaps showing adjusted mean variations in terms of PM2.5 between 2019 and 2020 by sensor and daily hour intervals. Each heatmap row represents graphically the adjusted mean variation in terms of PM2.5 and PM10 between 2019 and 2020 over the complete set of PurpleAir sensors (All sensors) and by sensor (PA-S1 – PA-S23) over daily hours intervals defined. Shades of green are proportional to the estimated magnitude of the reduction in terms of PM2.5 between 2019 and 2020; shades of red are proportional to the estimated magnitude of the increase in terms of PM2.5 between 2019 and 2020 as showed by the colors legend on the right side of each plot. The "+" symbol denotes an increase in terms of pollutants concentration between year 2019 and 2020.

An alternative approach consisting of a modification of the method described by Venter et al. [21] has been also evaluated to assess the impact of the COVID-19 related lockdown on pollutants concentration. According to this method, positive absolute changes in terms of pollutant concentration (observed – expected) indicate an increase in terms of PM compared to the expected values while negative changes a decrease in terms of pollutants concentration compared to the expected values. Positive and negative changes can be attributed to the COVID-19 related lockdown.

Results deriving from this approach were generally concordant with respect to the main findings reported in this section (**Supplementary Table 4**, **Supplementary Figure 8**). A negative change in terms of PM2.5 and PM10 was estimated for all sensors during all daily hour intervals except for hours 8 to 10 in the morning, being characterized by a positive change for most sensors for both pollutants. PM10 showed positive changes for some sensors also during hours 10 – 12 while some sensors (PA-S7, PA-S8, PA-S13 and PA-S18) were characterized by a positive change also during afternoon/evening hours.

Discussion

Air pollution is a complex phenomenon, resulting from the combination of air particles coming from different sources, both artificial and natural. Several different pollutants, including ozone, sulfur dioxide, nitrogen dioxide and particulate matter have an impact on human health. Among these pollutants, particulate matter is often used as a reference to describe air quality, as it includes all particles within a certain diameter (e.g., PM2.5 indicates all particles that have a diameter smaller than 2.5 µm) and it has been demonstrated to have an important relation with diseases risk increase [22,23]. Reduction of exposure to air pollution is the best prevention strategy that can be applied to reduce the risk of developing respiratory diseases linked to bad air quality. The National Health Ministry of Italy estimated that living in the Po valley, a large plain region in the north of the country, leads to a significant reduction of life expectancy [24]. This area is one of the most densely populated in Europe and it is characterized by high pollution levels due to a combination of factors such as high industrial activity, high traffic density, house heating and unfortunate climatic factors. Pollution is generally high during winter months, when cold dense air tends to be pressed to the ground and the frequent presence of fog makes pollutants even more dangerous [25]. In order to reduce pollution, the administrations in the regions and the cities affected take several measures like traffic limitations or regulations on the use of house heating every year. However, the results from such restrictions are often not sufficient, as the sources of pollution are numerous, and the combined effects of the produced pollutants are difficult to predict.

With the COVID-19 pandemic, which initially had its European epicenter in northern Italy at the end of winter 2020, a strict lockdown was imposed throughout Italy, as it happened previously in some regions of China and subsequently in the rest of Europe and America, as the COVID-19 cases spread and rose. For several weeks, most of the population was confined at home and all nonessential productive activities were closed. By these measures, a large part of the pollution sources was temporally stopped, creating the ideal ground to perform scientific studies to verify whether the limitation of traffic and shutdown of some activities had an impact on global and local air pollution. A large number of studies aiming at analyzing these aspects were carried out throughout the world, with results varying depending on the geographical areas covered. Some reports showed a global reduction in several pollutants worldwide [26], while other local-level studies obtained different results. Wang et al. [27] observed a significant reduction in all pollutants in 325 cities in China, especially in the northern cities, whereas Donzelli et al. [28] performed a similar analysis over a 7 months period for 3 cities in Tuscany, Italy, and reported different results depending on the pollutant (NO_2 levels changed significantly while PMs did not). A general study performed over western Europe performed by Menut et al. [29] taking into account also meteorological variables, showed a general reduction in terms of NO_2 throughout the continent, while the reduction in terms of PMs and O_3 was limited on a general level, but with pronounced local variations. Unlike other areas of the world, a severe reduction of all the pollutants during the lockdown in the Po Valley was not observed [9], indicating that traffic may not have a huge impact on pollution and that meteorological conditions probably play a role even more important than what was usually thought.

In this study, we analyzed the case of Pavia, a city in an area with poor air quality, where a dense network of sensors had been deployed a few months before as part of the European project PULSE, that had the aim to study air quality, among other things, in order to create a collaborative preventive system to help citizens and public health operators to reduce diseases risks and improve quality of life. There are several advantages deriving from the availability of a dense sensors network, such as the possibility to monitor local conditions, estimate pollution levels in each neighborhood and to analyze temporal trends. The choice of the time period considered in the study (i.e., between February and April 2019 and 2020) was motivated by the fact that unique conditions were applied in that period in 2020, as the total lockdown that was instituted had no precedents and was never repeated in the following months. In fact, even if restrictions were kept throughout 2020, the same level of shutdown of all nonessential activities and limitation to all movements were never reached again.

The results of our study show mainly two aspects: first, the influence of climatic factors on PM levels is extremely high, second, considering this difference, there has been a mildly significant reduction in PM levels

in 2020 during the lockdown, even though pollution levels remained relatively high even without traffic and most productive activities. The latter aspect suggests that most of the relevant pollution sources in this particular geographical area are represented by the heating systems in private houses and commercial traffic, and, even with only essential services active, the peculiar climatic conditions characterized by long periods without wind are able to create dangerous levels of pollution. One interesting result is that looking at the official ARPA data alone, there is not a clear decrease in the PM levels in 2020 if all meteorological variables are not taken into account, whereas there is a definite decrease considering other pollutants. This appears to be in line with other studies conducted over Italy such as [28] and [29]. Considering the variety of pollution sources and local environmental and meteorological situations, the reasons for this are not easy to explain, but it could be assumed that this phenomenon is due to the fact that traffic, which was the major pollution source that was stopped during the lockdown, contributes less to PMs than it does to other pollutants such as SO_2 and CO_2. Furthermore, most PM produced in the Po Valley could come from some sectors of industry and agriculture that were considered essential and therefore were not limited during the lockdown and from house heating systems, especially older ones. Increased house heating could partially explain also the change in the daily patterns that was observed during the lockdown, with a high peak in the first hours of the morning, i.e. the coldest ones in the day, that without lockdowns is balanced by vehicular traffic flowing during rush hours and most productive activities active during all daily hours.

Nevertheless, pollution remains a serious problem in this region, and wide interventions are needed to mitigate health risk related to air quality in northern Italy. Traffic limitations and introduction of new propulsion technologies, such as hybrid or electric engines, certainly contribute to an improvement of this situation, but are not enough to reduce health risk to safe levels.

Funding

This work has been funded by the European Commission as part of the PULSE project (Grant Agreement GA-727816) and PERISCOPE project (Grant Agreement 101016233), coordinated by the University of Pavia.

References

1. Franchini M, Mannucci PM. Short-term effects of air pollution on cardiovascular diseases: outcomes and mechanisms. J Thromb Haemost. 2007;5(11):2169–74.

2. Aj W. Worldwide trends in asthma morbidity and mortality. Explanation of trends. Bull Int Union Tuberc Lung Dis. 1991 Jun 1;66(2–3):85–9.

3. Pearce N, Aït-Khaled N, Beasley R, Mallol J, Keil U, Mitchell E, et al. Worldwide trends in the prevalence of asthma symptoms: phase III of the International Study of Asthma and Allergies in Childhood (ISAAC). Thorax. 2007 Sep 1;62(9):758–66.

4. Boyle JP, Honeycutt AA, Narayan KMV, Hoerger TJ, Geiss LS, Chen H, et al. Projection of Diabetes Burden Through 2050: Impact of changing demography and disease prevalence in the U.S. Diabetes Care. 2001 Nov 1;24(11):1936–40.

5. Cascella M, Rajnik M, Cuomo A, Dulebohn SC, Di Napoli R. Features, Evaluation and Treatment Coronavirus (COVID-19). In: StatPearls [Internet]. Treasure Island (FL): StatPearls Publishing; 2020 [cited 2020 Jul 8]. Available from: http://www.ncbi.nlm.nih.gov/books/NBK554776/

6. S.r.l LF. Nitrogen dioxide and fine particles are threatening Po valley air quality [Internet]. [cited 2020 Jul 8]. Available from: https://vitesy.com/blog/air-pollution/nitrogen-dioxide-fine-particles-po-valley-air-quality

7. Anjum NA. Good in The Worst: COVID-19 Restrictions and Ease in Global Air Pollution. 2020 Apr 6 [cited 2020 Jul 8]; Available from: https://www.preprints.org/manuscript/202004.0069/v1

8. CNN BRP. People in India can see the Himalayas for the first time in "decades," as the lockdown eases air pollution [Internet]. CNN. [cited 2020 Jul 8]. Available from: https://www.cnn.com/travel/article/himalayas-visible-lockdown-india-scli-intl/index.html

9. Rita V. Roma e Pianura Padana, le polveri sottili PM10 non diminuiscono con il lockdown [Internet]. Galileo. 2020 [cited 2020 Jul 8]. Available from: https://www.galileonet.it/roma-pianura-padana-polveri-sottili-pm10-non-calano/

10. Inquinamento: cala il biossido di azoto, ma I venti portano le polveri sottili [Internet]. [cited 2021 May 27]. Available from: https://www.repubblica.it/ambiente/2020/03/30/news/meno_biossido_di_azoto_piu_polveri_sottili_cosa_sta_accadendo_nei_nostri_cieli-252701516/

11. Collivignarelli MC, Abbà A, Bertanza G, Pedrazzani R, Ricciardi P, Carnevale Miino M. Lockdown for CoViD-2019 in Milan: What are the effects on air quality? Sci Total Environ. 2020 Aug 25;732:139280.

12. Bigi A, Ghermandi G, Harrison RM. Analysis of the air pollution climate at a background site in the Po valley. J Environ Monit. 2012 Feb 2;14(2):552–63.

13. Pala D, Pagán J, Parimbelli E, Rocca MT, Bellazzi R, Casella V. Spatial Enablement to Support Environmental, Demographic, Socioeconomics, and Health Data Integration and Analysis for Big Cities: A Case Study With Asthma Hospitalizations in New York City. Front Med [Internet]. 2019 Apr 24 [cited 2020 May 3];6. Available from: https://www.ncbi.nlm.nih.gov/pmc/articles/PMC6499162/

14. Pala D, Caldarone AA, Franzini M, Malovini A, Larizza C, Casella V, et al. Deep Learning to Unveil Correlations between Urban Landscape and Population Health. Sensors [Internet]. 2020 Apr 8 [cited 2020 May 3];20(7). Available from: https://www.ncbi.nlm.nih.gov/pmc/articles/PMC7181035/

15. Kumar P, Morawska L, Martani C, Biskos G, Neophytou M, Di Sabatino S, et al. The rise of low-cost sensing for managing air pollution in cities. Environ Int. 2015 Feb 1;75:199–205.

16. contributors PL. PurpleAir [Internet]. Public Lab. [cited 2020 May 3]. Available from: https://publiclab.org/n/16947

17. ARPA Lombardia – Taratura degli strumenti. [Internet]. [cited 2021 Jul 31]. Available from: https://www.purpleair.com/map?opt=1/mAQI/a10/cC0#12.43/45.19247/9.16417

18. Real-time air quality map | Purple Air [Internet]. [cited 2021 Aug 06]. Available from: https://www.arpalombardia.it/Pages/Aria/Rete-di-rilevamento/Qualit%C3%A0-dei-dati/Taratura-degli-strument.aspx?firstlevel=Rete%20di%20rilevamento

19. Qualità dell'aria? In Italia è migliorata negli ultimi cinque anni. Cosa misura in Snpa. [Internet]. [cited 2021 Jul 30]. Available from: https://www.infodata.ilsole24ore.com/2021/01/31/qualita-dellaria-italia-migliorata-negli-ultimi-cinque-anni-cosa-misura-snpa/?refresh_ce=1

20. Qualità dell'aria, trend in miglioramento negli ultimi 5 anni. [Internet]. [cited 2021 Jul 30]. Available from: https://www.snpambiente.it/2021/01/29/qualita-dellaria-in-italia-trend-in-miglioramento-negli-ultimi-5-anni/

21. Venter ZS, Aunan K, Chowdhury S, Lelieveld J. COVID-19 lockdowns cause global air pollution declines. Proc Natl Acad Sci U S A. 2020;117(32):18984-18990. doi:10.1073/pnas.200685311722. Fan J, Li S, Fan C, Bai Z, Yang K. The impact of PM2.5 on asthma emergency department visits: a systematic review and meta-analysis. Environ Sci Pollut Res. 2016 Jan 1;23(1):843–50.

23. Tecer LH, Alagha O, Karaca F, Tuncel G, Eldes N. Particulate Matter (PM2.5, PM10-2.5, and PM10) and Children's Hospital Admissions for Asthma and Respiratory Diseases: A Bidirectional Case-Crossover Study. J Toxicol Environ Health A. 2008 Mar 12;71(8):512–20.

24. redazione. Air pollution shortens the life expectancy of Italians [Internet]. Science in the net. 2015 [cited 2020 Jul 8]. Available from: http://www.scienceonthenet.eu/content/article/editorial-staff/air-pollution-shortens-life-expectancy-italians/june-2015

25. Cai D-P, He Y-M. Daily lifestyles in the fog and haze weather. J Thorac Dis. 2016 Jan;8(1):E75–7.

26. Zander SV, Kristin A, Sourangsu C, Jos. COVID-19 lockdowns cause global air pollution declines. Proceedings of the National Academy of Sciences. Aug 2020, 117(32):18984-18990; DOI: 10.1073/pnas.2006853117

27. Wang M, Liu F, & Zheng M. Air quality improvement from COVID-19 lockdown: evidence from China. Air Qual Atmos Health. 2020. https://doi.org/10.1007/s11869-020-00963-y

28. Donzelli G, Cioni L, Cancellieri M, Llopis Morales A, Morales Suárez-Varela MM. The Effect of the Covid-19 Lockdown on Air Quality in Three Italian Medium-Sized Cities. Atmosphere. 2020; 11(10):1118. https://doi.org/10.3390/atmos11101118

29. Laurent M, Bertrand B, Guillaume S, Sylvain M, Romain P, Arineh C. Impact of lockdown measures to combat Covid-19 on air quality over western Europe. Science of The Total Environment. 2020; 741. https://doi.org/10.1016/j.scitotenv.2020.140426.

Supporting Information

S1 Fig. PM10 correlation matrix. Correlation matrix (Pearson's correlation) of the PM10 measurements performed by seven Purple Air sensors co-located on a selected day.

S2 Fig. PM2.5 correlation matrix. Correlation matrix (Pearson's correlation) of the PM2.5 measurements performed by seven Purple Air sensors co-located on a selected day.

S3 Fig. Range of the PM10 values measured by the seven co-located sensors.

S4 Fig. Range of the PM2.5 values measured by the seven co-located sensors.

S5 Fig. PM10 and Wind Variations in time in the considered periods in 2019 and 2020. The upper plot shows 2019 data, the lower plot shows 2020 data.

S6 Fig. PM10 and Temperature Variations in time in the considered periods in 2019 and 2020. The upper plot shows 2019 data, the lower plot shows 2020 data.

S7 Fig. Visual representation of the cross-validation results for PM2.5 and PM10. The x-axis represents the complexity parameter corresponding to different tree sizes while the y-axis represents the cross validation relative error.

S8 Fig. Heatmaps showing the median difference between observed and LMM - predicted PM2.5 and PM10 during 2020 by sensor and daily hour intervals. Each heatmap row graphically represents the median absolute differences between observed and predicted pollutants measurements by daily hours intervals. Shades of green indicate negative absolute differences between observed and predicted pollutants concentration during 2020, shades of red indicate positive absolute differences between observed and predicted pollutants concentration during 2020 as showed by the colour code legend on the right side of each plot. The "+" symbol denotes a positive absolute differences between observed and predicted pollutants concentration.

S1 Table. Number of paired measurements available by sensor and hours.

S2 Table. Adjusted mean variations in terms of PM2.5 between 2019 and 2020 by sensor and daily hours. Each PurpleAir (PA) ID corresponds to a different sensor. In green: reduction in terms of PM2.5 between 2019 and 2020; in red: increase in terms of PM2.5 between 2019 and 2020.

S3 Table. Adjusted median variations in terms of PM10 between 2019 and 2020 by sensor and daily hours. Each Purple Air (PA) ID corresponds to a different sensor. In green: reduction in terms of PM10 between 2019 and 2020; in red: increase in terms of PM10 between 2019 and 2020.

S4 Table. Root mean square error, mean absolute error and Pearson correlation coefficient r from the LMM regression method.

Figure

Click here to access/download;Figure;S1_Fig.tif

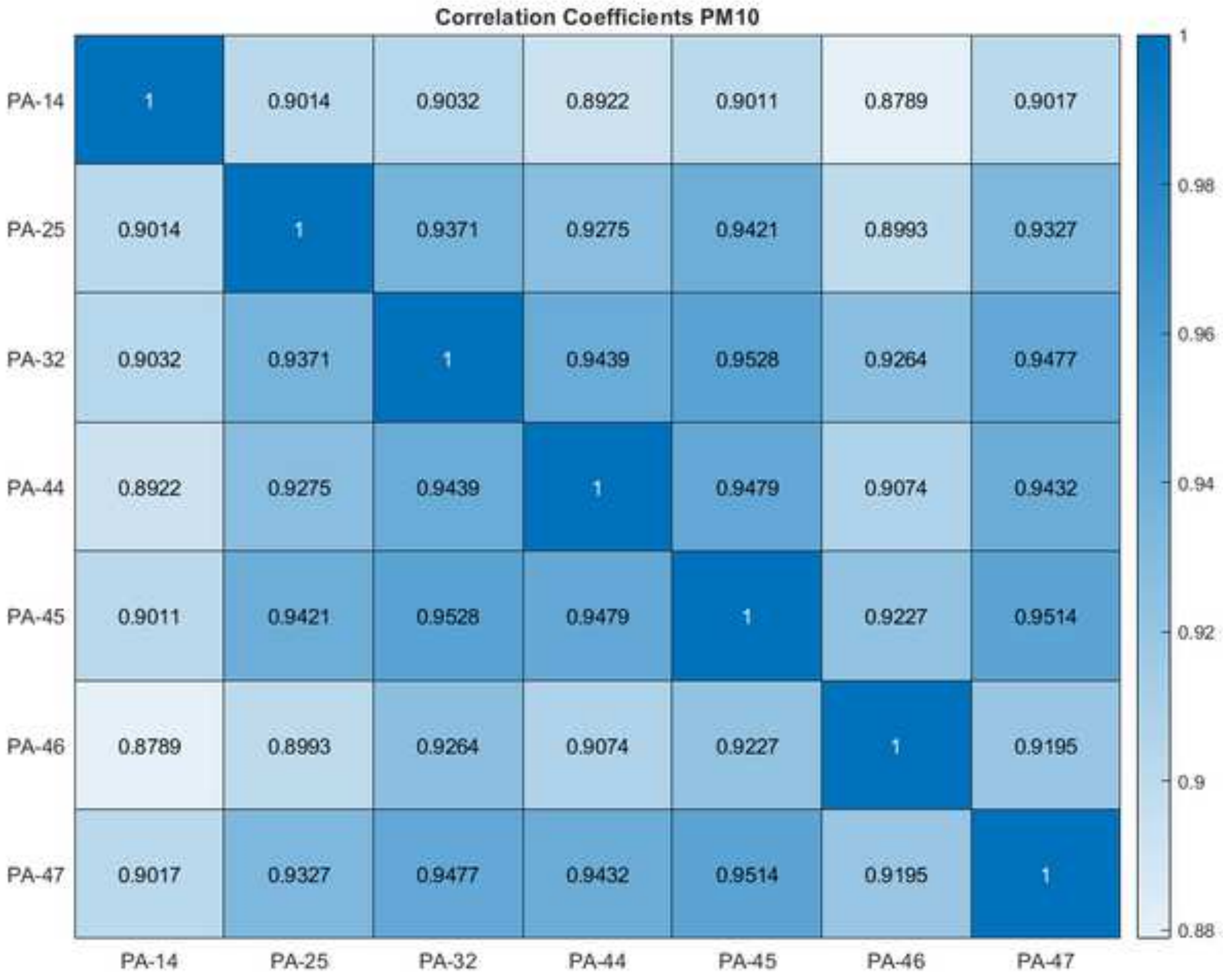

Click here to access/download;Figure;S2_Fig.tif

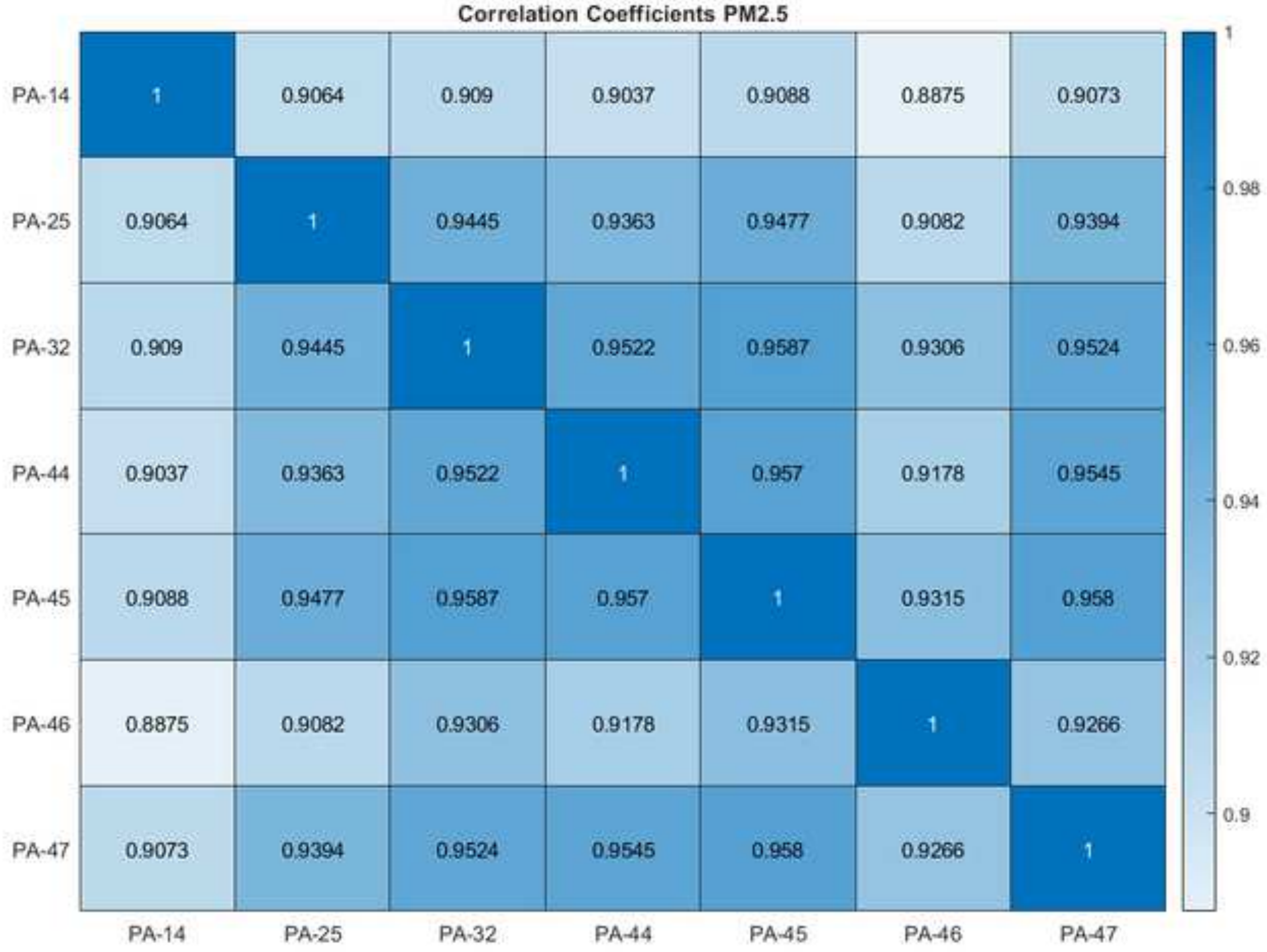

Figure

Click here to access/download;Figure;S3_Fig.tif

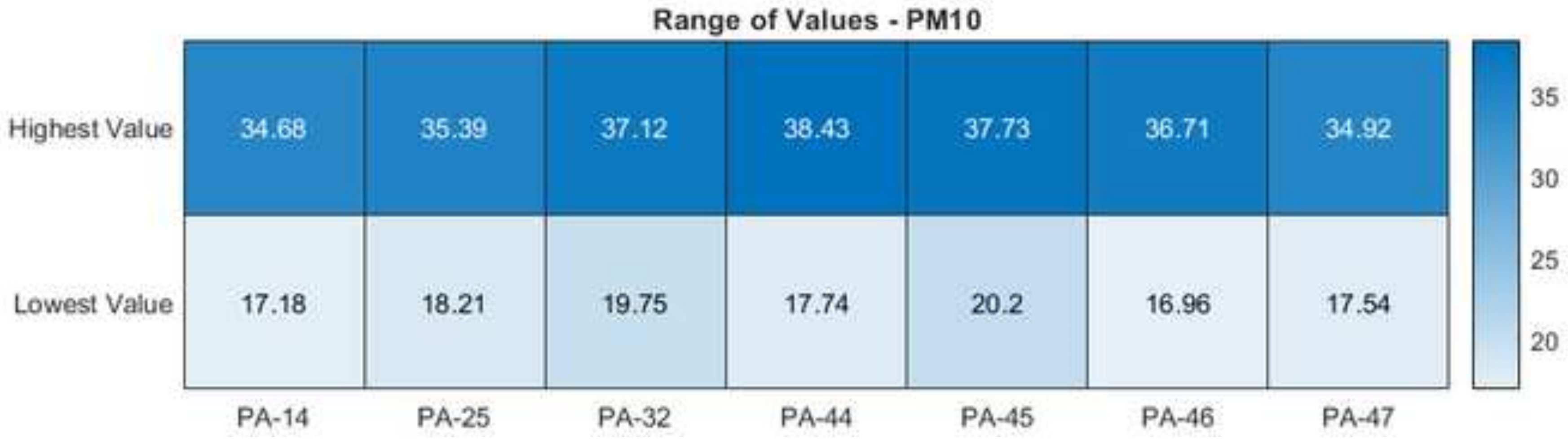

Figure

Click here to access/download;Figure;S4_Fig.tif

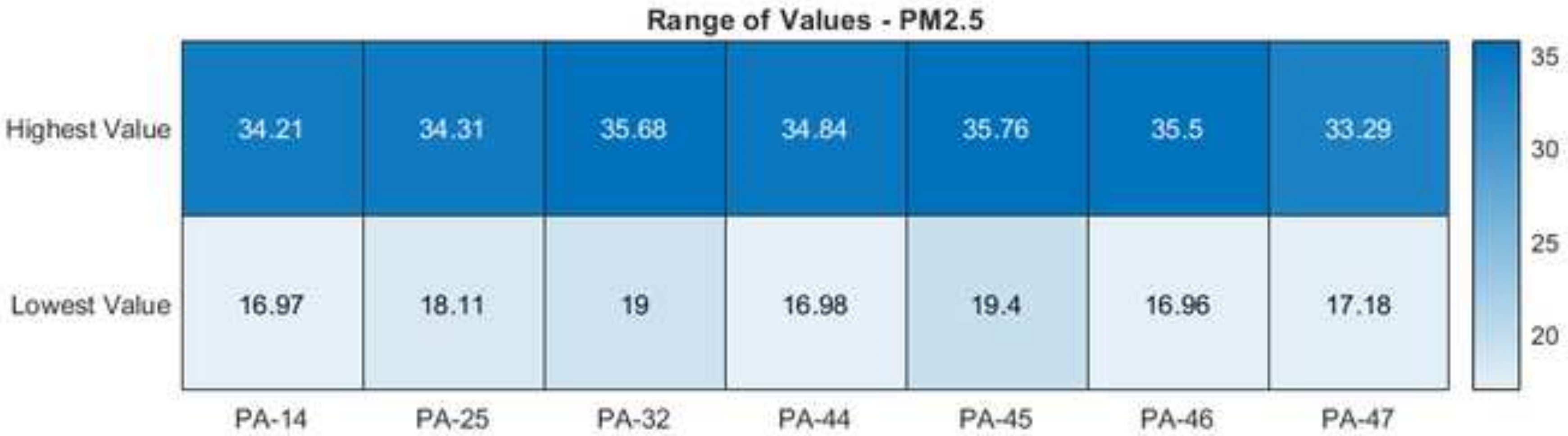

Figure

Click here to access/download;Figure;S5_Fig.tif

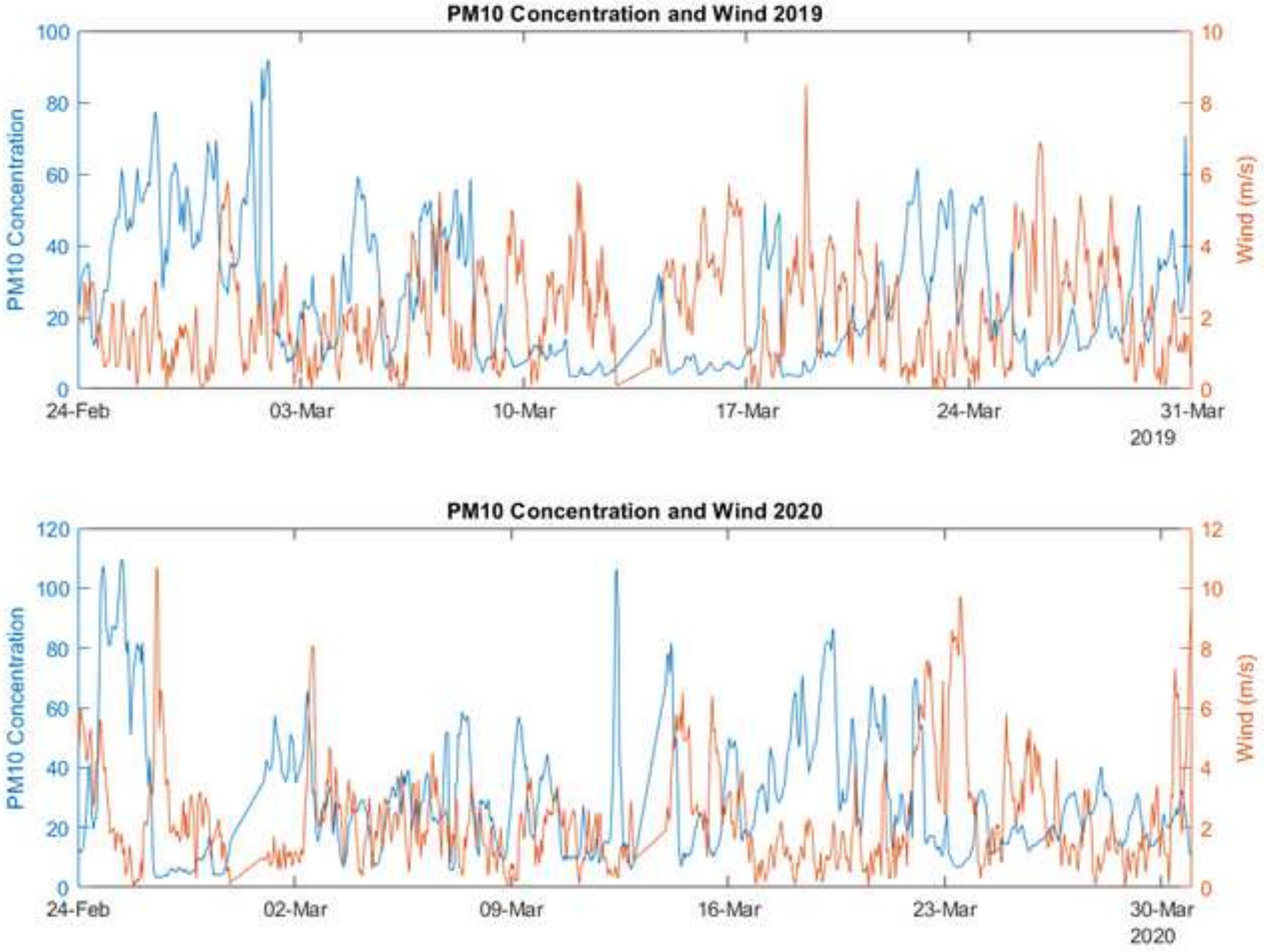

Figure

Click here to access/download;Figure;S6_Fig.tif

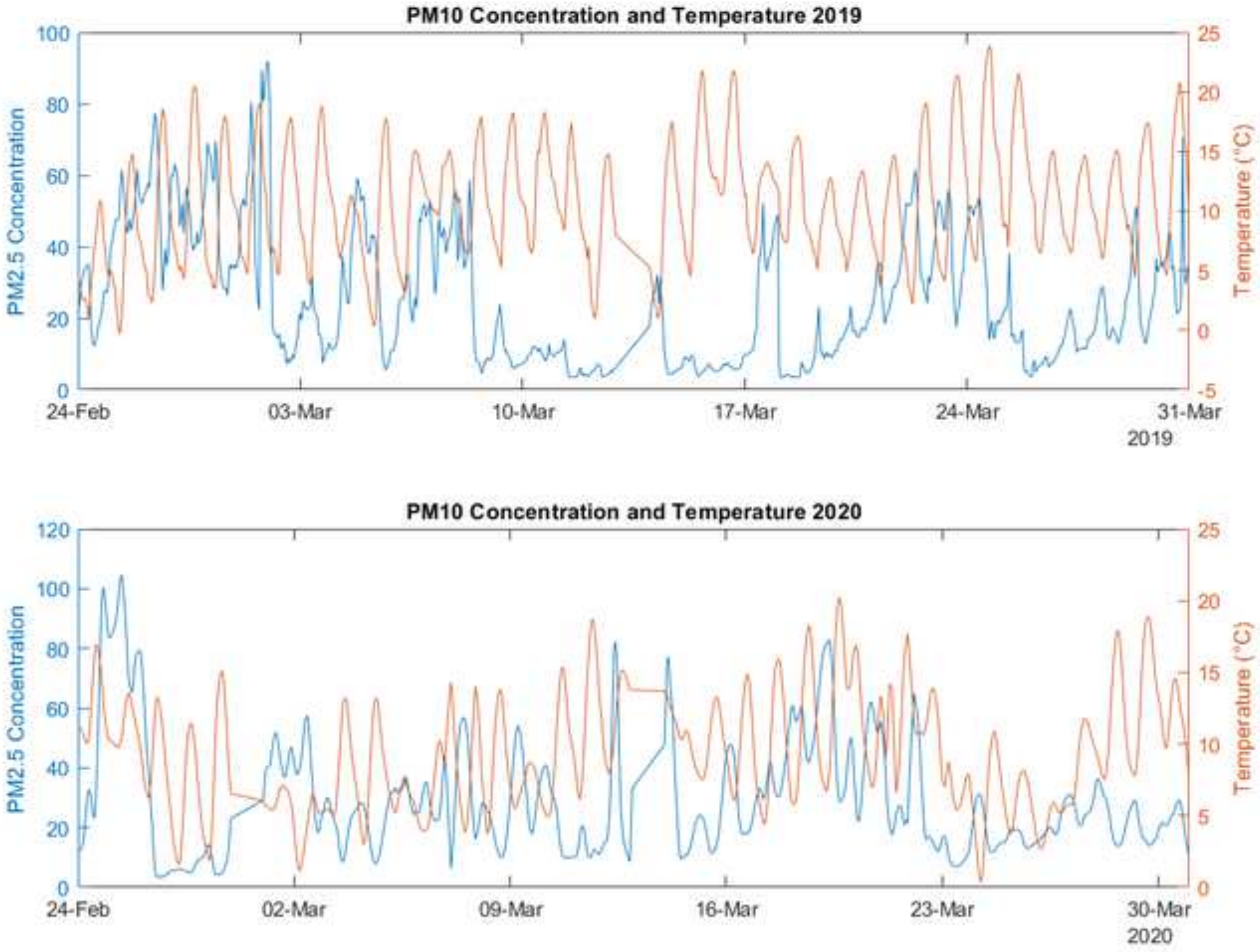

Figure

Click here to access/download;Figure;S7_Fig.tiff

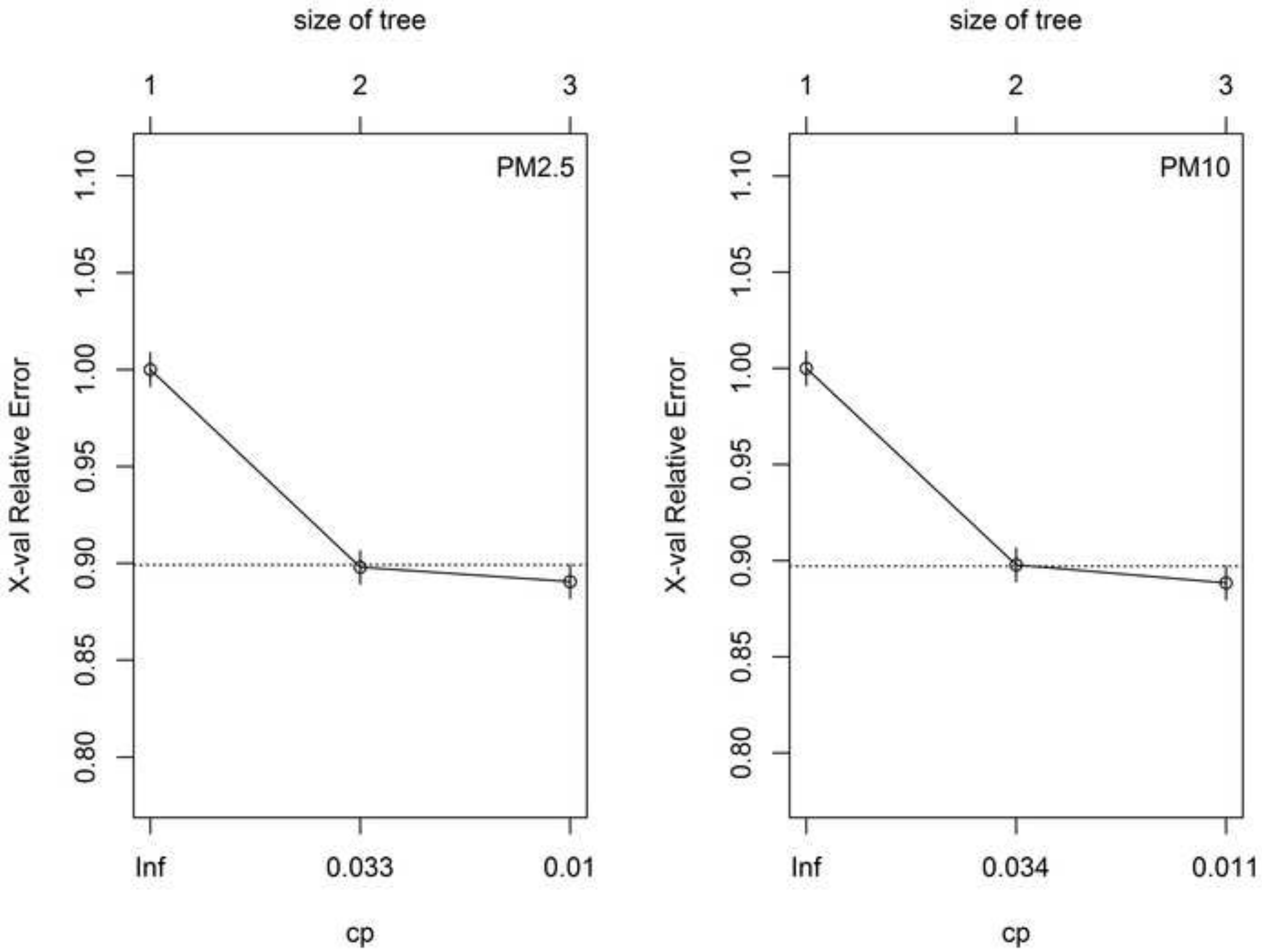

Figure

Click here to access/download;Figure;S8_Fig.tiff

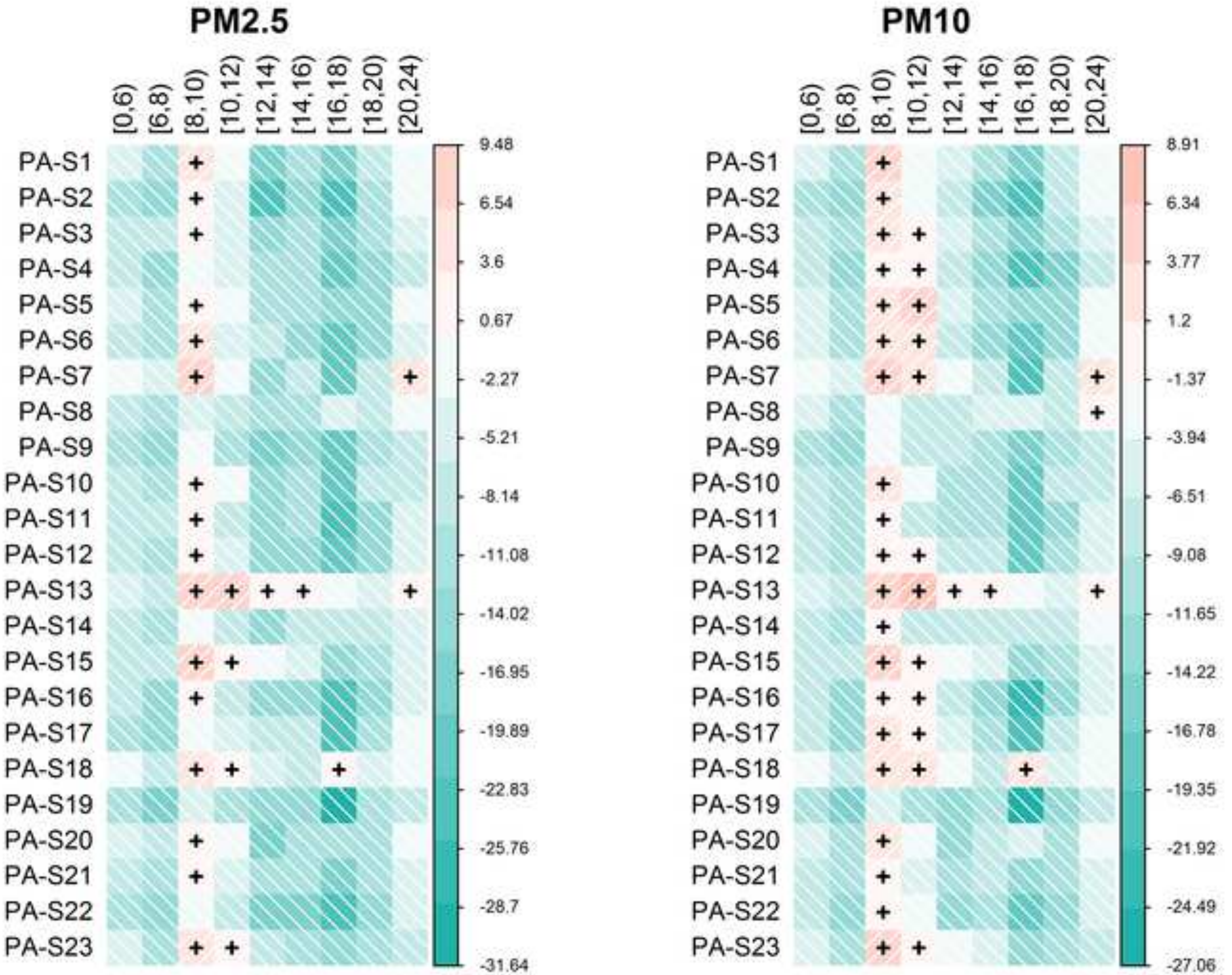

Click here to access/download;Figure;Fig3.tif

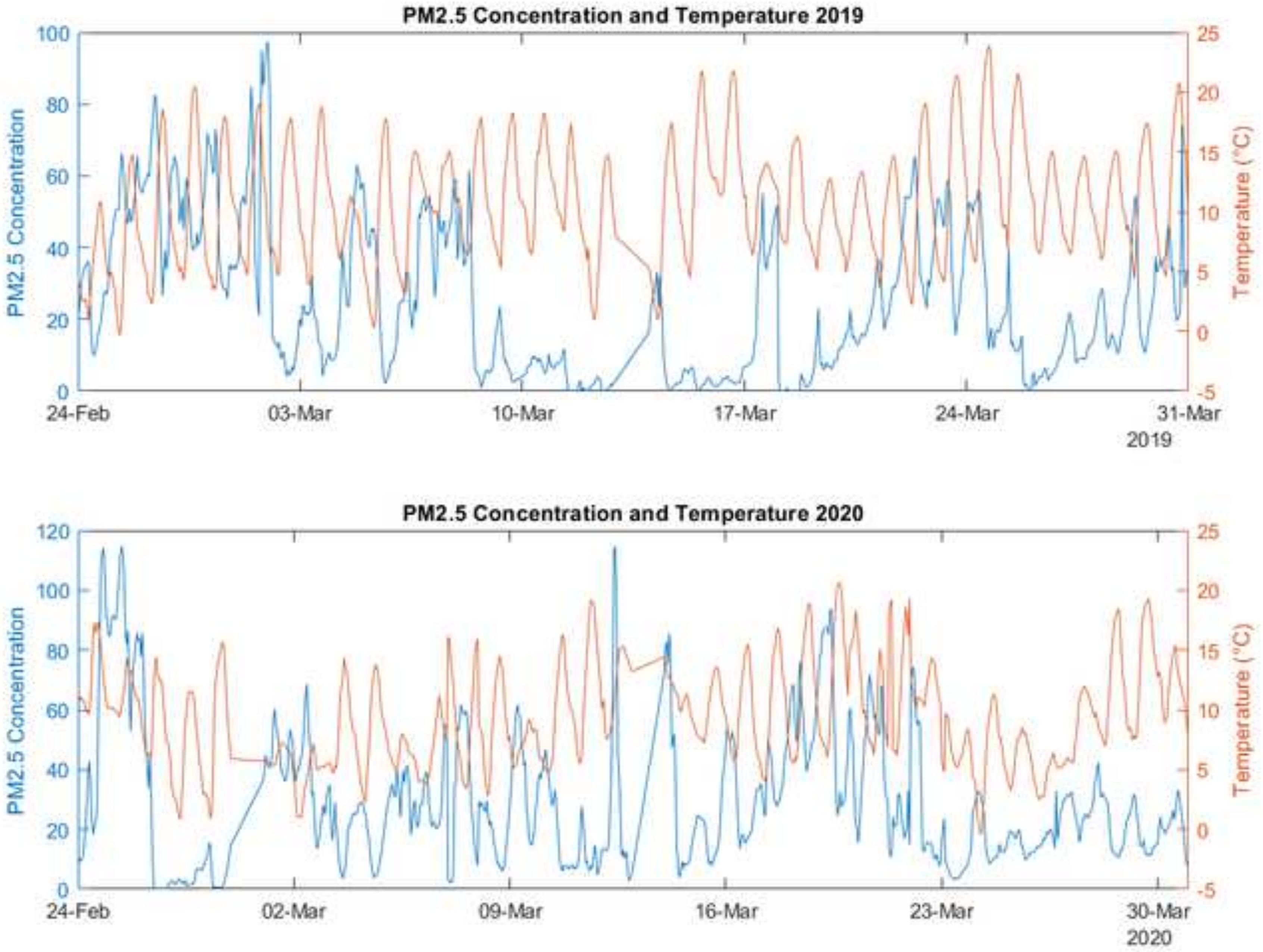

Click here to access/download;Figure;Fig1.tiff

Fig2.JPG

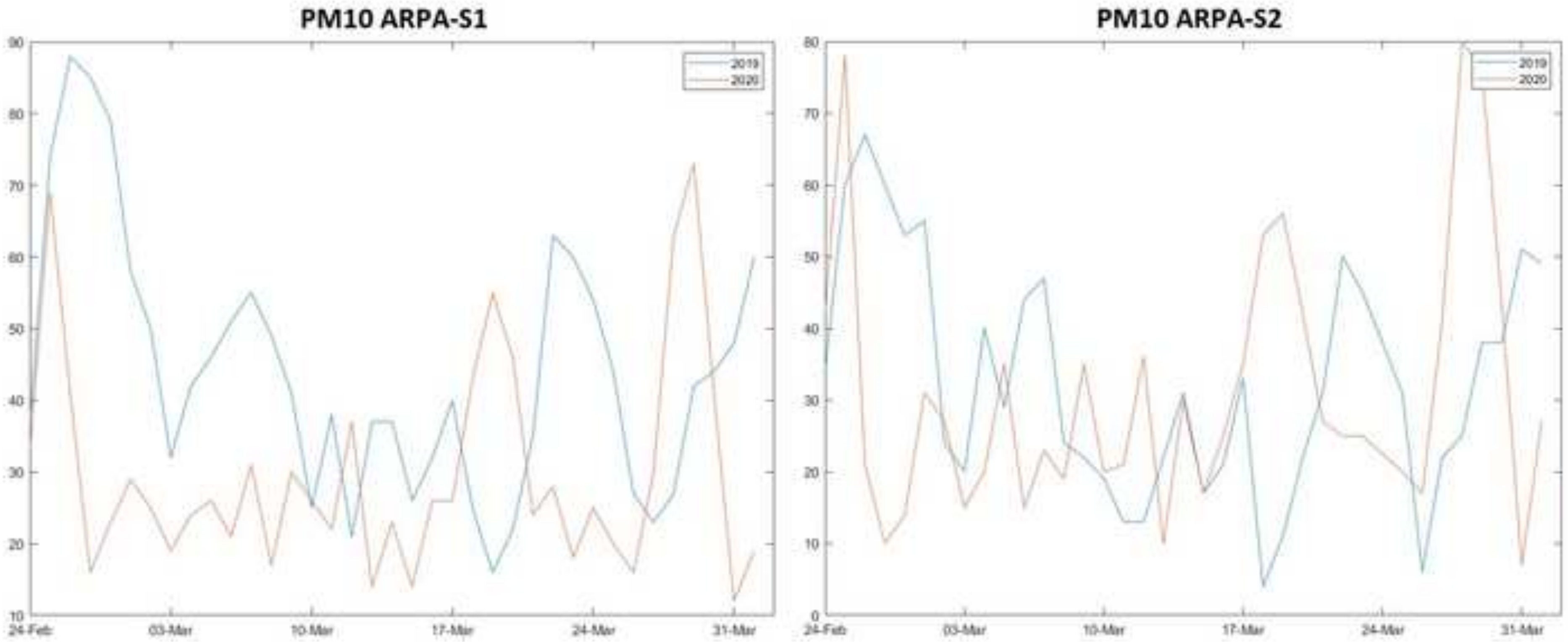

Click here to access/download;Figure;Fig2.tif

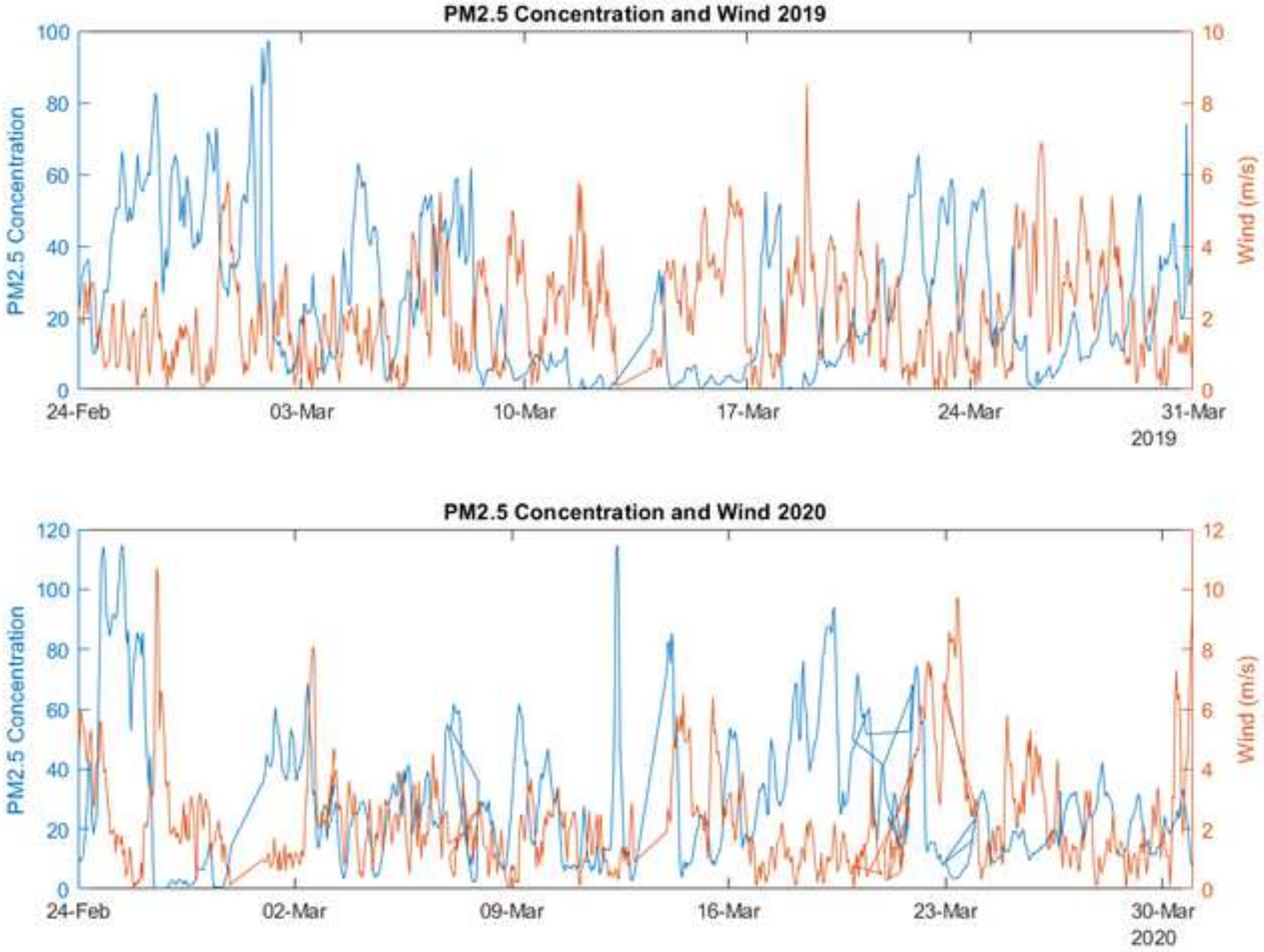

Click here to access/download;Figure;Fig9.tiff

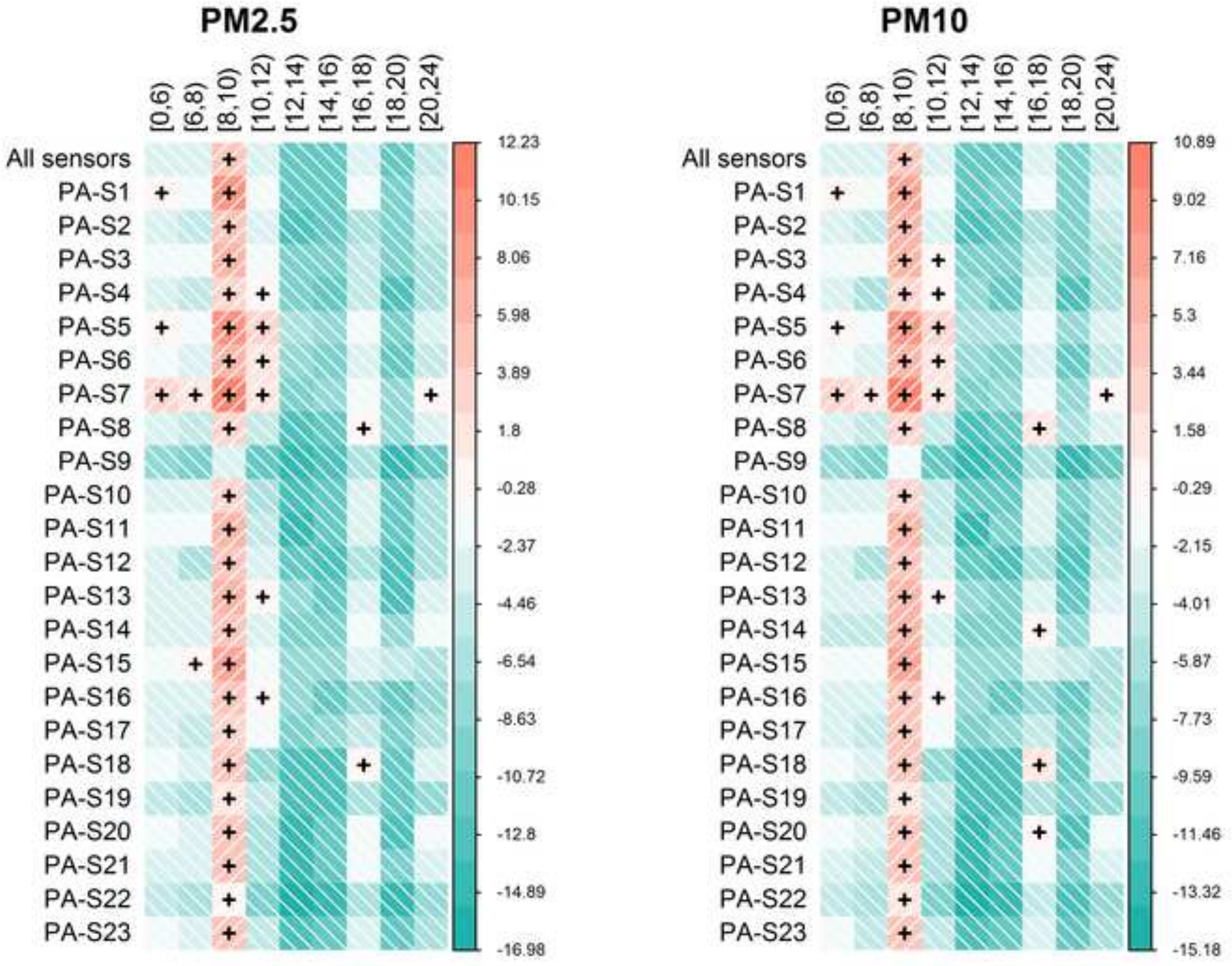

Click here to access/download;Figure;Fig4.tiff

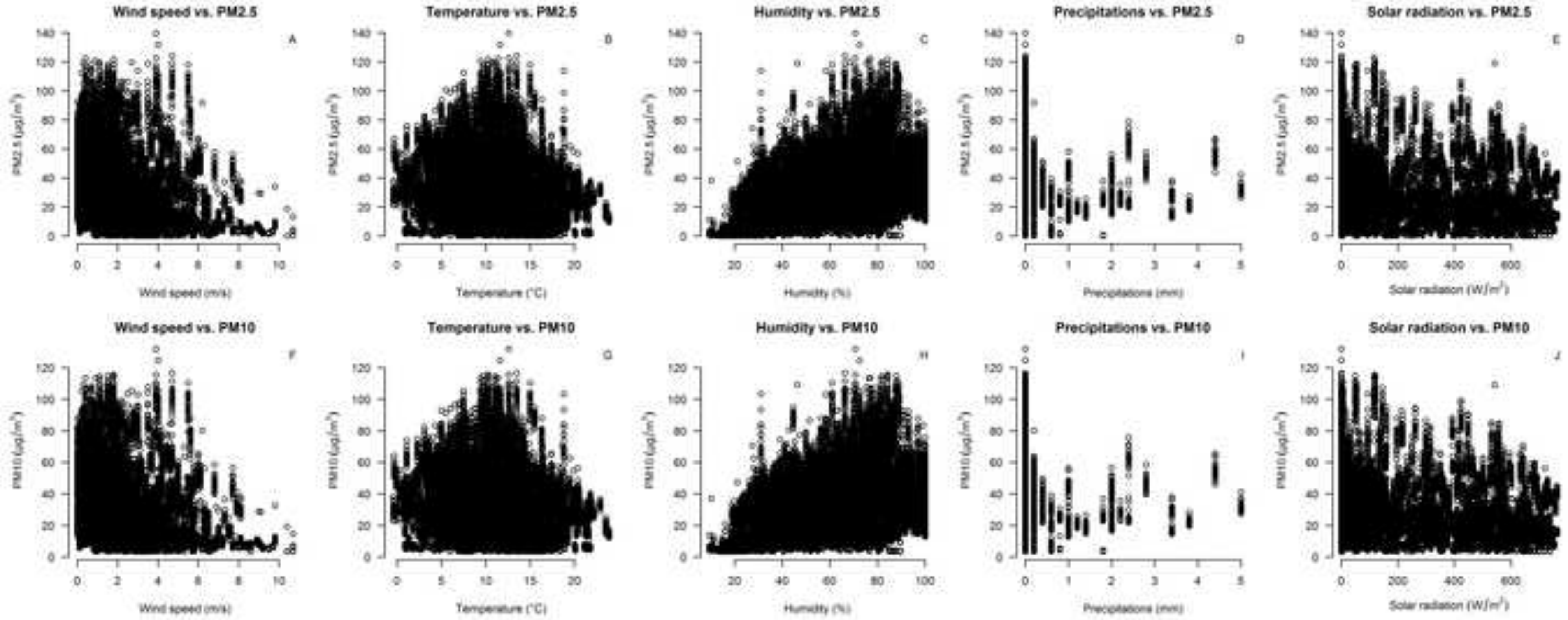

Click here to access/download;Figure;Fig5.tiff

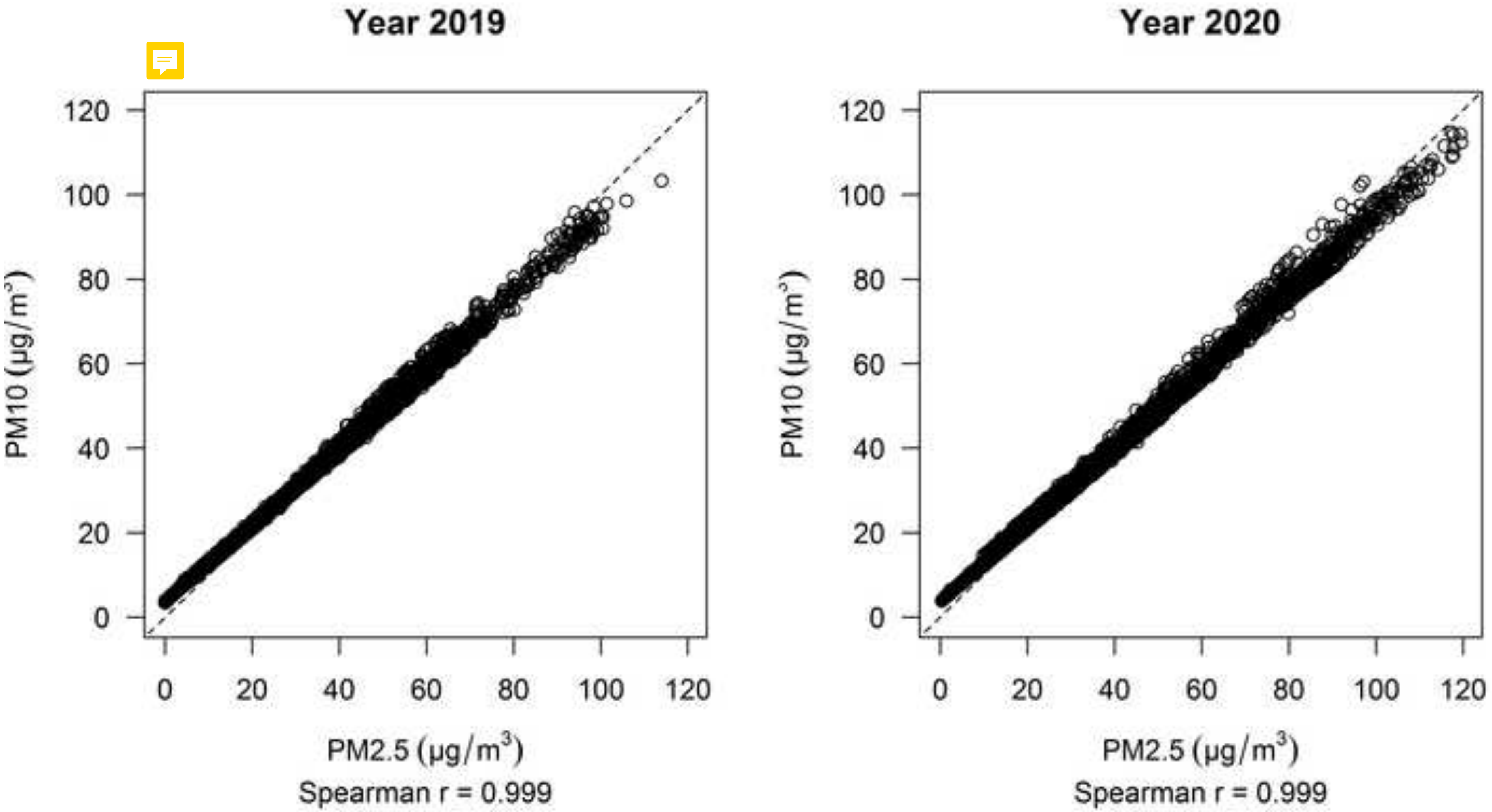

Click here to access/download;Figure;Fig6.tiff

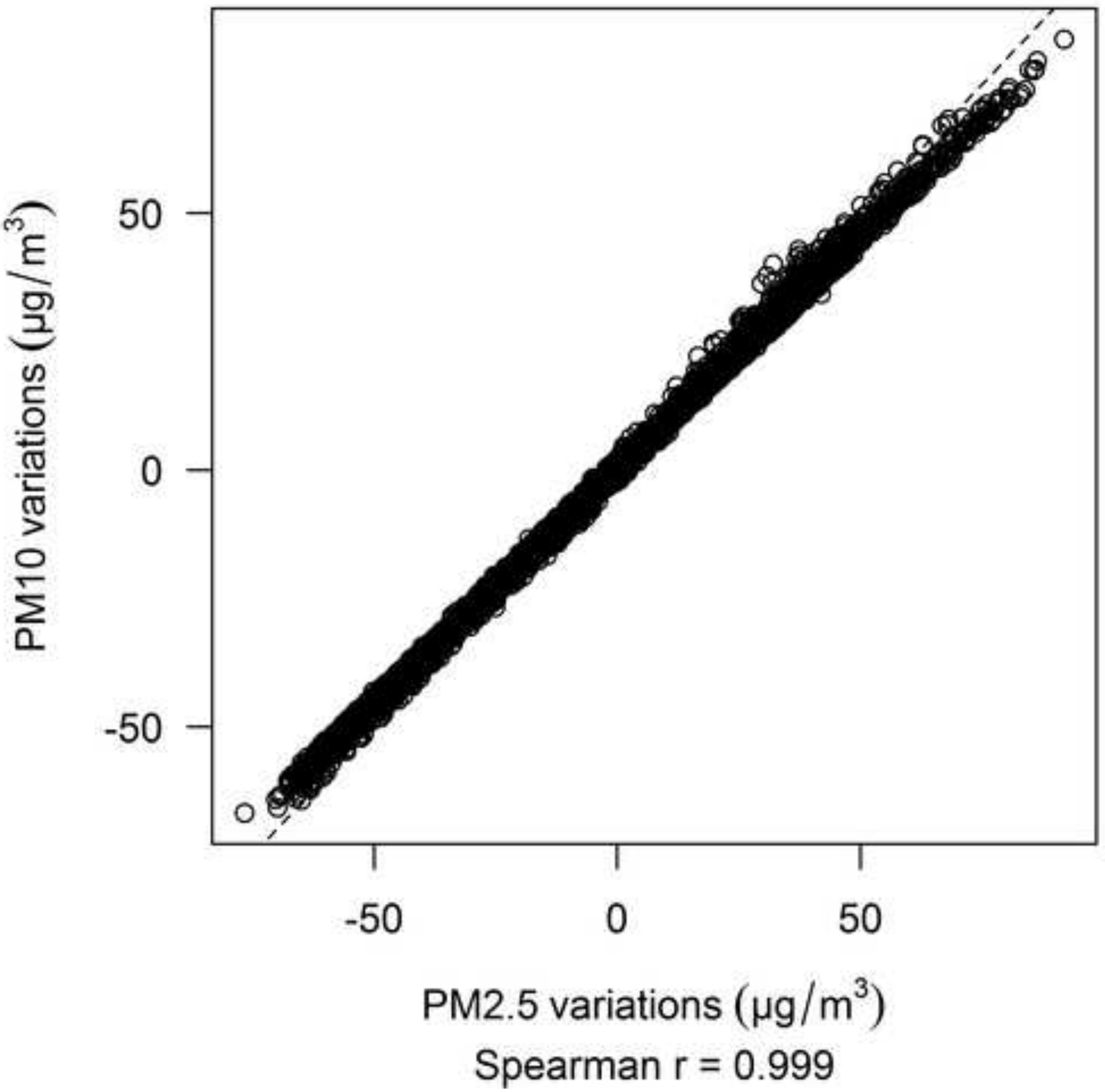

Click here to access/download;Figure;Fig7.tiff

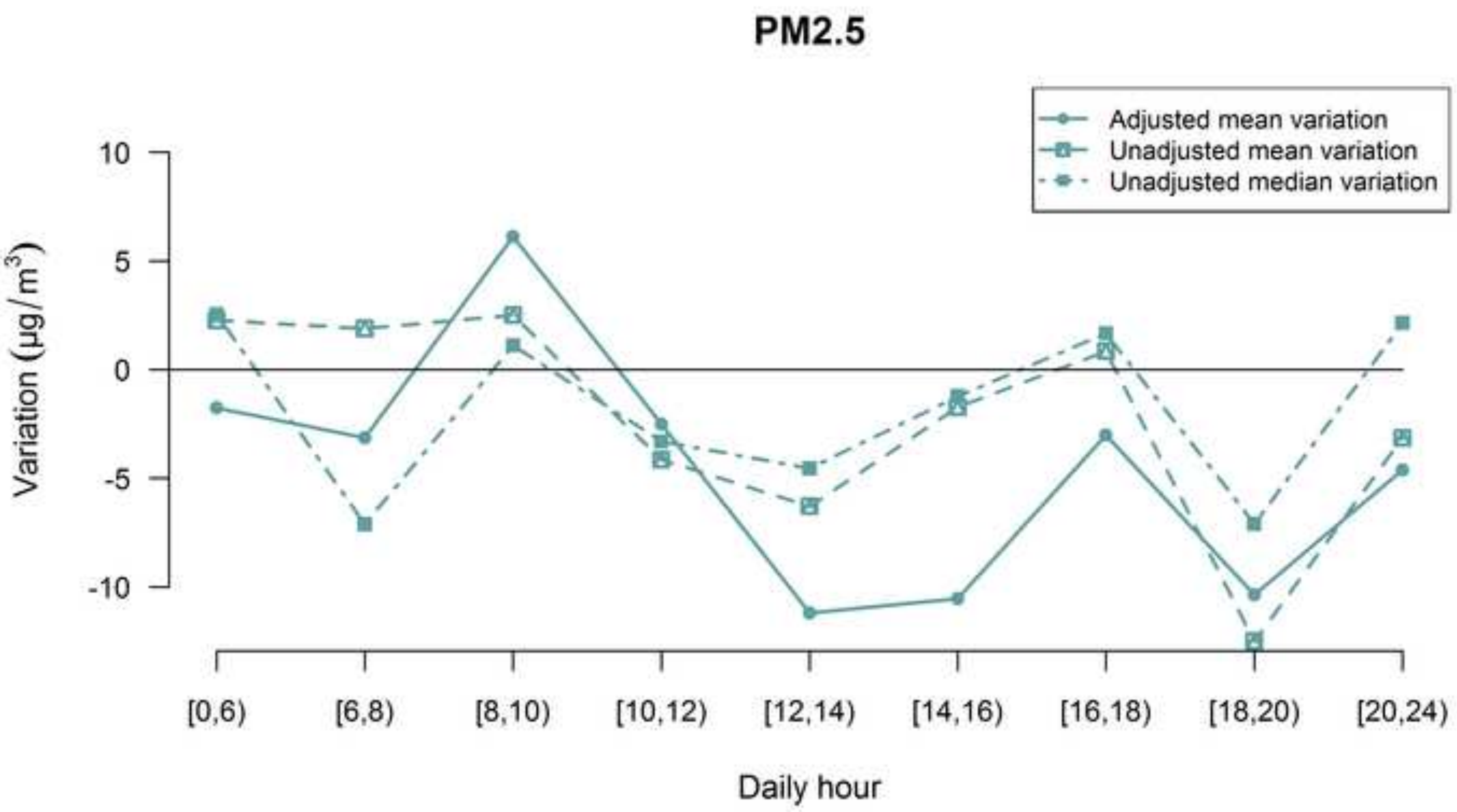

Click here to access/download;Figure;Fig8.tiff

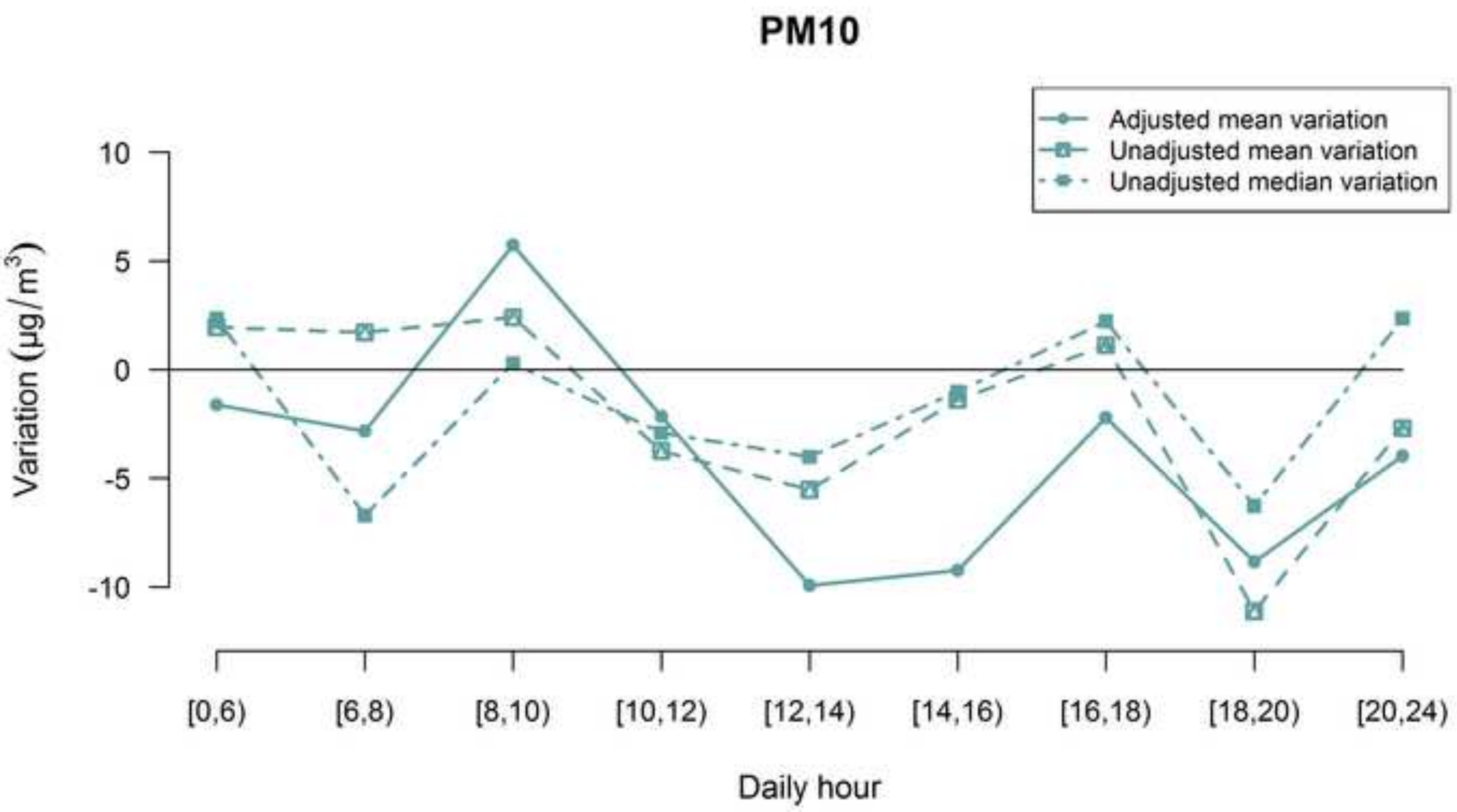

Click here to access/download
Supporting Information
Support_Information.docx

Impact of COVID-19 Lockdown on PM Concentrations in an Italian Northern City: a year-by-year Assessment

D.Pala[1*], V.Casella[2], C.Larizza[1], A.Malovini[3], R.Bellazzi[1]

[1]Department of Electrical, Computer and Biomedical Engineering, University of Pavia, Pavia, Italy.

[2]Department of Civil Engineering and Architecture, University of Pavia, Pavia, Italy.

[3]Istituti Clinici Scientifici Maugeri SpA SB IRCCS, Pavia, Italy.

*Corresponding author

E-mail: daniele.pala@unipv.it

Formatted: English (United Kingdom)

Abstract

In the last century, the increase in traffic, human activities and industrial production have led to a diffuse presence of air pollution, which causes an increase of risk of several health conditions such as respiratory diseases. In Europe, air pollution is a serious concern that affects several areas, one of the worst ones being northern Italy, and in particular the Po Valley, an area characterized by low air quality due to a combination of high population density, industrial activity, geographical factors and weather conditions. Public health authorities and local administrations are aware of this problem, and periodically intervene with temporary traffic limitations and other regulations, often insufficient to solve the problem. In February 2020, this area was the first in Europe to be severely hit by the SARS-CoV-2 virus causing the COVID-19 disease, to which the Italian government reacted with the establishment of a drastic lockdown. This situation created the condition to study how significant is the impact of car traffic and industrial activity on the pollution in the area, as these factors were strongly reduced during the lockdown. Differently from other areas in the world, a drastic decrease in pollution measured in terms of particulate matter (PM) was not observed in the Po Valley during the lockdown, suggesting that several external factors can play a role in determining the severity of pollution. In this study, we report the case study of the city of Pavia, where data coming from 23 air quality sensors were analyzed to compare the levels measured during the lockdown with the ones coming from the same period in 2019. Our results show that, on a global scale, there was ~~no~~ a statistically significant reduction in terms of PM levels taking into account ~~all~~ meteorological variables that can influence pollution such as wind, temperature, humidity, rain and solar radiation. ~~However, d~~Differences can be noticed analyzing daily pollution trends too, as – compared to 2019 – during 2020 pollution was higher ~~in the night and lower~~ in the morning and lower in the ~~evening rush hours~~remaining hours~~, taking into account the influence of meteorological factors such as wind and temperature.~~.

Introduction

In the last decades, air pollution has become a major threat to health and climate in several countries of the world, most of them being urban or highly populated areas. Scientific investigations showed that exposure to air pollution can lead to an increased risk of developing or exacerbating several diseases,

in particular respiratory and cardiovascular ones [1]. The prevalence of diseases such as asthma, type 2 diabetes and cardiovascular disorders is also increasing in most of the world [2–4]. While public health organizations, together with non-profit associations and governments are struggling to apply “green solutions” to contain the increase in pollution levels, a sudden reduction of air pollutants concentration was observed in many countries between the end of the year 2019 and the year 2020. During this period, most of the productive activities throughout the world had to come to an unexpected stop due to the pandemic caused by the new SARS-CoV-2 virus, and the related diseases, COVID-19. This virus generated most likely in a market in Wuhan, China, and quickly spread causing a high number of intense flu-like syndromes and cases of atypical pneumonia. Compared to influenza viruses, this unknown pathogen showed abnormally high contagious strength, with higher hospitalization and death risk [5]. Despite the initial efforts to contain the disease, the virus spread in several Asian countries outside of China, and at the end of February 2020 the first European case unrelated to the Asian outbreak was found in northern Italy, in the city of Codogno in Lombardy. In the following weeks, Italy suffered a tremendous increase in terms of COVID-19 cases, forcing the government to take drastic actions. Thus, Italy was the first western country to apply severe measures and to apply a general lockdown, with most of the population confined at home and a shutdown of all nonessential productive activities and services.

The first area where schools and factories have been closed included the most densely populated and industrialized regions of northern Italy, located in a large area named Po Valley. Among other aspects, this area is known to be among the most polluted in Europe [6], since several air pollutants, including particulate matter, often rise to dangerous levels. This phenomenon is due to an unfortunate combination of factors such as high population density, intense industrial activity and geographic position, as the valley is surrounded on three sides by mountains. Starting from China in December 2019, a reduction of human related pollution was observed in all areas where the lockdown was applied by governments. In several parts of the world air pollution dropped to the lowest levels in decades [7,8]; this happened particularly in Asian countries such as China and India. In Italy, although a similar effect has been observed, the reduction in terms of concentration was not evident for all pollutants. A significant decrease in nitrogen dioxide and sulfur dioxide was observed during the first period, whereas no drop in particulate matter (PM10 and PM2.5) was recorded [9]. Peculiar meteorological conditions such as high concentration of sea salt and Saharan sand brought by Eastern winds at the beginning of the lockdown and a coincidental increase in the wind speed for several days could have contributed to this phenomenon [10]. Meteorological factors are known to play a considerable role in modulating particulate matter and other pollutants concentration: wind and precipitations tend to disperse them, while lower or higher temperatures can create favorable conditions for higher concentrations. The presence of confounders makes it difficult to assess the real impact of traffic, industrial activities and house heating on the pollution levels, especially because they can potentially interact in complex ways. The sudden lockdown condition in which Italy entered between February and March 2020 allowed to analyze how vehicular traffic and factories activities impact on air pollution during the daily life. Although traffic was limited to essential transportations and factories not producing essential goods were closed during this period, results from studies resulted controversial due to the extreme variability in terms of weather condition typically characterizing the month of March [11]. Air pollution is a typical problem in the Po Valley during winter months [12], when cold dense air tends to stagnate in the lower layers of the atmosphere. The phenomenon is less common in the warm season as air is less dense and local breezes are more frequent.

In this paper we present the results from a study performed in Pavia, located in the Po valley in Italy, where 45 air quality sensors have been deployed in the urban area in the context of the European project PULSE (Participatory Urban Living for Sustainable Environments [https://www.project-pulse.eu/]), allowing to create a dense network of pollutants measurements.

The primary objective of the analysis was to assess the impact of the lockdown on the urban PM pollution. To this aim we analyzed ~~PM10~~ PM2.5 and PM~~2.5~~10 data coming from the available sensors

with high spatial and temporal resolution and compared measurements performed from the end of February to the beginning of April of 2020 with those deriving from the same period in 2019. Analyses consisted of several steps: we first analyzed data from two official monitoring stations in Pavia, then we investigated the effect of meteorological conditions (wind and temperature) on the measurements and finally we estimated the adjusted mean variations in terms of PM2.5 and PM10 between years 2019 and 2020 at a whole data level and at a single sensor level stratified by daily hours.

Materials and Methods

The PULSE Project

New public health initiatives are emerging to face the new challenges brought by the increase of population in the urban areas and the global changes in air pollution and lifestyle. Among these, the PULSE project was funded by the European Commission to perform heath research in big cities across Europe, Asia and the U.S.A. through a collaboration between universities, research centers and city councils. The project started during the fall of 2016 and lasted until April 2020 with clinical focus on the link between air quality and asthma, physical inactivity and type 2 diabetes and on the influence of exposures on health and wellbeing in general. A complex multi technological platform was created in the context of the PULSE project and tested in seven cities: Barcelona (Spain), Birmingham (United Kingdom), Keelung (Taiwan), New York (U.S.A.), Paris (France), Pavia (Italy) and Singapore. This platform is based on a sophisticated data exchange infrastructure that involves data coming from different sources, including satellite data, open data and dedicated sensing technology for air quality.

The sensors network in Pavia

The city of Pavia, Italy, was one of the test sites of the PULSE system. Although Pavia is a rather small city (about 72,000 inhabitants), its university and its peculiar position in the geographical area of the Po Valley makes it a suitable location to carry on research and innovation projects to study air quality and personal exposure to pollutants, being air pollution and its related health risk a frequent problem ~~affecting~~ that affects this area during winter months.

One of the key innovation aspects of PULSE is the use of data with high spatial and temporal resolution. The increase in terms of resolution can be beneficial to study phenomena of public health interest at a neighborhood level, thus taking into account social and health disparities that often characterize large urban environments, as highlighted by studies we performed in the context of the project [13,14]. Despite this necessity, urban data is rarely collected with a sufficient spatial granularity, due to the high costs and difficulties of the process. Taking air quality as an example, pollution is usually measured by high quality monitoring stations deployed in the cities by official agencies or state departments. Being these instruments expensive and requiring constant maintenance they are usually deployed in small numbers. As an example, the entire city of New York has only 13 monitoring stations while Pavia has 2 stations over an area of about 65 km^2. For this reason, low-cost and portable sensing technologies are becoming common [15] but generating controversial results: while the frequency and easiness of measurements increase with low-cost technology, the quality of the data collected is often limited.

A total number of 45 low-cost PurpleAir sensors [16] were purchased and installed throughout Pavia in agreement with the municipality and with the collaboration of a few private citizens within the PULSE project. These sensors are small outdoor devices that measure three types of pollutants, i.e. PM1, PM2.5 and PM10, and ~~T~~temperature and ~~H~~humidity as meteorological data. These instruments~~y~~ are easy to use as they can be ~~drilled~~installed on private apartments' ~~people's~~ balcon~~y~~ies and require only a Wi-Fi connection and an electrical outlet. The ~~sensor~~ network created ~~with~~by these sensors was added to already existing official monitoring stations, property of *ARPA Lombardia*~~. ARPA Lombardia~~

(Environmental Protection Regional Agency of the region of Lombardy)~~ is~~, a public agency that ~~has the~~ aims ~~of~~at measuring ~~and treating~~ environmental data in the Lombardy region in Italy – where Pavia is located~~of the region the city of Pavia is located in~~. Through numerous sensors scattered throughout the region, ARPA collects a large quantity of data about air ~~pollution~~quality, meteorology, agriculture, sole status, etc.

In Pavia, there are two official ARPA air quality monitoring stations, they are high-quality fixed stations that measure several pollutants (NO_X, SO_2, CO, O_3, PM10 and PM2.5) at regular time intervals. These sensors are calibrated continuously using commercially available instruments that can be used as reference according to Italian or European laws [17] and therefore can be considered very reliable. Weather parameters are collected as well, and data is freely available upon request on a dedicated portal. An analysis has been performed to evaluate whether measurements performed by the Purple Air sensors were different from the ones performed by one of the official monitoring stations~~,~~ belonging to the local environmental agency~~ (Agenzia Regionale per la Protezione Ambientale, ARPA)~~ ARPA. It was observed that, despite a small offset, the Purple Air sensors' measurements were highly correlated with the official ARPA ones (correlation > 0.9). Thanks to the large number of available devices, a dense sensors network was created, allowing to derive high-quality homogeneous maps through interpolation and to use them in the monitoring of pollution levels and personalized risk for the citizens that move around the city. Besides defining a spatially dense network, these sensors allow also for high temporal granularity in the measurements, by quantifying PM1, PM2.5, PM10 every 80 seconds. The geographical position of the Purple Air sensors located in Pavia can be visualized on the real time map available on the Purple Air website, showing the latest sensors' measurements [18].

~~**Fig 1** shows PurpleAir sensors location in the city of Pavia.~~

~~**Fig 1**. Map of PurpleAir sensors location in the city of Pavia as of May 2020, showing the average daily Air Quality Index on a selected day.~~

Analyzed data

Two main data sources have been used in this study: air quality measurements coming from the Purple Air sensors and weather data (wind speed, ~~ and~~ temperature, humidity, precipitations and solar radiation) acquired from the ARPA official database. In particular, used sensors measure all kinds of particulate matter (PM1, PM2.5 and PM10), but only ~~PM10~~ PM2.5 and PM~~2.5~~10 have been considered in our analysis, since they are well-agreed indexes of air pollution. Even though the Purple Air sensors themselves measure weather data (temperature and humidity) ~~(quali???)~~, these data were not of interest in the PULSE project and therefore the accuracy of weather measurements was not determined. For this reason, we decided to use the official ARPA sensors ~~for~~to measure meteorological variables included in the study. Being Pavia a small city, it is not expected to observe significant meteorological differences ~~in~~across different areas of the city.

We considered hourly pollution measurements data during two periods of time: from February 24th, 2020 (00:00 CET) to April 2nd, 2020 (24:00, CEST) and the same period during year 2019. During 2020 the time period considered started in correspondence of the days after the identification of the first COVID-19 cases in northern Italy, with the consequent closing of schools and of most of the commercial activities and the deriving reduction in terms of urban mobility (the so-called "lockdown"). The same temporal period during year 2019 has been considered as reference, to estimate PM2.5 and PM10 variations between the two years. Meteorological data (measurements of hourly average wind speed, maximum wind gusts, ~~ and~~ air temperature, humidity, precipitations and solar radiation) were collected with the same hourly temporal granularity as PM. Only sensors with available measures for both 2019 and 2020 were included in the analysis.

~~Data quality control~~

Formatted: Subscript

Formatted: Subscript

Formatted: Subscript

Formatted: Font: Bold

Formatted: Font: Not Bold

~~Scatterplots and boxplots by sensor were generated to visually inspect the correlation between PM.2.5 and PM10 concentration (µg/m³) and to identify potential outlier measurements.~~

Data calibration

Data measured by Purple Air sensors were calibrated using the official ARPA monitoring stations measurements, using data of a single sensor located close to one of the ARPA stations (PurpleAir sensor 3, PA-S3). Linear regression was used to calibrate the sensor data according to the following model:

$$y = m \cdot x + b$$

Where y indicates data coming from the Purple Air sensor, x those coming from the ARPA monitoring station, and m and b are the regression coefficients, representing the slope and the intercept respectively. Using measurements with the same temporal granularity (measurements were aggregated by day as the ARPA data was available only with this temporal granularity), the values of m and were estimated for PM2.5 and PM10 independently. Data from the considered sensor and from the ARPA station showed evidence of strong correlation (r = 0.83 and r = 0.80 for PM2.5 and PM10 respectively). Once the parameters were estimated, all sensors' measurements were corrected by the inverse formula:

$$\hat{x} = \frac{(y - b)}{m}$$

Specifically, by comparing the PM2.5 measurements between the Purple Air and the ARPA sensors we obtained m = 1.5755 and b = -5.7709, while m = 1.1851 and b = -16.1240 when comparing PM10 values. Thus, by defining PM2.5' and PM10' as the crude values measured by our sensors, the corresponding scaled PM2.5 and PM10 values have been calculated as follows:

- PM2.5 = (PM2.5' + 5.7709) / 1.5755
- PM10 = (PM10' + 16.1240) / 1.1851

Scaled PM2.5 and PM10 measurements were then used for the analyses reported in the next sections.

Considering that the Purple Air sensors have all the same hardware, it was enough to locate only one sensor close to the ARPA one to estimate the correction to be made, and then apply it to all the other Purple Air sensors, rather than co-locate all sensors close to the ARPA ones. Furthermore, an ~~small~~ internal study ~~that was~~ performed at the University of Pavia locating seven Purple Air sensors in the same place showed ~~that~~ evidence of strong correlation between measures performed by co-located ~~Purple Air~~ sensors~~ measure PMs with high correlation~~. Results ~~of~~are reported~~ this comparison are visible~~ in **Supplementary Figures 1-4**, showing the correlation matrices and the range of measured values for each sensor on a selected day (July 24th 2019). Considering that Pavia is a small city with no significant climatic differences ~~within~~across different zones, we assumed that ~~a~~ possible changes in performance over time ~~does~~should not affect some sensors more than others.

Data pre-processing

Only data from 24 out of the 45 Purple Air sensors available in Pavia were analyzed since some of these were not active ~~for~~during both years 2019 and 2020. Scatterplots and boxplots by sensor were generated to visually inspect the correlation between PM.2.5 and PM10 concentration (µg/m³) and to identify potential outlier measurements.

Visual inspection of boxplots allowed identifying and removing 4 outlier values corresponding to PM10 and PM2.5 > 250, for a total number of 39,926 measurements passing quality control.

General pollution trend over the years

Formatted: Font: Bold
Formatted: Font: Bold
Formatted: Superscript
Formatted: Not Highlight
Formatted: Not Highlight
Formatted: Not Highlight
Formatted: Not Highlight
Formatted: Not Highlight
Formatted: Not Highlight
Formatted: Not Highlight
Formatted: Not Highlight

Before comparing 2019 and 2020 data ~~with a~~by statistical ~~analysis~~methods, it is useful to check for the presence of potential ~~observe whether there is an~~ increasing or reducing trends in ~~the~~ PM levels in the study area. The continuous technological improvements in vehicular traffic and energetic efficiency are presumably leading to a slow decrease of average pollution levels in Europe, and this could be a confounding element in the evaluation of the lockdown effects using data from the previous year, since an observed reduction could be due to the general trend instead of the lockdown itself. According to data in literature and past articles, there is some evidence of a PM10 and PM2.5 reduction trend in all the area in the last years [19] although with notable fluctuations. Looking at an article published by the Italian National Environmental Protection System [20], it can be noticed that the reduction trend appears less evident after 2018, with even a little increase, probably not significant, in the PM10 concentrations in 2020. The article itself states that the meteorological variability could have played an important role in the measurements' variations, as in 2019 and 2020 temperatures were generally higher and precipitations lower than the previous years. Therefore, it is reasonable to assume that the general trend ~~of~~characterizing the last years probably did not significantly influence the difference in PM2.5 and PM10 concentrations between 2019 and 2020.

Statistical and data mining methods

The Wilcoxon Mann Whitney test was applied to compare numeric variables distribution between groups. Multivariate regression trees were fitted to identify informative wind speed ~~and temperature~~ cut off values able to discriminate subpopulations of PM2.5 and PM10 measurement~~s~~ ~~characterized by different average PM2.5 and PM10~~ values. The one sample t-test was applied to test the null hypothesis that the mean variation in terms of PM pollutants between years was zero. The Spearman correlation coefficient *r* and corresponding 95% confidence interval (95% CI) were computed to estimate the correlation between numeric variables~~: 95% CIs were computed~~ by a bootstrap approach based on 1,000 replicates. Linear mixed model regression was used to estimate the adjusted mean variation in terms of PM2.5 and PM10 between years 2019 and 2020, using data matched between years by measurement month, day and hour. Year (binary: 2019 coded "0", 2020 coded "1"), working day (binary: week end coded "0", working day coded "1"), wind (numeric continuous, expressed as m/s), temperature (numeric continuous, expressed as °C), humidity (numeric continuous, expressed as %), precipitations (numeric continuous, expressed as mm), solar radiation (numeric continuous, expressed as W/m^2) and sensors elevation upon the sea level (continuous, expressed as meters) were included as fixed effect terms, while a single variable resuming the sensors ID/month/day/hour of measurement was used as random effect grouping variable. The interaction between year and daily hour was also included in order to estimate the adjusted mean variation in terms of pollutants concentration by daily hours interval. Analyses were performed at a whole data level and then repeated by sensor.

An alternative approach consisting of a modification of the method proposed by Venter et al. [~~ref~~21] has been also applied to estimate the potential impact of the lockdown on pollutants concentration during year 2020. The method is described in the detail in the **Supplementary Material** section.

Analyses were performed by the R statistical software tool version 3.6.1 (www.r-project.org). Regression trees were fitted by the *rpart* fuction implemented in the *rpart* package. Spearman ~~regression~~ coefficient~~s~~ and corresponding 95% CI were computed by the *spearman.ci* function implemented in the R package called *RVAideMemoire*. Linear mixed model analysis was performed by the *lmer* function implemented in the R package called *lme4*. MATLAB (R2020b Version) was used for sensors' calibration, analyses regarding the official ARPA data and the creation of visual representations of the PMs trends in the two years.

Results

Analysis of the Official ARPA Data

Formatted: Font color: Auto

Formatted: Highlight

Formatted: Font: Bold

Formatted: Font: Not Bold

Formatted: Font: (Default) Times New Roman, Font color: Black

ARPA owns two sensors in the urban area of Pavia: the first one (ARPA-S1) is located in a square very close to the city center and along one of the most important traffic arteries of the city, characterized by high traffic during the rush hours. The second one (ARPA-S2) is located in a residential area, densely populated but less prone to traffic. Unfortunately, ARPA historical data are available with a daily temporal granularity for PM10 only. As a matter of fact, ARPA-S1 open data does not contain PM2.5 measurements and ARPA-S2 open data are characterized by an extremely high missing data fraction in the considered period.

When comparing measurements distribution between years it was possible to observe different behaviors of the two sensors, as ARPA-S1 showed a significant decrease in the PM10 levels during the year 2020 (median decreasing from 42 μg/m^3 to 26 μg/m^3, p-value = 0.0016), whereas ARPA-S2 measurements distribution did not show significant variations (p-value = 0.4571). ~~We used a Wilcoxon Mann Whitney test since the variables were not normally distributed.~~ The~~is~~ difference between the two sensors is visible also in **Fig** 1~~2~~, where PM10 measurements of both sensors in 2019 and 2020 are shown. This analysis was performed on daily averaged data for each sensor, the only data available from the ARPA dedicated portal.

These differences are presumably due to the fact that pollution levels are highly dependent on meteorology, and spring months like March ~~are the most meteorologically unstable months in which~~are often characterized by very quick weather changes (in terms of wind, humidity, pressure, temperature, etc.)~~, can vary very quickly and within small spaces~~. This enlightens the necessity to take meteorological factors into account when comparing two ~~different~~time periods.

Fig 1 ~~– Daily trend of~~ PM10 daily trends during 2019 and 2020 measured by the two ARPA monitoring stations.

Besides PM2.5 and PM10, ~~Looking at the situation related to other~~when focusing on other kinds of pollutants measured by ARPA sensors (CO, NO_2, SO_2 and SO) it has been possible to observe ~~rather than PMs, some interesting results can be seen. Using the same ARPA data, we applied the same Wilcoxon Mann Whitney statistical test on CO, NO_2, SO_2 and SO data, since the data were not distributed normally. The results are reported in Table 1,~~ a statistically significant reduction in terms of all pollutants except for CO during 2020 compared to 2019 ($p < 0.0001$) (**Table 1**)~~and show that all pollutants except CO had a significant decrease in 2020~~.

**Pollutant**	**Median 2019 (Q1:Q3)**	**Median 2020 (Q1:Q3)**	**p-value**
CO	0.6 (0.6:0.7)	0.7 (0.5:0.8)	0.1295
NO_2	34.0 (20.6:47.9)	20.9 (13.3:31.7)	~~$4.6 \cdot 10^{-52}$~~<0.0001
SO_2	5.2 (4.3:6.1)	1.8 (1.4:2.3)	<0.0001~~$3.3 \cdot 10^{-246}$~~
SO	42.7 (27.7:64.5)	28.2 (18.0:42.5)	<0.0001~~$3.4 \cdot 10^{-46}$~~

Table 1. Results of the Wilcoxon Mann Whitney test on ARPA data. The table reports the median values of the pollutants in the considered periods in 2019 and 2020, together with their 25th and 75th percentiles, and the tests' p-value. Apart from CO, all pollutants had a significant decrease in 2020 compared to 2019, even neglecting the effect of meteorological variables.

~~**Fig 2** – Daily trend of PM10 during 2019 and 2020 measured by the two ARPA monitoring stations.~~

Commented [MA1]: Manca la didascalia, riportare

~~Data pre-processing~~

~~Only data from 24 out of the 45 PurpleAir sensors available in Pavia were analyzed since some of these were not active for both years 2019 and 2020. Visual inspection of boxplots allowed identifying and removing 4 outlier values corresponding to PM10 and PM2.5 > 250, for a total number of 40,043~~39,926 ~~measurements passing quality control. Data were then calibrated based on the ARPA measurements as described in the **Material and Methods** section.~~

Effect of potential confounders on pollutants concentration

~~Visual inspection of sensors data boxplots allowed the identification and removal of 4 outlier values corresponding to PM10 and PM2.5 > 250, and~~After outliers removal ~~–~~a total number of 39,926 measurements passed quality control. An exploratory analysis has been performed to inspect how the pollution trends have changed during the different time periods and how weather conditions ~~(wind speed and temperature)~~ influenced this change. Looking at the absolute quantities reported in **Fig 2~~3~~**, showing scaled data from the sensor PA-S3 (the same used for data calibration), a very irregular trend in pollution concentration can be observed during both years, suggesting that external factors can influence these measurements.

Fig 2~~3~~ – PM2.5 concentration and wind speed in the two considered periods in 2019 (upper plot) and 2020 (lower plot).

Looking at the two plots, it is possible to observe that high wind speed peaks correspond generally to lower concentrations of PM2.5. The same trend has been observed for PM10 (**Supplementary Figure 5~~1~~**).

Similarly, the effect of temperature on PM concentration (scaled values) has been inspected. Plots in **Fig 3~~4~~** and **Supplementary Figure 6~~2~~** show an apparently milder relationship between temperature and pollutants concentration.

Fig 3~~4~~ – PM2.5 concentration and temperature in the two considered periods in 2019 (upper plot) and 2020 (lower plot).

The relationship between wind speed (m/s), temperature (°C), humidity (%), precipitations (mm), solar radiation (W/m^2) and pollutants' concentration using scaled data pooled from both 2019 and 2020 was assessed by visual inspection of the scatterplots reported in **Fig 4~~5.~~**

Formatted: Font: Bold

Formatted: Font: Bold

Formatted: Font: Bold

Formatted: Font: Bold

Formatted: Left, Space After: 8 pt

Formatted: Left, Space After: 8 pt

Formatted: Font: Bold

Formatted: Font: Bold

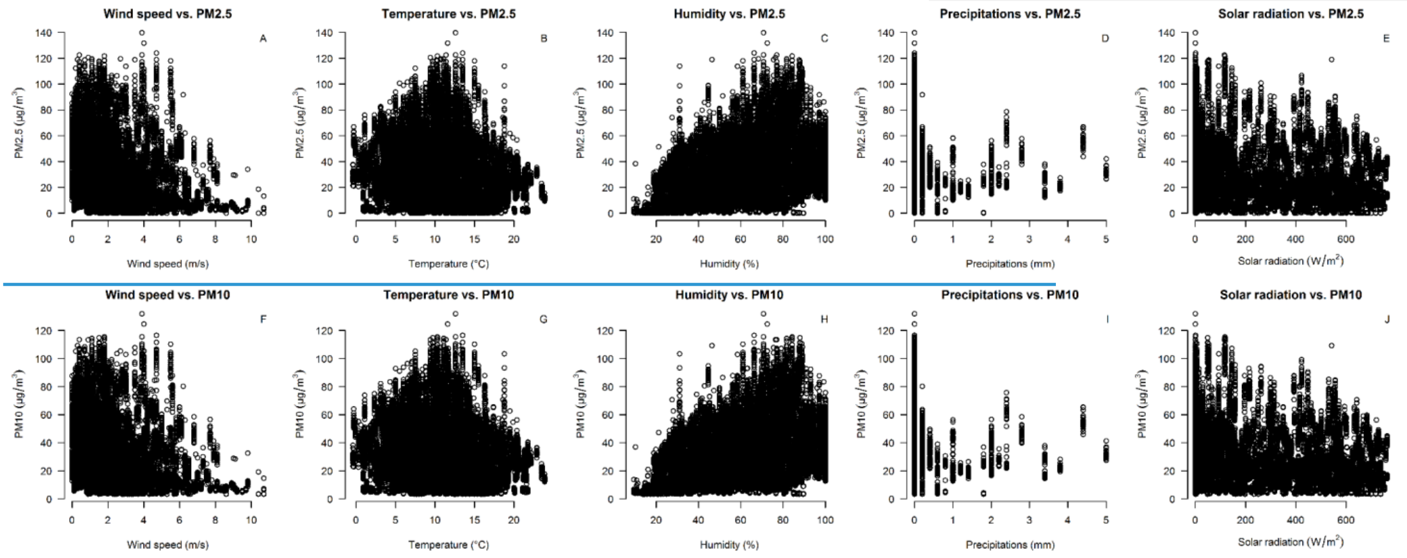

Fig ~~4~~5 – Scatterplots describing the correlation between wind speed, temperature, humidity, precipitations, solar radiation and PM concentration.

Plots in **Fig ~~5A~~ ~~4~~5A** and **Fig ~~5B~~ ~~4~~5F** confirm a mild negative correlation between wind speed and both PM2.5 and PM10 levels (PM2.5: r = ~~-0.40~~ -0.40, 95% CI = ~~-0.41~~ -0.41:-0.39 ~~-0.39~~; PM10: r = ~~-0.40~~ -0.40, 95% CI = ~~-0.41~~ -0.41:~~-0.40~~ -0.39), especially for high wind speed values. High wind speed values could reduce pollutants concentration, representing a potential confounder when comparing PM levels between 2019 and 2020. Further, PM2.5 and PM10 levels correlated positively with humidity (PM2.5: r = 0.47, 95% CI = 0.46:0.48; PM10: r = 0.47, 95% CI = 0.46:0.47, **Fig ~~4~~5C** and **Fig ~~4~~5H**).

W~~A w~~eaker and negative correlation was observed between temperature and pollutants concentration (PM2.5: r = -0.21, 95% CI = -0.22:-0.20; PM10: r = -0.20, 95% CI = -0.21:-0.19, **Fig ~~4~~5B** and **Fig ~~4~~5G**) and between pollutants concentration and solar radiation (PM2.5: r = -0.19, 95% CI = -0.20:-0.18; PM10: r = -0.19, 95% CI = -0.20:-0.18, **Fig ~~4~~5E** and **Fig ~~4~~5J**)~~as shown in Fig 5C and Fig 5D~~.

The evidence of correlation between precipitations and pollutants concentration was almost null (PM2.5: r = 0.02, 95% CI = -0.01:0.03; PM10: r = 0.02, 95% CI = 0.01:0.03, **Fig ~~4~~5D** and **Fig ~~4~~5I**).

Based on the scatterplots in **Fig ~~4~~5** it was possible to observe a non linear relationship between wind speed and pollutants concentration (**Fig ~~4~~5A** and **Fig ~~4~~5F**). ~~Multivariate regression trees were then fitted including both wind speed and temperature as predictors while PM2.5 and PM10 as dependent variables in turn.~~ With the aim ~~The goal was~~ to identify informative wind speed ~~and temperature~~ cut off value~~s~~s able to ~~discriminate~~ distinguish subpopulations of measurements, univariate regression trees were fitted

Formatted: Font: Bold
Formatted: Font: Bold
Formatted: Font: Bold
Formatted: Font: Bold
Formatted: Font: Bold
Formatted: Font: Bold
Formatted: Font: Bold
Formatted: Font: Bold
Formatted: Font: Not Bold
Formatted: Font: Bold
Formatted: Font: Bold
Formatted: Font: Bold
Formatted: Font: Bold
Formatted: Font: Bold
Formatted: Font: Bold

including wind speed as predictor while PM2.5 and PM10 as dependent variables in turn~~characterized by different average PM2.5 and PM10 values~~.

By visual inspection of the cross-validation results of the unpruned trees it was possible to observe that the first split caused a major reduction of the relative cross validation error of about 10% for both pollutants, further splits induced less relevant reductions (~ 2%) as shown in **Supplementary Figure 7~~3~~**. Thus, by imposing a single split to the regression tree algorithm, a wind speed of 2.15 m/s was identified as the most informative threshold to stratify both PM2.5 and PM10 levels.

Pollutants concentration measured when the wind speed was ≥ 2.15 m/s were significantly lower compared to those performed when the wind speed was below the threshold (p-value < 0.0001).

Plots in **Fig 4~~5~~C** and **Fig 4~~5~~H** evidenced that pollutants concentration did not vary with respect to humidity when humidity values were below ~ 20%, highlighting a potential bias in terms of measurements accuracy when the confounder value is below this threshold.

It was then decided to focus on a subset of 22,~~828~~ 240 measures performed when the wind speed was below ~~this threshold~~2.15 m/s and humidity > 20% to avoid ~~its~~ confounding effects.

Formatted: Font: Bold

Formatted: Font: Bold

Formatted: Font: Bold

Formatted: Font: Bold

Matching measurements between years

PM measures were then matched between 2019 and 2020 by sensor, month, day and hour to further reduce the potential impact of confounders when assessing their variation between years. Further, all data from a single sensor (PA-S24) were also removed due to the low number of measurements available compared to the others (n = ~~156~~ 154 vs. n ≥ ~~460~~436). A total number of 5,~~728~~ 452 paired measures (year 2019: 5,~~728~~ 452 measures, year 2020: 5,~~728~~ 452 measures) from 23 sensors were included in the analyses on the basis of the matching criteria. **Supplementary Table ~~1~~ 1** reports the number of measurements available by sensor.

The correlation between PM2.5 and PM10 measures during 2019 and 2020 as well as the correlation between variations in terms of PM2.5 and PM10 between 2019 and 2020 using matched data was extremely high (Spearman r > 0.99), as shown in **Fig 5~~6~~** and **Fig 6~~7~~**.

Formatted: Font: Bold

Formatted: Font: Bold

Formatted: Font: Bold

Formatted: Font: Bold

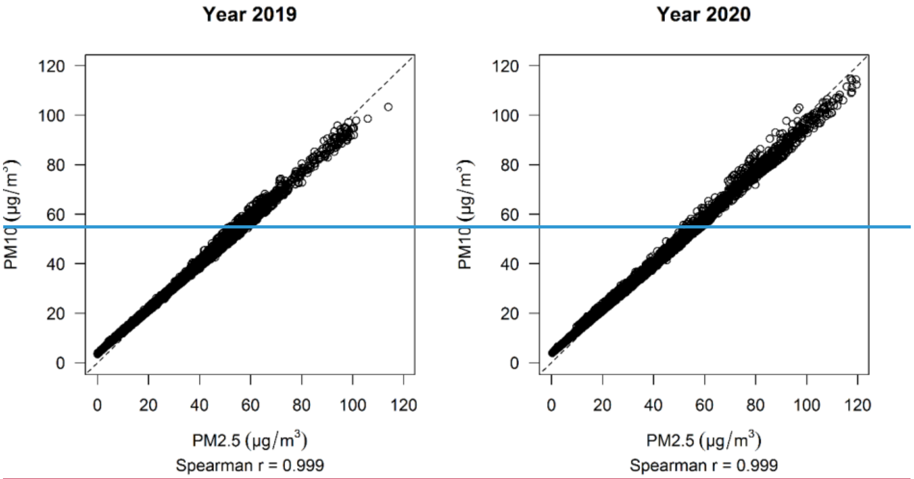

Fig 5~~6~~ – Correlation between PM2.5 and PM10 during years 2019 and 2020.

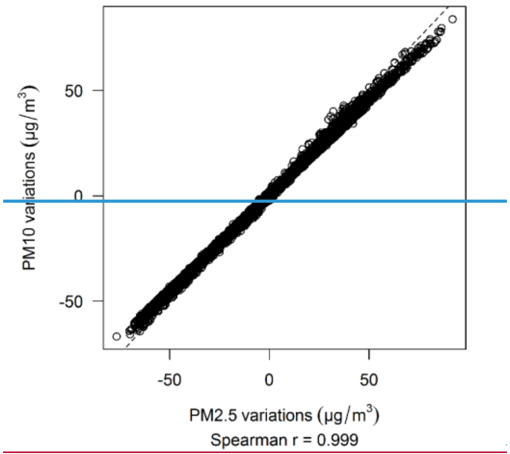

Fig 67 – Correlation between variations in terms PM2.5 and PM10 between years 2019 and 2020.

Variation in terms of pollutants concentration between 2019 and 2020

The variation in terms of PM2.5 and PM10 concentration between 2019 and 2020 has then been estimated, results are reported in **Table 1 2** and show no statistically significant variations in terms of mean PM2.5 (p-value = 0.995177) or PM10 (p-value = 0.904230).

		PM2.5		
**Year**	**N**	**Mean ± SD**	**Median (IQRQ1:Q3)**	**p**
2019	5,452 5,728	34.2 ± 21.0233.24 ± 21.22	34.6 (15.63 : 50.79)33.45 (14.66:50.29)	
2020	5,452 5,728	33.68 ± 23.6933.24 ± 23.71	28.11 (17.55 : 45.12)27.69 (16.73:44.43)	
Variation	5,452 5,728	-0.52 ± 28.560 ± 28.3	-0.38 (-17.92 : 17.33)0.01 (-17.21:17.69)	0.9950.177

		PM10		
**Year**	**N**	**Mean ± SD**	**Median (Q1:Q3IQR)**	**P**
2019	5,452 5,728	34.62 ± 19.1833.76 ± 19.35	34.76 (17.69 : 49.74)33.76 (16.76:49.24)	
2020	5,452 5,728	34.2 ± 21.7633.8 ± 21.78	28.87 (19.5 : 44.18)28.48 (18.91:43.27)	
Variation	5,452 5,728	-0.43 ± 26.190.04 ± 25.95	-0.14 (-16.12 : 15.73)0.22 (-15.74:16.06)	0.904230

Table ~~1~~ 2 – PM2.5 and PM10 distribution during 2019 and 2020. Year = analyzed year or variation between years; Mean ± SD = mean value and standard deviation of pollutants concentration during years 2019, 2020 and variations between 2019 and 2020; Median (**Q1:Q3**~~IQR~~) = median value and ~~IQR~~ 25th : 75th percentiles of pollutants concentration during years 2019, 2020 and variations between 2019 and 2020; p = p – value from the paired samples t-test.

Two ~~A m~~multivariate linear mixed effects model regression models ~~was fit~~have been fitted~~ted~~ to quantify the variation in terms of PM2.5 and PM10 between 2019 and 2020, adjusting by wind speed, temperature, humidity, precipitations, solar radiation, weekend/working days and sensors elevation upon the sea level (fixed effects). A single random effect grouping factor was included, resuming the information about sensor ID and year, month, day, hour when the measure was performed (5,~~728~~ 452 levels or intercept values).

Results are reported in **Table ~~2~~ 3** and show statistically ~~borderline~~ significant ~~increased~~ reductions in terms of PM2.5 and PM10 values during 2020 compared to 2019 accounting for potential confounders (PM2.5: Year (2020) coefficient = ~~+~~ - ~~0.69~~2.58 μg/m^3, 95% CI = -3.25 to -1.91 μg/m^3, p ~~=~~< 0.~~0552~~0001; PM10: Year (2020) coefficient ~~coefficient~~ = ~~+ 0.73~~ - 2.25 μg/m^3, - 95% CI = -2.87 to -1.64 μg/m^3, p ~~=~~< 0.~~0281~~0001).

	PM2.5		PM10	
**Variable**	**Estimate (95% CI)**	**p**	**Estimate (95% CI)**	**p**
~~(Intercept)~~	~~34.12 (29.08:39.16)~~	~~<0.0001~~	~~33.53 (28.91:38.15)~~	~~<0.0001~~
Year (2020)	*-2.58 (-3.25:-1.91)~~0.69 (-0.02:1.4)~~*	*<0.0001~~0.0552~~*	*-2.25 (-2.87:-1.64)~~0.73 (0.08:1.38)~~*	*<0.0001~~0.0281~~*
Working day (yes)	11.15 (10.36:11.94)~~7.27 (6.42:8.12)~~	<0.0001~~<0.0001~~	10.15 (9.42:10.87)~~6.59 (5.81:7.37)~~	<0.0001~~<0.0001~~
Average wind speed (m/s)	-4.33 (-4.99:-3.66)~~-7.75 (-8.45:-7.05)~~	<0.0001~~<0.0001~~	-3.96 (-4.57:-3.36)~~-7.09 (-7.74:-6.45)~~	<0.0001~~<0.0001~~
Temperature (°C)	1.59 (1.48:1.7)~~0.08 (-0.02:0.17)~~	<0.0001~~0.1130~~	1.51 (1.4:1.61)~~0.11 (0.02:0.2)~~	<0.0001~~0.0127~~
Sensors' elevation upon the sea level (m)	0.02 (-0.04:0.08)~~0.02 (-0.04:0.08)~~	0.4850~~0.5246~~	0.03 (-0.03:0.08)~~0.03 (-0.03:0.09)~~	0.3002~~0.3467~~
Humidity (%)	0.54 (0.52:0.57)	<0.0001	0.49 (0.47:0.52)	<0.0001
Precipitations (mm)	-9.55 (-11.2:-7.9)	<0.0001	-8.85 (-10.36:-7.34)	<0.0001
Solar radiation (W/m^2)	0 (-0.01:0)	0.0015	0 (-0.01:0)	0.0001

Table ~~2~~ 3 – Multivariate linear mixed effects model regression: ~~Variation~~ variation in terms of PM2.5 and PM10 between 2019 and 2020 accounting for confounders. Estimate (95% CI) = regression coefficient and 95% CI; p = p-value. The regression coefficient corresponding to "year (2020)" term quantifies the variation in terms of PM2.5 and PM10 pollutants concentration (μg/m^3) between 2019 and 2020 accounting for the other variables included in the model reported in the table.

Variation in terms of pollutants concentration between 2019 and 2020 by daily hours

The presence of differences in terms of PM variations between 2019 and 2020 has been then inspected by daily hour intervals ~~**(Supplementary Table 1)**~~ by including the year x hour interaction ~~in the regression~~and the same set of confounders previously described in the regression. Adjusted means

Formatted
Formatted
Formatted Table
Formatted
Formatted
Formatted
Formatted
Formatted: Font: Italic
Formatted: Font: (Default) Times New Roman, 10 pt, Italian (Italy)
Formatted: Font: (Default) Times New Roman, 10 pt, Italian (Italy)
Formatted: Font: (Default) Times New Roman, 10 pt, Italian (Italy)
Formatted: Font: (Default) Times New Roman, 10 pt, Italian (Italy)
Formatted
Formatted
Formatted
Formatted
Formatted
Formatted
Formatted
Formatted
Formatted
Formatted
Formatted
Formatted
Formatted
Formatted
Formatted
Formatted
Formatted
Formatted
Formatted
Formatted
Formatted
Formatted
Formatted
Formatted: Not Superscript/ Subscript

estimated by multivariate linear mixed effect models showed that both PM2.5 and PM10 variations were characterized by different trends as function of the daily hours interval considered (**Fig** 78 and **Fig** 89).

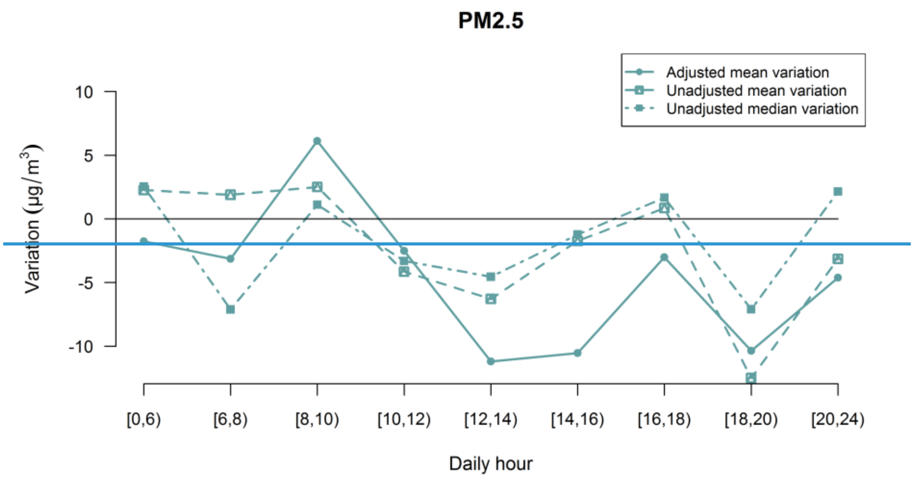

Fig 78 – PM2.5 variations by daily hour interval. Data are presented as adjusted mean variations, unadjusted mean variations and unadjusted median variations.

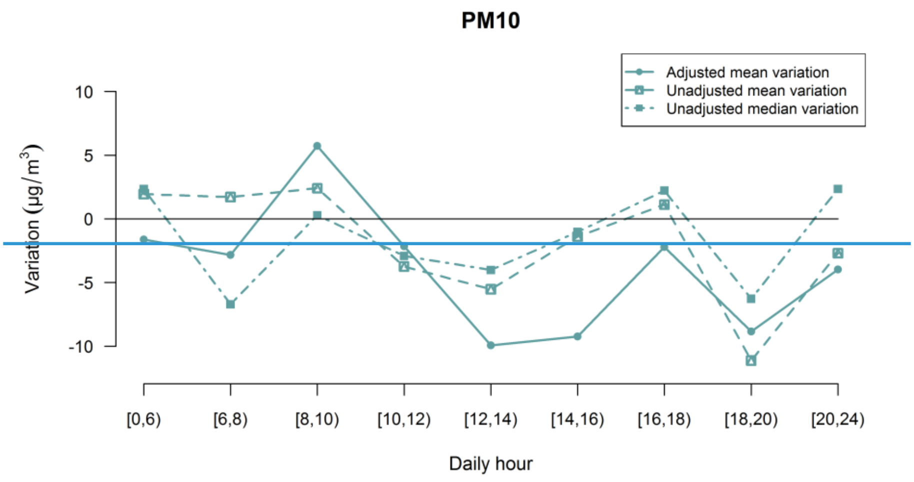

Fig 89 – PM10 variations by daily hour interval. Data are presented as adjusted mean variations, unadjusted mean variations and unadjusted median variations.

Formatted: Font: Bold

Formatted: Font: Bold

Formatted: Font: Bold

Formatted: Font: Bold

Formatted: Left

By inspecting the adjusted mean~~s, unadjusted mean and median~~ variations (**Fig** ~~7~~8 and **Fig** ~~8~~9) it is possible to observe a ~~clear~~ general reduction in terms of PM2.5 and PM10 between 2019 and 2020 ~~from 10:00 to 16:00 and from 18:00 to 20:00~~during all day except from 8 to 10 in the morning, when a pollutants increase was estimated ~~opposed to an increase from 0:00 to 6:00 and from 16:00 to 18:00~~. Unadjusted mean and median variations showed conflicting trends of variations~~The interpretation of the trend during daily hours from 6:00 to 10:00 and from 20:00 to 24:00 resulted more conflicting~~: discrepancies with respect to the adjusted mean variations could be ~~between mean and median values could be~~ partially explained by the non-gaussian distribution of PM measures variations during the different daily hours and by the lack of correction by confounders.

The adjusted variations estimated by linear mixed effect models in the whole sample and by sensor and daily hour (by performing sensor-level analyses) are ~~reported~~ graphically represented in ~~**Table 3**~~**Fig** 9~~10~~ and reported in **Supplementary Table 2** and **Supplementary Table 3** ~~and **Table 4**~~. Results show strong consistency in terms of ~~differences of~~ adjusted mean PM variations by daily hour intervals across sensors. A general pollutants increase was estimated from hour 8 to 10 in the morning for most of the sensors, in some cases increases were observed also from 10 and 12 and - sporadically - during evening/night, morning and afternoon hours.

~~Unadjusted mean and median variations in the whole sample and by sensor and daily hours are reported in **Supplementary Table 2**, **Supplementary Table 3**, **Supplementary Table 4** and **Supplementary Table 5**.~~

Formatted: Font: Bold

Formatted: Font: Bold

Formatted: Font: Bold

Formatted: Font: Bold

Formatted: Font: Bold

Formatted: Font: Bold

Formatted: Font: Not Bold

Formatted: Font: Not Bold

Formatted: Font: Bold

Adjusted mean PM2.5 variations by hour									
Sensor	[0,6)	[6,8)	[8,10)	[10,12)	[12,14)	[14,16)	[16,18)	[18,20)	[20,24)
All sensors	***3.33***	***4.64***	***7.32***	***-1.31***	***-5.39***	***-2.73***	***5.05***	***-4.05***	***-2.29***
PA-S1	6.79	7.08	11.60	1.38	-4.53	-2.40	6.18	-3.19	-0.80
PA-S2	3.34	5.44	7.85	-3.71	-9.16	-4.32	2.88	-3.63	-2.63
PA-S3	4.33	7.28	9.25	1.20	-3.63	-1.28	4.07	-3.48	-3.85
PA-S4	2.16	2.46	5.54	1.46	-3.88	-4.36	3.23	-5.94	-4.11
PA-S5	6.88	7.26	11.44	5.07	-0.37	-0.09	6.80	-2.10	0.20
PA-S6	4.35	4.79	8.15	3.41	-2.45	-2.92	4.73	-4.26	-2.04
PA-S7	11.09	12.31	13.68	3.64	-2.65	1.40	7.40	-0.57	4.30
PA-S8	2.88	4.35	5.68	-2.37	-5.63	-1.15	10.12	-1.50	0.50
PA-S9	-3.71	-2.25	-1.17	-9.74	-9.20	-5.39	2.27	-8.54	-8.98
PA-S10	3.00	4.98	5.36	-3.97	-5.25	-2.59	6.58	-4.93	-3.75
PA-S11	4.39	6.83	8.41	-2.93	-7.74	-2.13	5.28	-4.57	-3.11
PA-S12	2.61	2.19	6.62	-1.43	-5.97	-5.23	1.71	-6.00	-3.01
PA-S13	2.51	4.65	8.37	1.21	-3.93	-3.96	5.66	-5.26	-0.61
PA-S14	1.62	4.45	7.83	-0.41	-3.42	-0.90	8.13	-2.09	0.97
PA-S15	4.03	7.92	10.33	1.07	-2.05	-0.21	6.68	1.72	-3.36
PA-S16	3.12	4.16	7.43	2.26	-3.36	-5.26	0.02	-4.35	-4.70
PA-S17	2.39	3.05	6.17	0.00	-4.54	-2.34	2.98	-3.58	-3.34
PA-S18	3.17	4.90	7.68	-5.00	-6.07	-1.39	8.92	-3.38	-0.64
PA-S19	0.35	0.80	3.63	-2.83	-7.36	-4.22	0.97	-4.95	-5.04
PA-S20	3.85	4.83	7.11	-4.05	-8.13	-2.51	7.81	-5.24	1.03
PA-S21	2.68	3.78	6.83	-4.35	-7.64	-3.10	6.06	-4.47	-0.62
PA-S22	0.33	0.86	2.76	-7.93	-10.81	-6.89	1.91	-7.90	-5.46
PA-S23	4.21	4.47	7.72	-2.50	-6.82	-2.44	5.14	-4.97	-3.61

Formatted: English (United Kingdom)
Formatted: English (United Kingdom)
Formatted: English (United Kingdom)
Formatted: English (United Kingdom)
Formatted: English (United Kingdom)
Formatted: English (United Kingdom)
Formatted: English (United Kingdom)
Formatted: English (United Kingdom)
Formatted: English (United Kingdom)
Formatted: English (United Kingdom)
Formatted: English (United Kingdom)
Formatted: English (United Kingdom)
Formatted: English (United Kingdom)
Formatted: English (United Kingdom)
Formatted: English (United Kingdom)
Formatted: English (United Kingdom)
Formatted: English (United Kingdom)
Formatted: English (United Kingdom)
Formatted: English (United Kingdom)
Formatted: English (United Kingdom)
Formatted: English (United Kingdom)
Formatted: English (United Kingdom)
Formatted: English (United Kingdom)
Formatted: English (United Kingdom)
Formatted: English (United Kingdom)

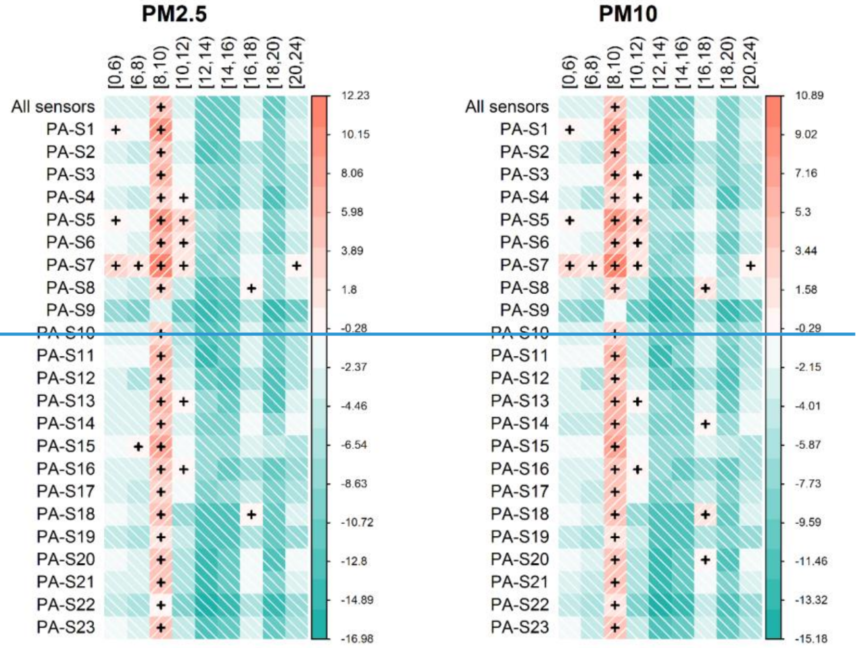

~~**Table 3**~~**Fig 9~~10~~** – Heatmaps showing ~~Adjusted~~ adjusted mean variations in terms of PM2.5 between 2019 and 2020 by sensor and daily hour intervals. Each heatmap row represents graphically the adjusted mean variation in terms of PM2.5 and PM10 between 2019 and 2020 over the complete set of PurpleAir sensors (All sensors) and by ~~PurpleAir (PA) ID corresponds to a different~~ sensor (PA-S1 – PA-S23) over daily hours intervals defined. Shades of ~~In~~ green~~:~~ are proportional to the estimated magnitude of the reduction in terms of PM2.5 between 2019 and 2020; shades of red are proportional to the estimated magnitude of the increase in terms of PM2.5 between 2019 and 2020 as showed by the colors legend on the right side of each plot~~in red: increase in terms of PM2.5 between 2019 and 2020.~~. The "+" symbol denotes an increase in terms of pollutants concentration between year 2019 and 2020.

Formatted: Font: Not Bold

An alternative approach consisting of a modification of the method described by Venter et al. [~~ref~~21] has been also evaluated to assess the impact of the COVID-19 related lockdown on pollutants concentration. According to this method, positive absolute changes in terms of pollutant concentration (observed – expected) indicate an increase in terms of PM compared to the expected values while negative changes a decrease in terms of pollutants concentration compared to the expected values. Positive and negative changes can be attributed to the COVID-19 related lockdown.

Results deriving from this approach were generally concordant with respect to the main findings reported in this section (**Supplementary Table 4**, **Supplementary Figure 8~~4~~**). A negative change in terms of PM2.5 and PM10 was estimated for all sensors during all daily hour intervals except for hours 8 to 10 in the morning, being characterized by a positive change for most sensors for both pollutants. PM10 showed positive changes for some sensors also during hours 10 – 12 while some sensors (PA-S7, PA-S8, PA-S13 and PA-S18) were characterized by a positive change also during afternoon/evening hours.

Formatted: Font: Not Bold

Adjusted mean PM10 variations by hour									
**Sensor**	**[0,6)**	**[6,8)**	**[8,10)**	**[10,12)**	**[12,14)**	**[14,16)**	**[16,18)**	**[18,20)**	**[20,24)**
All sensors	*2.99*	*4.25*	*6.82*	*-0.98*	*-4.46*	*-2.11*	*5.05*	*-3.27*	*-1.88*
PA-S1	5.87	6.23	10.21	1.13	-3.78	-2.00	5.71	-2.74	-0.75
PA-S2	2.81	4.96	7.13	-3.56	-8.35	-3.83	2.92	-3.20	-2.37
PA-S3	3.68	6.33	8.31	1.21	-2.77	-0.65	4.09	-2.74	-3.26
PA-S4	1.77	2.31	5.33	1.80	-2.84	-3.56	3.61	-5.08	-3.65
PA-S5	6.14	6.62	10.48	4.74	0.15	0.35	6.58	-1.47	0.44
PA-S6	4.14	4.61	7.65	3.45	-1.63	-2.13	4.92	-3.40	-1.48
PA-S7	9.55	10.85	12.18	2.93	-2.47	1.28	6.89	-0.63	3.64
PA-S8	2.32	3.68	4.86	-2.37	-5.18	-1.07	9.62	-1.06	0.30
PA-S9	-3.02	-1.67	-0.43	-8.23	-7.70	-4.36	2.76	-7.06	-7.62
PA-S10	2.95	4.69	5.32	-2.86	-4.01	-1.79	6.31	-3.90	-2.77
PA-S11	4.05	6.21	7.91	-2.29	-6.37	-1.36	5.31	-3.55	-2.47
PA-S12	2.52	2.29	6.45	-0.86	-4.81	-4.36	2.20	-5.06	-2.40
PA-S13	2.28	4.22	7.48	1.25	-3.15	-3.16	5.35	-4.42	-0.41
PA-S14	1.29	3.92	7.35	-0.12	-2.61	-0.39	8.03	-1.46	0.97
PA-S15	3.30	6.64	8.93	0.68	-1.99	-0.30	5.78	1.16	-3.12
PA-S16	2.89	3.90	6.93	2.24	-2.59	-4.43	0.48	-3.61	-4.11
PA-S17	2.14	2.89	5.87	0.50	-3.46	-1.65	3.13	-2.86	-2.85
PA-S18	2.59	4.32	6.77	-4.37	-5.31	-1.16	8.26	-2.92	-0.82
PA-S19	0.28	0.78	3.55	-2.42	-6.29	-3.34	1.48	-3.96	-4.37
PA-S20	3.43	4.41	6.61	-3.54	-6.83	-1.78	7.51	-3.64	1.16
PA-S21	2.39	3.44	6.46	-3.75	-6.57	-2.39	6.12	-3.56	-0.32
PA-S22	1.35	1.92	4.08	-6.35	-8.96	-5.25	3.42	-6.07	-3.89
PA-S23	3.76	4.06	7.20	-2.20	-5.75	-1.73	5.24	-4.04	-3.17

Table 4 – Adjusted mean variation in terms of PM10 between 2019 and 2020 by sensor and daily hour intervals. Each PurpleAir (PA) ID corresponds to a different sensor. In green: reduction in terms of PM10 between 2019 and 2020; in red: increase in terms of PM10 between 2019 and 2020.

Formatted: English (United Kingdom)
Formatted: English (United Kingdom)
Formatted: English (United Kingdom)
Formatted: English (United Kingdom)
Formatted: English (United Kingdom)
Formatted: English (United Kingdom)
Formatted: English (United Kingdom)
Formatted: English (United Kingdom)
Formatted: English (United Kingdom)
Formatted: English (United Kingdom)
Formatted: English (United Kingdom)
Formatted: English (United Kingdom)
Formatted: English (United Kingdom)
Formatted: English (United Kingdom)
Formatted: English (United Kingdom)
Formatted: English (United Kingdom)
Formatted: English (United Kingdom)
Formatted: English (United Kingdom)
Formatted: English (United Kingdom)
Formatted: English (United Kingdom)
Formatted: English (United Kingdom)
Formatted: English (United Kingdom)
Formatted: English (United Kingdom)
Formatted: English (United Kingdom)
Formatted: English (United Kingdom)

Discussion

Commented [MA2]: Aggiornare con nuovi risultati e confronto con metodo alternativo.

Air pollution is a complex phenomenon, resulting from the combination of air particles coming from different sources, both artificial and natural. Several different pollutants, including ozone, sulfur dioxide, nitrogen dioxide and particulate matter have an impact on human health. Among these pollutants, particulate matter is often used as a reference to describe air quality, as it includes all particles within a certain diameter (e.g., PM2.5 indicates all particles that have a diameter smaller than 2.5 µm) and it has been demonstrated to have an important relation with diseases risk increase [22~~17~~,23~~18~~]. Reduction of exposure to air pollution is the best prevention strategy that can be applied to reduce the risk of developing respiratory diseases linked to bad air quality. The National Health Ministry of Italy estimated that living in the Po valley, a large plain region in the north of the country, leads to a significant reduction of life expectancy [24~~19~~]. This area is one of the most densely populated in Europe and it is characterized by high pollution levels due to a combination of factors such as high industrial activity, high traffic density, house heating and unfortunate climatic factors. Pollution is generally high during winter months, when cold dense air tends to be pressed to the ground and the frequent presence of fog makes pollutants even more dangerous [25~~0~~]. In order to reduce pollution, the administrations in the regions and the cities affected take several measures like traffic limitations or regulations on the use of house heating every year. However, the results from such restrictions are often not sufficient, as the sources of pollution are numerous, and the combined effects of the produced pollutants are difficult to predict.

With the COVID-19 pandemic, which initially had its European epicenter in northern Italy at the end of winter 2020, a strict lockdown was imposed throughout Italy, as it happened previously in some regions of China and subsequently in the rest of Europe and America, as the COVID-19 cases spread and rose. For several weeks, most of the population was confined at home and all nonessential productive activities were closed. By these measures, a large part of the pollution sources was temporally stopped, creating the ideal ground to perform scientific studies to verify whether the limitation of traffic and shutdown of some activities had an impact on global and local air pollution. A large number of studies aiming at analyzing these aspects were carried out throughout the world, with results varying depending on the geographical areas covered. Some reports showed a global reduction in several pollutants worldwide [26~~1~~], while other local-level studies obtained different results. Wang et al. [27~~2~~] observed a significant reduction in all pollutants in 325 cities in China, especially in the northern cities, whereas Donzelli et al. [28~~3~~] performed a similar analysis over a 7 months period for 3 cities in Tuscany, Italy, and reported different results depending on the pollutant (NO_2 levels changed significantly while PMs did not). A general study performed over western Europe performed by Menut et al. [29~~4~~] taking into account also meteorological variables, showed a general reduction in terms of NO_2 throughout the continent, while the reduction in terms of PMs and O_3 was limited on a general level, but with pronounced local variations. Unlike other areas of the world, a severe reduction of all the pollutants during the lockdown in the Po Valley was not observed [9], indicating that traffic may not have a huge impact on pollution and that meteorological conditions probably play a role even more important than what was usually thought.

In this study, we analyzed the case of Pavia, a city in an area with poor air quality, where a dense network of sensors had been deployed a few months before as part of the European project PULSE, that had the aim to study air quality, among other things, in order to create a collaborative preventive system to help citizens and public health operators to reduce diseases risks and improve quality of life. There are several advantages deriving from the availability of a dense sensors network, such as the possibility to monitor local conditions, estimate pollution levels in each neighborhood and to analyze temporal trends. The choice of the time period considered in the study (i.e., between February and April 2019 and 2020) was motivated by the fact that unique conditions were applied in that period in 2020, as the total lockdown that was instituted had no precedents and was never repeated in the following months. In fact, even if restrictions were kept throughout 2020, the same level of shutdown of all nonessential activities and limitation to all movements were never reached again.

The results of our study show mainly two aspects: first, the influence of climatic factors on PM levels is extremely high, second, considering this difference, there has ~~not~~ been a mildly significant reduction in PM

levels in 2020 during the lockdown, so pollutioneven though pollution levels remained relatively high even without traffic and most productive activities. The latter aspect suggests that most of the relevant pollution sources in this particular geographical area are represented by the heating systems in private houses and commercial traffic, and, even with only essential services active, the peculiar climatic conditions characterized by long periods without wind are able to create dangerous levels of pollution. One interesting result is that looking at the official ARPA data alone, there is not a clear decrease in the PM levels in 2020 if all meteorological variables are not taken into account, whereas there is a definite decrease considering other pollutants. This appears to be in line with other studies conducted over Italy such as [28] and [29]. Considering the variety of pollution sources and local environmental and meteorological situations, the reasons for this are not easy to explain, but it could be assumed that this phenomenon is due to the fact that traffic, which was the major pollution source that was stopped during the lockdown, contributes less to PMs than it does to other pollutants such as SO_2 and CO_2. Furthermore, most PM produced in the Po Valley could come from some sectors of industry and agriculture that were considered essential and therefore were not limited during the lockdown and from house heating systems, especially older ones. Increased house heating could partially explain also the change in the daily patterns that was observed during the lockdown, with a high peak in the first hours of the morning, i.e. the coldest ones in the day, that without lockdowns is balanced by vehicular traffic flowing during rush hours and most productive activities active during all daily hours.

Nevertheless, pollution remains a serious problem in this region, and wide interventions are needed to mitigate health risk related to air quality in northern Italy. Traffic limitations and introduction of new propulsion technologies, such as hybrid or electric engines, certainly contribute to an improvement of this situation, but are not enough to reduce health risk to safe levels.This enlightens the necessity of wide interventions to mitigate the health risks related to air quality in northern Italy. Our results showed also another interesting phenomenon, i.e. a change in the daily air quality patterns during the lockdown, with a higher level of PM during the night opposed to a lower concentration in the day hours. This phenomenon could be related to the increased use of house heating deriving from the fact that people stayed more at home in the considered period in 2020, as the night hours are the coldest ones. Nevertheless, air quality is the outcome of very complex co-occurring factors that are still hard to identify.

Funding

This work has been funded by the European Commission as part of the PULSE project (Grant Agreement GA-727816) and PERISCOPE project (Grant Agreement 101016233), coordinated by the University of Pavia.

References

1. Franchini M, Mannucci PM. Short-term effects of air pollution on cardiovascular diseases: outcomes and mechanisms. J Thromb Haemost. 2007;5(11):2169–74.

2. Aj W. Worldwide trends in asthma morbidity and mortality. Explanation of trends. Bull Int Union Tuberc Lung Dis. 1991 Jun 1;66(2–3):85–9.

3. Pearce N, Aït-Khaled N, Beasley R, Mallol J, Keil U, Mitchell E, et al. Worldwide trends in the prevalence of asthma symptoms: phase III of the International Study of Asthma and Allergies in Childhood (ISAAC). Thorax. 2007 Sep 1;62(9):758–66.

4. Boyle JP, Honeycutt AA, Narayan KMV, Hoerger TJ, Geiss LS, Chen H, et al. Projection of Diabetes Burden Through 2050: Impact of changing demography and disease prevalence in the U.S. Diabetes Care. 2001 Nov 1;24(11):1936–40.

5. Cascella M, Rajnik M, Cuomo A, Dulebohn SC, Di Napoli R. Features, Evaluation and Treatment Coronavirus (COVID-19). In: StatPearls [Internet]. Treasure Island (FL): StatPearls Publishing; 2020 [cited 2020 Jul 8]. Available from: http://www.ncbi.nlm.nih.gov/books/NBK554776/

6. S.r.l LF. Nitrogen dioxide and fine particles are threatening Po valley air quality [Internet]. [cited 2020 Jul 8]. Available from: https://vitesy.com/blog/air-pollution/nitrogen-dioxide-fine-particles-po-valley-air-quality

7. Anjum NA. Good in The Worst: COVID-19 Restrictions and Ease in Global Air Pollution. 2020 Apr 6 [cited 2020 Jul 8]; Available from: https://www.preprints.org/manuscript/202004.0069/v1

8. CNN BRP. People in India can see the Himalayas for the first time in “decades,” as the lockdown eases air pollution [Internet]. CNN. [cited 2020 Jul 8]. Available from: https://www.cnn.com/travel/article/himalayas-visible-lockdown-india-scli-intl/index.html

9. Rita V. Roma e Pianura Padana, le polveri sottili PM10 non diminuiscono con il lockdown [Internet]. Galileo. 2020 [cited 2020 Jul 8]. Available from: https://www.galileonet.it/roma-pianura-padana-polveri-sottili-pm10-non-calano/

10. Inquinamento: cala il biossido di azoto, ma I venti portano le polveri sottili [Internet]. [cited 2021 May 27]. Available from: https://www.repubblica.it/ambiente/2020/03/30/news/meno_biossido_di_azoto_piu_polveri_sottili_cosa_sta_accadendo_nei_nostri_cieli-252701516/

11. Collivignarelli MC, Abbà A, Bertanza G, Pedrazzani R, Ricciardi P, Carnevale Miino M. Lockdown for CoViD-2019 in Milan: What are the effects on air quality? Sci Total Environ. 2020 Aug 25;732:139280.

12. Bigi A, Ghermandi G, Harrison RM. Analysis of the air pollution climate at a background site in the Po valley. J Environ Monit. 2012 Feb 2;14(2):552–63.

13. Pala D, Pagán J, Parimbelli E, Rocca MT, Bellazzi R, Casella V. Spatial Enablement to Support Environmental, Demographic, Socioeconomics, and Health Data Integration and Analysis for Big Cities: A Case Study With Asthma Hospitalizations in New York City. Front Med [Internet]. 2019 Apr 24 [cited 2020 May 3];6. Available from: https://www.ncbi.nlm.nih.gov/pmc/articles/PMC6499162/

14. Pala D, Caldarone AA, Franzini M, Malovini A, Larizza C, Casella V, et al. Deep Learning to Unveil Correlations between Urban Landscape and Population Health. Sensors [Internet]. 2020 Apr 8 [cited 2020 May 3];20(7). Available from: https://www.ncbi.nlm.nih.gov/pmc/articles/PMC7181035/

15. Kumar P, Morawska L, Martani C, Biskos G, Neophytou M, Di Sabatino S, et al. The rise of low-cost sensing for managing air pollution in cities. Environ Int. 2015 Feb 1;75:199–205.

16. contributors PL. PurpleAir [Internet]. Public Lab. [cited 2020 May 3]. Available from: https://publiclab.org/n/16947

17. ARPA Lombardia – Taratura degli strumenti. [Internet]. [cited 2021 Jul 31]. Available from: https://www.purpleair.com/map?opt=1/mAQI/a10/cC0#12.43/45.19247/9.16417

18. Real-time air quality map | Purple Air [Internet]. [cited 2021 Aug 06]. Available from: https://www.arpalombardia.it/Pages/Aria/Rete-di-rilevamento/Qualit%C3%A0-dei-dati/Taratura-degli-strument.aspx?firstlevel=Rete%20di%20rilevamento

19. Qualità dell'aria? In Italia è migliorata negli ultimi cinque anni. Cosa misura in Snpa. [Internet]. [cited 2021 Jul 30]. Available from: https://www.infodata.ilsole24ore.com/2021/01/31/qualita-dellaria-italia-migliorata-negli-ultimi-cinque-anni-cosa-misura-snpa/?refresh_ce=1

20. Qualità dell'aria, trend in miglioramento negli ultimi 5 anni. [Internet]. [cited 2021 Jul 30]. Available from: https://www.snpambiente.it/2021/01/29/qualita-dellaria-in-italia-trend-in-miglioramento-negli-ultimi-5-anni/

21. Venter ZS, Aunan K, Chowdhury S, Lelieveld J. COVID-19 lockdowns cause global air pollution declines. Proc Natl Acad Sci U S A. 2020;117(32):18984-18990. doi:10.1073/pnas.2006853117

22~~17~~. Fan J, Li S, Fan C, Bai Z, Yang K. The impact of PM2.5 on asthma emergency department visits: a systematic review and meta-analysis. Environ Sci Pollut Res. 2016 Jan 1;23(1):843–50.

23~~18~~. Tecer LH, Alagha O, Karaca F, Tuncel G, Eldes N. Particulate Matter (PM2.5, PM10-2.5, and PM10) and Children's Hospital Admissions for Asthma and Respiratory Diseases: A Bidirectional Case-Crossover Study. J Toxicol Environ Health A. 2008 Mar 12;71(8):512–20.

24~~19~~. redazione. Air pollution shortens the life expectancy of Italians [Internet]. Science in the net. 2015 [cited 2020 Jul 8]. Available from: http://www.scienceonthenet.eu/content/article/editorial-staff/air-pollution-shortens-life-expectancy-italians/june-2015

25~~0~~. Cai D-P, He Y-M. Daily lifestyles in the fog and haze weather. J Thorac Dis. 2016 Jan;8(1):E75–7.

26~~1~~. Zander SV, Kristin A, Sourangsu C, Jos. COVID-19 lockdowns cause global air pollution declines. Proceedings of the National Academy of Sciences. Aug 2020, 117(32):18984-18990; DOI: 10.1073/pnas.2006853117

27~~2~~. Wang M, Liu F, & Zheng M. Air quality improvement from COVID-19 lockdown: evidence from China. Air Qual Atmos Health. 2020. https://doi.org/10.1007/s11869-020-00963-y

28~~3~~. Donzelli G, Cioni L, Cancellieri M, Llopis Morales A, Morales Suárez-Varela MM. The Effect of the Covid-19 Lockdown on Air Quality in Three Italian Medium-Sized Cities. Atmosphere. 2020; 11(10):1118. https://doi.org/10.3390/atmos11101118

29~~4~~. Laurent M, Bertrand B, Guillaume S, Sylvain M, Romain P, Arineh C. Impact of lockdown measures to combat Covid-19 on air quality over western Europe. Science of The Total Environment. 2020; 741. https://doi.org/10.1016/j.scitotenv.2020.140426.

Supporting Information

Formatted: Italian (Italy)

Field Code Changed

Formatted: Italian (Italy)

Formatted: Italian (Italy)

Formatted: English (United States)

Formatted: Italian (Italy)

Formatted: Italian (Italy)

Formatted: English (United States)

Formatted: Italian (Italy)

Formatted: Space After: 0 pt

Formatted: Justified, Indent: Left: 0", First line: 0", Space After: 8 pt, Line spacing: Multiple 1.08 li, Border: Top: (No border), Bottom: (No border), Left: (No border), Right: (No border), Between : (No border), Tab stops: Not at 0.27"

Formatted: Font: 12 pt, Font color: Auto

Formatted: English (United States)

Formatted: English (United States)

S1 Fig. PM10 correlation matrix. Correlation matrix (Pearson's correlation) of the PM10 measurements performed by seven Purple Air sensors co-located on a selected day.~~**S1 Fig. PM10 correlation matrix. Correlation matrix of the PM10 measurements performed by 7 Purple Air sensors co-located on a selected day.S2 Fig. PM2.5 correlation matrix. Correlation matrix of the PM2.5 measurements performed by seven Purple Air sensors co-located on a selected day**~~

S2 Fig. PM2.5 correlation matrix. Correlation matrix (Pearson's correlation) of the PM2.5 measurements performed by seven Purple Air sensors co-located on a selected day.

S3 Fig. Range of the PM10 values measured by the seven co-located sensors.

S4 Fig. Range of the PM2.5 values measured by the seven co-located sensors.

S5 Fig. PM10 and Wind Variations in time in the considered periods in 2019 and 2020. The upper plot shows 2019 data, the lower plot shows 2020 data.

S6 Fig. PM10 and Temperature Variations in time in the considered periods in 2019 and 2020. The upper plot shows 2019 data, the lower plot shows 2020 data.

S7 Fig. Visual representation of the cross-validation results for PM2.5 and PM10. The x-axis represents the complexity parameter corresponding to different tree sizes while the y-axis represents the cross validation relative error.

S8 Fig. Heatmaps showing the median difference between observed and LMM - predicted PM2.5 and PM10 during 2020 by sensor and daily hour intervals. Each heatmap row graphically represents the median absolute differences between observed and predicted pollutants measurements by daily hours intervals. Shades of green indicate negative absolute differences between observed and predicted pollutants concentration during 2020, shades of red indicate positive absolute differences between observed and predicted pollutants concentration during 2020 as showed by the colour code legend on the right side of each plot. The "+" symbol denotes a positive absolute differences between observed and predicted pollutants concentration.

S1 Table. Number of paired measurements available by sensor and hours.

S2 Table. Adjusted mean variations in terms of PM2.5 between 2019 and 2020 by sensor and daily hours. Each PurpleAir (PA) ID corresponds to a different sensor. In green: reduction in terms of PM2.5 between 2019 and 2020; in red: increase in terms of PM2.5 between 2019 and 2020.

S3 Table. Adjusted median variations in terms of PM10 between 2019 and 2020 by sensor and daily hours. Each Purple Air (PA) ID corresponds to a different sensor. In green: reduction in terms of PM10 between 2019 and 2020; in red: increase in terms of PM10 between 2019 and 2020.

S4 Table. Root mean square error, mean absolute error and Pearson correlation coefficient r from the LMM regression method.

~~**S3 Fig. Range of the PM10 values measured by the seven co-located sensorsS4 Fig. Range of the PM2.5 values measured by the seven co-located sensorsS5 Fig. PM10 and Wind Variations in time in the considered periods in 2019 and 2020. The upper plot shows 2019 data, the lower plot shows 2020 dataS6 Fig. PM10 and Temperature Variations in time in the considered periods in 2019 and 2020. The upper plot shows 2019 data, the lower plot shows 2020 dataS7 Fig. Visual representation of the cross-validation results for PM2.5 and PM10. The x-axis represents the complexity parameter corresponding to different tree sizes while the y-axis represents the cross validation relative error**~~

~~**S8 Fig. Heatmaps showing the median difference between observed and LMM - predicted PM2.5 and PM10 during 2020 by sensor and daily hour intervals. Each heatmap row graphically represents the median absolute differences between observed and predicted pollutants measurements by daily hours intervals. Shades of green indicate negative absolute differences between observed and predicted pollutants concentration during 2020, shades of red indicate positive absolute differences between observed and predicted pollutants concentration during 2020 as showed by the colour code legend on the right side of each plot. The "+" symbol denotes a positive absolute differences between observed and predicted pollutants concentrationS1 Table. Number of paired measurements available by sensor and hoursS2 Table. Adjusted mean variations in terms of PM2.5 between 2019 and 2020 by sensor and daily hours**. Each PurpleAir (PA) ID corresponds to a different~~

Formatted: Font: Not Bold

Formatted: Justified

Formatted: Justified

Formatted: Indent: Left: 0", First line: 0"

sensor. In green: reduction in terms of PM2.5 between 2019 and 2020; in red: increase in terms of PM2.5 between 2019 and 2020**S3 Table. Adjusted median variations in terms of PM10 between 2019 and 2020 by sensor and daily hours**. Each Purple Air (PA) ID corresponds to a different sensor. In green: reduction in terms of PM10 between 2019 and 2020; in red: increase in terms of PM10 between 2019 and 2020**S4 Table.** Root mean square error, mean absolute error and Pearson correlation coefficient r from the LMM regression method.

Formatted: English (United Kingdom)

We would like to thank the Reviewer for the positive comments on our study and for raising interesting points regarding our research. The comments have been very beneficial to improve the quality of the paper and better conveying its message. It is true that there is considerable interest on the effect of the lockdown on air pollution, as the unique situation of last year caused an unprecedented opportunity to explore the real impact of traffic and some productive activities on air quality. The phenomenon is really complex, as it is influenced by a large number of external factors.

In this revision process, we addressed all the points of the review, adding new analyses and editing the manuscript where required. Concerning the list of comments, the detailed modifications are the following:

1) ***Detailed information on the sensor calibration: how each of the sensors are calibrated? Are all the sensors been co-located at one of the ARPA stations? Does the performance of the sensor change with time, and how this is controlled? How do the sensors compare with each other?***

   Purple Air sensors are already calibrated during their production before being made available for purchase, as stated in the official documentation. To increase the precision of their measurements, we applied a simple recalibration method, explained in lines 176-204 of the manuscript, where we used the data coming from the official ARPA sensors as reference, since those sensors are calibrated with reliable procedures according to the European standards (see point 3). A parallel study that was performed at the University of Pavia showed that co-located Purple Air sensors measure PMs with high correlation, and therefore that the sensors are expected to provide consistent results between each other. For this reason we used only one sensor colocated with the ARPA one to estimate the correction to be made, and then applied it to all the other sensors. Considering that Pavia is a small city with no significant climatic differences within different zones, we assume that a possible change in performance over time does not affect some sensors more than others. The calibration process is repeated every year. We added a more detailed explanation in lines 194-203 and reported the results of the co-location studies in supplementary figures 1-4.

2) Time series figures or raw data for each sensor should be provided, perhaps as a supplementary table or dataset. It may be easier to separate the sensor location into urban background, traffic and rural background (if any) and present the average data. If so, the original raw data should also be provided.

The raw sensor data will be provided on a GitHub folder, both for the Purple Air sensors and the ARPA ones. Pavia is a small city and does not have significant changes in urban landscape, as there are not extremely urbanized areas and industrial sites inside the city borders. We also did not install sensors in rural areas (which are outside of the municipality borders). Nevertheless, the central area of the city is mostly closed to traffic, so we divided the sensors according to their locations into two categories: center and traffic areas. These categories have been added to the raw data. Most sensors belong to the traffic areas category and significant differences in the data from the two categories were not observed.

3) What data are available from the ARPA stations and how they are measured? It mentioned ARPA sensors – what are they?

ARPA Lombardia (Environmental Protection Regional Agency of the region of Lombardy) is a public agency that has the aim of measuring and treating environmental data of the region the

city of Pavia is located in. Through numerous sensors scattered throughout the region, ARPA collects a large quantity of data about air pollution, meteorology, agriculture, sole status etc.

In Pavia, there are two official ARPA air quality monitoring stations, they are high-quality fixed stations that measure several pollutants (NOX, SO2, CO, O3, PM10 and PM2.5) at regular time intervals. These sensors are calibrated continuously using other commercially available instruments that can be used as reference according to Italian or European laws (https://www.arpalombardia.it/Pages/Aria/Rete-di-rilevamento/Qualit%C3%A0-dei-dati/Taratura-degli-strument.aspx?firstlevel=Rete%20di%20rilevamento) and therefore can be considered very reliable. Weather parameters are collected as well, and data is freely available upon request on a dedicated portal.

We added this information in the manuscript, lines 132-149.

4) Figure 1 – short-term air pollution levels are highly dependent on meteorology. Is it really meaningful to compare the concentration of PM10 levels in late Feb to March in 2020 with that in 2019? What is interesting is that there are large differences for the two stations (2020 vs 2019). It would be important to explain why this is the case. The quality of the figure is not so good but this can be rectified at a later stage. You may want to put the two figures into one.

It is true that pollution levels are highly dependent on meteorology, and unfortunately spring months like March are the most meteorologically unstable months in which wind, humidity, pressure etc. can vary very quickly and influence air pollution's measurements. The differences between 2019 and 2020 are due to this variability, as 2019 had a warmer spring mostly dominated by high pressure systems and higher temperatures, whereas 2020 had a generally lower temperature and higher instability. The aim of this image is to show the effects of this variability and prove that meteorology can affect the measurements in a significant way. Since the lockdown was established in this period, comparing data in this unstable month was not a choice, but our analyses explicitly consider the effects of meteorological variables to avoid confounding factors (see also point 6). Comments about this have been added in lines 268-271.

5) Line 220 – this should be in the methodology?

We thank the Reviewer for the suggestion and moved it to the Methods section (lines 203-208).

6) Line 226 – this section explored the effect of meteorology on air pollution levels. There are a number of figures but the key messages could be made clearer. Why only wind speed and temperature? Other meteorological conditions such as wind direction, relative humidity, cloud cover, precipitation, and back trajectories all could contribute to the variations in air quality. The key point of this section is to establish the regression models so that you can predict the concentrations under same meteorological conditions whether that is in 2020 or 2019. Although this regression model is not as good as machine learning based techniques, such as based on random forest algorithms (you should be able to find relevant papers), it has been used by Zenter et al. (https://www.pnas.org/content/pnas/117/32/18984.full.pdf). Only by doing this, you can compare the data in 2019 with that in 2020.

We thank the Reviewer for highlighting these aspects: we updated the analyses by including additional potential confounding factors in the models and tested an additional methodology to estimate the impact of the lock-down on pollutants concentration.

The set of confounders considered in the analyses now include: wind (m/s), temperature (°C), humidity (%), precipitations (mm) and solar radiation (W/m^2). The correlation between the updated set of variables and PM2.5 and PM10 concentrations has been extensively explored as described in the Results section with title "*Effect of potential confounders on pollutants concentration*". In particular, an updated approach to data filtering has been applied as reported in the following. Based on the scatterplots in Fig 6 we observed a non-linear relationship between wind speed and pollutants concentration (Fig 4A and Fig 4F). With the aim to identify informative wind speed cut off values able to distinguish subpopulations of measurements, univariate regression trees were fitted including wind speed as predictor while PM2.5 and PM10 as dependent variables in turn. By imposing a single split to the regression tree algorithm, a wind speed of 2.15 m/s was identified as the most informative threshold to stratify both PM2.5 and PM10 levels. Further, plots in Fig 4C and Fig 4H evidenced that pollutants concentration did not vary with respect to humidity when humidity values were below ~ 20%, highlighting a potential bias in terms of measurements accuracy when the confounder value is below this threshold. It was then decided to focus on measures performed when the wind speed was below 2.15 m/s and humidity > 20% to avoid confounding effects.
As an additional approach to estimate the effect of the lock-down on pollutants concentration, a methodology based on the method described in Venter et al. (https://www.pnas.org/content/pnas/117/32/18984.full.pdf) has been applied as described in the Supplementary Data section. In details, the implemented methodology consists of the following steps:

a) Train linear mixed model regression models (LMM) using data from 2019 at a sensor level, using PM2.5 and PM10 levels in turn as dependent variable and the following predictors as independent variables: working day, wind, temperature, humidity, precipitations, solar radiation, day/month, daily hour categories. The day/month information is used as random effect grouping variable, while the remaining ones as fixed terms.
b) Apply the LMM model trained on data from 2019 to forecast PM2.5 and PM10 concentrations during 2020.
c) Using the predicted pollutants concentration as benchmark, compare the predicted and the observed PM2.5 and PM10 values to estimate the absolute change (observed – predicted) in terms of pollutants concentration. Positive changes indicate an increase in terms of pollutants concentration compared to the expected values, negative changes a decrease in terms of pollutants concentration compared to the expected values.

The Pearson correlation coefficient *r* between observed and predicted PM2.5 and PM10 concentrations during 2020 was + 0.51 for PM2.5 and + 0.52 for PM10 (Supplementary Table 4). The median value of the absolute differences between observed and predicted pollutants concentration by sensor and daily hour showed trends concordant with what estimated by the method used in our manuscript (Supplementary Figure 8 vs. Figure 9).

7) Table 2: do not understand this. For example, what does intercept mean?

Table 2 reports the regression coefficients, 95% confidence intervals and p-values corresponding to the set of variables included in the multivariate linear mixed effects model regression fitted to estimate the mean variation in terms of PM2.5 and PM10 between 2019 and 2020 accounting for confounders. The regression coefficient corresponding to the "year (2020)" term quantifies the average variation in terms of PM2.5 and PM10 pollutants concentration between 2019 and 2020 accounting for potential confounding effect of the other variables included in the model and reported in the table. For sake of clarity the coefficients and significance corresponding to the intercept term have been now removed from Table 2. These aspects have been described more in the detail in Table 2 legend and in the corresponding text section.

8) Section starting line 308: this is interesting. Consider putting data in Table 3 and 4 in the SI and present the data in figures. It would be much easier to see the trend.

We made the proposed change, and we thank the Reviewer for the suggestion. Table 3 and Table 4 have been moved to Supplementary Data section (Supplementary Table 2 and Supplementary Table 3) and replaced by the two Heatmaps in Figure 9 graphically resuming the same information.

9) Is there longer-term changes in PM levels in the study region? For example, are the PM levels reducing in the previous years? If so, then the PM levels will be lower in 2020 whether or not there is a lockdown. You may need to take this "trend" into account as well. This is called "detrend".

There is some evidence of a reduction trend in all the area in the last years (https://www.infodata.ilsole24ore.com/2021/01/31/qualita-dellaria-italia-migliorata-negli-ultimi-cinque-anni-cosa-misura-snpa/?refresh_ce=1), although with notable fluctuations. Looking at the data gathered by the Italian National Environmental Protection System, it can be noticed that the reduction trend appears less evident after 2018, with even a little increase, probably not significant, in the PM10 concentrations in 2020. The article itself states that the meteorological variability could have played an important role in the measurements' variations, as in 2019 and 2020 temperatures were generally higher and precipitations lower than the previous years. Therefore, we do not assume that the general trend of the last years could influence the difference in PM2.5 and PM10 concentrations between 2019 and 2020. We added a few lines about this (lines 210-224) with the proper citations.

10) Discussions – this seems very general. It would be important to explain your results, e.g., why there is no obvious change in PM levels? We know the emission reduced in 2020 as a result of the lockdown. Is it due to the sensor uncertainty, or meteorology difference in 2019 and 2020, or is it due to the negligible contribution to PM levels from road traffic? Or is it due to changing chemistry? Do you see variations in different types of sites, for example, do you see changes in PM levels at roadside sites but not at the urban background sites? The changing diurnal patterns are really interesting and suggest different emission sources. Are there any other data, such as NO2, CO, BC data from the monitoring stations that can potentially help to interpret the results better? The second part of the discussion should focus on the implications of the results for air pollution control in the study region.

The discussion has been extended according to the new results and the Reviewer's comments, especially from line 480.
