# Supplementary material for: Impact of COVID-19 lockdown on PM concentrations in an Italian Northern City: A year-by-year assessment
Source: PLoS One. 2022 Mar 28;17(3):e0263265. doi: 10.1371/journal.pone.0263265 (PMC8959169; doi:10.1371/journal.pone.0263265)
Supplement: Attachment — Submitted filename: Response to Reviewers.pdf [file pone.0263265.s019.pdf]

We would like to thank the Reviewers for revising our manuscript and suggesting useful improvements. We tried to address each point editing the manuscript in the best possible way following the suggestions. Our specific modifications and comments are listed below:

Editor's Comments:

There are major presentational issues. The overall English presentation is very poor with numerous English errors.

We double checked the manuscript again and corrected some mistakes.

There are two many figures and some could be moved to the SI.

We moved Figures 3,6 and 8 to the SI.

The Discussions are very poorly written. A majority of such discussions should be in the Introduction. Discussions should include:

1) Why you have seen a reduction in 2020: you should look at literature data and emission inventory to see what are the main sources of particles; you can then explain your data by linking to mobility changes - which leads to lower levels of PM / NOx emissions.

2) Why you did not see a reduction in 2020 in raw data - explain which meteorological factor(s) contributed to this. Basically you results showed that meteorological conditions in 2020 was not as good as in 2019, so the observed concentrations are higher than those if under 2019 meteorological conditions.

3) What are the implications of your results: something like this; Such a lockdown did not change PM levels much - so more efforts should be focused on sources other than traffic.

We thank the Reviewer from the suggestion, our discussion was in fact too much focused on a general discourse rather than an accurate dissertation on our findings and analysis. We modified the entire discussion section, deleting the first paragraph and going deeper on the comments concerning our analyses.

#Reviewer 1

We thank the Reviewer for the appreciation of our paper and the useful comments. We addressed all the minor issues as follows:

1) Line 70, I do not think that precipitation could disperse air pollutants.

That is true, precipitation does not disperse pollutants alone, although long lasting rain can push them to the ground. Anyway, we removed the sentence from the paper.

2) The 10 and 2.5 in terms PM10 and PM2.5 should be subscript.

We edited the text and made them subscripts.

3) Line 126, I suggest the authors add one more sentence to describe the measurement principle of PurpleAir sensors. Is it based on laser measurement?

We added a description of the measurement principle as suggested.

4) Figure 1, the word is too small to read. Captions of X-axis and Y-axis are missing.

We modified Figure 1 and increased the labels dimension.

5) Line 274 and Table 1, what is SO?

This was a typo, what we actually meant was NO. We thank the reviewer for making us notice it.

6) Figure 4, the word is too small to read.

We modified Figure 4 and increased the labels dimension.

7) Figures 5 and 6 could be combined as one figure.

In order not to decrease the dimension of the figures too much and make them difficult to analyze, we moved figure 6 in the SI.

8) The first paragraph in Discussion is unnecessary or should not be in Discussion section. Also, from my perspective, the discussion is weak. I would expect more discussion based on their own data and literatures.

We removed the first paragraph and, following also the other Reviewer's suggestions, edited the discussion section adding more insights on our results.
